# The effects of coconut oil on the cardiometabolic profile: a systematic review and meta-analysis of randomized clinical trials

**DOI:** 10.1186/s12944-022-01685-z

**Published:** 2022-08-31

**Authors:** Ana Cláudia Duarte, Bernardo Frison Spiazzi, Carolina Pires Zingano, Eduarda Nunes Merello, Laura Fink Wayerbacher, Paula Portal Teixeira, Laura Penso Farenzena, Carina de Araujo, Carmen Raya Amazarray, Verônica Colpani, Fernando Gerchman

**Affiliations:** 1 Post-Graduate Program in Medical Sciences: Endocrinology, Universidade Federal Do Rio Grande Do Sul, Porto Alegre, Brazil; 2 Faculdade de Medicina, Departamento de Medicina Interna, Universidade Federal Do Rio Grande Do Sul, Porto Alegre, Brazil; 3 Division of Endocrinology and Metabolism, Hospital de Clinicas de Porto Alegre, Porto Alegre, Brazil; 4 Research Projects Office, Hospital Moinhos de Vento, Porto Alegre, Brazil; 5 Division of Endocrinology and Metabolism, Hospital Moinhos de Vento, Porto Alegre, Brazil

**Keywords:** Coconut oil, Saturated fatty acids, Lipid profile, Anthropometric profile

## Abstract

**Background:**

Despite having a 92% concentration of saturated fatty acid composition, leading to an apparently unfavorable lipid profile, body weight and glycemic effect, coconut oil is consumed worldwide. Thus, we conducted an updated systematic review and meta-analysis of randomized clinical trials (RCTs) to analyze the effect of coconut oil intake on different cardiometabolic outcomes.

**Methods:**

We searched Medline, Embase, and LILACS for RCTs conducted prior to April 2022. We included RCTs that compared effects of coconut oil intake with other substances on anthropometric and metabolic profiles in adults published in all languages, and excluded non-randomized trials and short follow-up studies. Risk of bias was assessed with the RoB 2 tool and certainty of evidence with GRADE. Where possible, we performed meta-analyses using a random-effects model.

**Results:**

We included seven studies in the meta-analysis (*n* = 515; 50% females, follow up from 4 weeks to 2 years). The amount of coconut oil consumed varied and is expressed differently among studies: 12 to 30 ml of coconut oil/day (*n* = 5), as part of the amount of SFAs or total daily consumed fat (*n* = 1), a variation of 6 to 54.4 g/day (*n* = 5), or as part of the total caloric energy intake (15 to 21%) (*n* = 6). Coconut oil intake did not significantly decrease body weight (MD -0.24 kg, 95% CI -0.83 kg to 0.34 kg), waist circumference (MD -0.64 cm, 95% CI -1.69 cm to 0.41 cm), and % body fat (-0.10%, 95% CI -0.56% to 0.36%), low-density lipoprotein cholesterol (LDL-C) (MD -1.67 mg/dL, 95% CI -6.93 to 3.59 mg/dL), and triglyceride (TG) levels (MD -0.24 mg/dL, 95% CI -5.52 to 5.04 mg/dL). However, coconut oil intake was associated with a small increase in high-density lipoprotein cholesterol (HDL-C) (MD 3.28 mg/dL, 95% CI 0.66 to 5.90 mg/dL). Overall risk of bias was high, and certainty of evidence was very-low. Study limitations include the heterogeneity of intervention methods, in addition to small samples and short follow-ups, which undermine the effects of dietary intervention in metabolic parameters.

**Conclusions:**

Coconut oil intake revealed no clinically relevant improvement in lipid profile and body composition compared to other oils/fats. Strategies to advise the public on the consumption of other oils, not coconut oil, due to proven cardiometabolic benefits should be implemented.

**Registration:**

PROSPERO CRD42018081461.

**Supplementary Information:**

The online version contains supplementary material available at 10.1186/s12944-022-01685-z.

## Background

Cardiovascular disease (CVD), particularly coronary heart disease and stroke, is a major public health problem, being responsible for one-third of deaths worldwide [1–4]. Despite the great effort of different scientific organizations to fight against the burden of major risk factors for CVD, it is estimated that 11 million deaths and 255 million disability-adjusted life-years are attributable to dietary risk factors [5–8].

The impact of different types of dietary fats on health has been studied, and its contribution to the development of diseases, causing major burden, such as diabetes, cardiovascular diseases and cancer has been debated [6, 9]. A recent report from the American Heart Association based on different prospective cohort studies, randomized clinical trials (RCTs), and meta-analyses estimated that replacing 5% of energy intake of saturated fatty acids (SFAs) with the same intake of polyunsaturated fatty acids (PUFAs) or monounsaturated fatty acids (MUFAs) was associated with a 25 and 15% lower risk of coronary heart disease, respectively [6]. In light of this evidence, the most recent Dietary Guidelines for Americans recommend a reduction in SFAs to less than 10% of calories and their replacement with unsaturated fats [10]. Additionally, recent data from long-term prospective cohorts and meta-analyses have shown that these recommendations are associated with weight gain prevention and reduction of insulin resistance and risk for diabetes [11–15].

Despite that, coconut oil, which is more than 90% SFA, has been widely recommended on social media for the management of obesity, diabetes, and lipid disorders, broadening its consumption all over the world [16–18]. In increasing demand, the estimated consumption of coconut oil in the United States reached 400,000 tons in 2010 [19]. Nonetheless, before the recent rise in coconut oil consumption in western countries, it was only mainly present in some Asian populations’ diets [20–22].

A recent systematic review showed that lauric, myristic, and palmitic fatty acids—the major components of coconut oil—are responsible for the highest increase in low-density lipoprotein cholesterol (LDL-C) levels, which is a major risk factor for CVD [19]. Unlike other types of oils which were consistently proven to prevent weight gain, diabetes, CVD, and mortality [23–25], studies that analyzed how coconut oil intake affects weight, lipid and glycemic levels are mostly based on small, short-term observational studies and clinical trials [16, 26, 27]. In addition, there are meta-analyses including RCTs that have even demonstrated that coconut oil intake increased LDL-C in comparison to non-tropical vegetable and animal oils and did not observe differences in TG levels [28, 29].

Due to the popularity of coconut oil as a “healthy” food, its broad dietary consumption has risen all over the world. This has led to increasing difficulties to translate medical and nutritional science into adequate recommendations for physicians and health workers as well as laymen. Given this context, we conducted an updated systematic review and meta-analysis of RCTs investigating the effects of coconut oil intake on body weight and composition, lipid profile, glycemic status, blood pressure, and subclinical inflammation in adults.

## Methods

This systematic review and meta-analysis was prospectively registered on PROSPERO (CRD42018081461) and was conducted in accordance with the Preferred Reporting Items for Systematic Reviews and Meta-Analyses (PRISMA) 2020 statement [30].

### Search methods for the identification of studies

We searched the MEDLINE, EMBASE, and LILACS databases to identify studies analyzing the effects of coconut oil intake on weight, lipid and glycemic profiles, blood pressure, and subclinical inflammation in adults from inception to April, 2022, and searched www.clinicaltrials.gov for potentially available unpublished results (Supplementary Appendix I). The references of relevant systematic reviews were screened manually to identify further relevant citations. When an article did not present the results of interest, we contacted the authors by email requesting the data.

### Study selection and data extraction

Study inclusion and data extraction were conducted independently (A.C.D., C.R.A., and C.A.). Reviewers resolved discrepancies by discussion and, when necessary, with adjudication by a third party (F.G.). Inter-rater agreement was assessed using the Kappa statistic and percentage of agreement. Kappa statistic was calculated with SPSS software (version 18.03; Chicago, USA). Data extracted were reviewed and double checked by two independent authors (B.F.S. and E.N.M.), who were blinded to the objectives of the meta-analysis.

A standard protocol for data extraction was used, including the following variables: number of participants, study design, duration of the study, interventions, demographic data, age and sex, chronic disease status, as well as exposures of interest before and after the interventions. Data was extracted to assess the effects of coconut oil on anthropometric profile (body weight, body mass index, waist circumference and body composition), lipid profile (LDL-C, HDL-C, total cholesterol [TC], TC/HDL ratio and triglycerides), glycemic profile (glucose, insulin, the homeostasis model assessment [HOMA] β and HOMA-S, HOMA-IR and glycated hemoglobin [HbA1c]), inflammatory profile (ultra-sensitive c-reactive protein [US-CRP], fibrinogen, total homocysteine [tcHcy], interleukins [IL] 1β, IL-6, IL-8 and interferon-gamma [IFN- γ]) and blood pressure (systolic blood pressure and diastolic blood pressure).

### Inclusion and exclusion criteria

Since our aim was to evaluate the isolated effect of coconut oil with no influence of dietary pattern, we considered eligible only RCTs (both parallel group or crossover randomized trials) which analyzed the effects of coconut oil intake in comparison to other fats, oils, or placebos on weight, lipid and glycemic profile, blood pressure, and subclinical inflammation of adults (≥ 18 years) published in all languages. We excluded non-randomized trials or studies with follow-ups shorter than seven days. Studies including patients with illnesses which affect metabolism, studies on animals or in vitro, and studies testing coconut products different from oils for intake were also excluded.

### Assessment of risk of bias and quality of evidence

Two pairs of authors independently assessed the risk of bias of each included trial using the revised Cochrane risk-of-bias tool for randomized trials (RoB 2) [31]. RoB 2 plots were generated using the Risk-of-bias VISualization (robvis) tool [32]. The overall certainty of evidence was assessed using the Grading of Recommendations Assessment, Development, and Evaluations (GRADE) [33].

### Statistical synthesis

Data were synthesized both qualitatively and quantitatively. To uniformly summarize the exposure data extracted, we standardized the units of concentration by applying standard conversion factors [34, 35]. Mean differences were calculated for continuous outcomes. For data collection, we prioritized intention-to-treat outcomes. Articles that expressed results as standard error had the results transformed into standard deviation. When a study did not express its results in a change from baseline manner, changes from baseline were calculated by subtracting final values from baseline values in each group and change from baseline standard deviations were imputed using a correlation coefficient calculated from the most similar study reported in considerable detail, in accordance with Cochrane Collaboration recommendations [36]. Where possible (that is, when parallel RCTs provided the baseline and final values of each outcome and when the crossover RCTs provided the order of different interventions and the measures of the variables of interest before and after each intervention), data were pooled using a meta-analytic approach. A random-effects model, with DerSimonian and Laird’s variance estimator, was used, and mean differences with 95% confidence intervals were calculated. A *p* value ≤ 0.05 was considered statistically significant. We used I^2^ statistics to assess the consistency of effects among studies [37]. We did not assess publication bias with a statistical test or funnel plot because such assessment is not recommended for sample sizes of less than 10 studies [38]. We used the statistical software R version 4.0.5 with the meta-version 4.18–1 package for meta-analysis.

We planned to perform subgroup analyses regarding the following factors: amount of coconut oil used, type of control group, sex, age, body mass index, geographical region where the study was conducted, studies in overweight/obesity subjects or in those with dyslipidemia, time of follow-up, and study sample size. When subgroup analysis of any forementioned factors was not possible due to the low number of studies – thus precluding our ability to quantitatively investigate the sources of heterogeneity –, this analysis was not performed and, therefore, is not mentioned in the results section of this text.

## Results

**Fig. 1:**
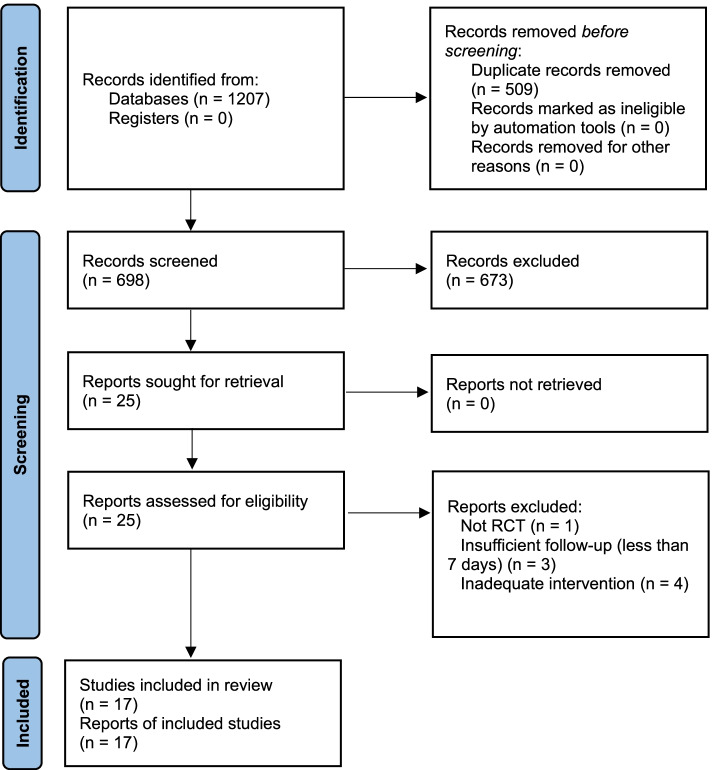
Flow diagram of study selection

After screening 1,160 potentially relevant studies, 17 fulfilled the selection criteria, of which seven studies were included in the meta-analysis. Inter-rater agreement assessed by the Kappa coefficient was 0.36 (% agreement: 91.1%) and –0.09 (% agreement: 84%) for the record screening and fulltext assessment stages, respectively. Details of the study selection are presented in Fig. 1 and Table S1.

The studies comprise 721 patients (age 18–68 years, 52% females) and follow-ups varied from one week to two years. The studies were performed in Europe (*n* = 2), Asia (*n* = 3), New Zealand (*n* = 1), the United States of America (*n* = 7), and Brazil (*n* = 4). In four studies, coconut oil was compared predominantly to MUFAs (olive and canola) [39–42], in 11 studies predominantly to PUFAs (soybean, chia, safflower, sunflower, and corn) [16, 17, 26, 42–49], and in six studies predominantly to SFAs (lard, butter, and palm oil) [39, 40, 44, 50, 51], followed by comparisons with soybean oil + psyllium, transgenic soybean, hydrogenated soybean, and a placebo in one study each [18, 26, 45, 50]. The amount of coconut oil consumed varied and is expressed differently among studies (Table 1): 12 to 30 ml of coconut oil/day (*n* = 5), as part of the amount of SFAs or total daily consumed fat (*n* = 1), a variation of 6 to 54.4 g/day (*n* = 5), or as part of the total caloric energy intake (15 to 21%) (*n* = 6). Seven studies included healthy individuals [18, 26, 39, 45, 48, 50, 51]; two included subjects with hypercholesterolemia [41, 44]; four, with abdominal obesity, overweight, or obesity [16, 42, 43, 49]; one, in postmenopausal women [46]; and one, individuals with CVD [17]. The key characteristics of all included studies are in Supplementary Tables S2, S3, S4, S5, S6 and summarized in Table 1.

**Table 1:** Characteristics of the included studies

Source	RCT design	Population	Intervention (daily amount of coconut oil)	Control	N In	N C	Female, N (%)	Baseline lipid profile, mean (SD)				Follow up (wk)
								**TC**	**HDL-C**	**LDL-C**	**TG**	
Assunção (2009) [43]	Parallel group	Women with abdominal obesity	30 ml	PUFA (soybean)	20	20	40 (100)	191 (32.7)	48.5 (8.7)	110.6 (28.7)	160 (81.9)	12
Cândido (2021) [42]	Parallel group	Women with IMC between 26 and 35 kg/m^2^, % body fat > 30%	25 ml	MUFA (olive oil) PUFA (soybean)	24	61	85 (100)	168.6 (9.7)	47.2 (2.7)	98.7 (9.7)	96.5 (8.9)	9
Chinwong (2017) [18]	Crossover	Healthy individuals	15 ml	Placebo (carboxymethycellulose solution)	34	34	16 (47)	190.8 (32.3)	60.6 (9.0)	116.5 (30.1)	68.5 (23.1)	8
Cox (1995) [44]	Crossover	Healthy individuals	39 g	SFA (butter) PUFA (safflower)	28	28	15 (53.6)	245.5 (27.5)	58 (15.5)	160.5 (29.4)	161.2 (79.7)	6
Ganji (1996) [45]	Crossover	Healthy individuals	20% of daily calories	PUFA (soybean and soybean + psyllium fiber)	10	10	5 (50)	187.9 (30.2)	56.5 (12)	107.5 (31.3)	132.9 (40.7)	4
Harris (2017) [46]	Crossover	Postmenopausal women	30 ml	PUFA (safflower)	14	14	14 (100)	223.1 (35.1)	64.1 (17.4)	128.7 (26.1)	105.2 (66.2)	4
Heber (1992) [50]	Crossover	Healthy men	17.5% of daily calories	SFA (palm) Hydrogenated soybean	13	13	0 (0)	176 (4)	37 (9)	120 (7)	95 (10)	3
Khaw (2018) [39]	Parallel group	Healthy individuals	50 g	SFA (butter) MUFA (olive)	30	66	63 (67)	229.31 (37.5)	73.47 (19.33)	138.05 (36.34)	NR	4
Lu (1997) [26]	Crossover	Healthy women	20% of daily calories	PUFA (soybean) A16 oil	15	15	15 (100)	162.4 (17.01)	52.97 (10.82)	90.1 (15.08)	93 (38.97)	3
McKenney (1995) [41]	Crossover	Individuals with hypercholesterolemia	Sufficient to increase in 10% the amount of daily calories from SFA	PUFA (canola)	11 (all)		5 (45.5)	222.3 (25.3)	49.8 (18.3)	149 (20.3)	117.1 (49.2)	6
Maki (2018) [47]	Crossover	Healthy individuals	54.4 g (muffins or rolls)	PUFA (corn)	13	12	13 (52)	188 (178, 215)^a^	46 (38.5, 55.5)^a^	123 (105, 142)^a^	92.5 (76.5, 136)^a^	4
Oliveira-de-Lira (2018) [16]	Parallel group	Obese women	6 g (capsules)	PUFA (safflower, chia, and soybean)	18	57	75 (100)	215.8 (24.2)	48.3 (8.1)	149.6 (23.7)	132.7 (41.7)	8
Reiser (1985) [48]	Crossover	Healthy women	21% of daily calories	SFA (lard) PUFA (safflower)	19 (all)		0 (0)	NA	NA	NA	NA	5
Schwab (1995) [51]	Crossover	Healthy women	16-26 g of coconut oil/day	SFA (palm)	7	8	15 (100)	187.9(25.5)	59.9 (10.4)	110.6 (17.8)	82.4 (34.5)	4
Vijayakumar (2016) [17]	Parallel group	Individuals with CVD	15% of daily calories	PUFA (sunflower)	99	99	13 (6.5)	148.3 (28.3)	40.8 (9.5)	88.2 (22.2)	113.1 (51.5)	2 years
Vogel (2020) [49]	Parallel group	Overweight men	12 ml	PUFA (soybean)	15	14	0 (0)	184.8 (44.1)	39.5 (10.1)	117 (36.1)	140.9 (67.1)	4
Voon (2011) [40]	Crossover	Normal and overweight healthy adults	20% of daily calories	SFA (palm) MUFA (olive)	15	30	36 (80)	182.1(25.5)	47.6 (10.8)	118.3 (22.4)	85 (39)	5

^a^ Median (IQR). Baseline lipid values are expressed in mg/dL. *Abbreviations*: *RCT*, Randomized clinical trial, *N In* Number of participants in the intervention arm, *N C* Number of participants in the control arm, *TC* Total cholesterol, *HDL-C* High-density lipoprotein cholesterol, *LDL-C* Low-density lipoprotein cholesterol, *TG* Triglycerides, *wk*, week, *PUFA* Polyunsaturated fatty acid, *SFA* Saturated fatty acid, *MUFA* Monounsaturated fatty acid, *A16* Transgenic soybean oil, *NA* data not avaliable, *CVD* Cardiovascular disease

We contacted authors from 12 trials, of whom three shared data with us (see Acknowledgments).

Trials reporting the effects of coconut oil on LDL-C to HDL-C ratio, TG, TC/HDL-C ratio*,* glycemic control (fasting glucose and HbA1c levels) and blood glucose regulation (insulin sensitivity and β-cell function), blood pressure, and subclinical inflammation profile are described in the Supplementary Appendix II. No differences in these parameters were found between coconut oil intake and the different control oils/fats. A summary of findings of the systematic review and meta-analysis is presented in Table 2.

**Table 2:** Summary of findings of the systematic review and meta-analysis

**Outcome group**		**Overall result, MD (95% CI)**	**Risk of Bias (RoB 2)**	**Certainty of evidence (GRADE)**
Anthropometric profile	Body weight	NS	High	Very low
	Waist circumference	NS		Very low
	Total body fat	NS		Very low
Lipid profile	LDL-C	NS	High	Very low
	HDL-C	+ 3.28 (0.66; 5.9)		Very low
	Triglycerides	NS		Very low
	TC/HDL-C	NS		Very low
Glycemic profile	Fasting blood glucose	NS	High	Very low
Inflammatory profile	US-CRP	NS	High	Very low

*Abbreviations*: *MD* Mean difference, *CI* Confidence interval, *GRADE* Grading of Recommendations Assessment, Development and Evaluations, *NS* Non-statistically significant, *LDL-C* Low-density lipoprotein cholesterol, *HDL-C* High-density lipoprotein cholesterol, *TC* Total cholesterol, *US-CRP* Ultra-sensitive c-reactive protein

### Coconut oil consumption and health outcomes

#### Anthropometric profile

##### Body weight

Nine studies analyzed the effects of coconut oil on body weight [16–18, 26, 39, 42, 43, 46, 51]. These studies included 533 participants (56.5% females, 18 to 68 years).

**Fig. 2:**
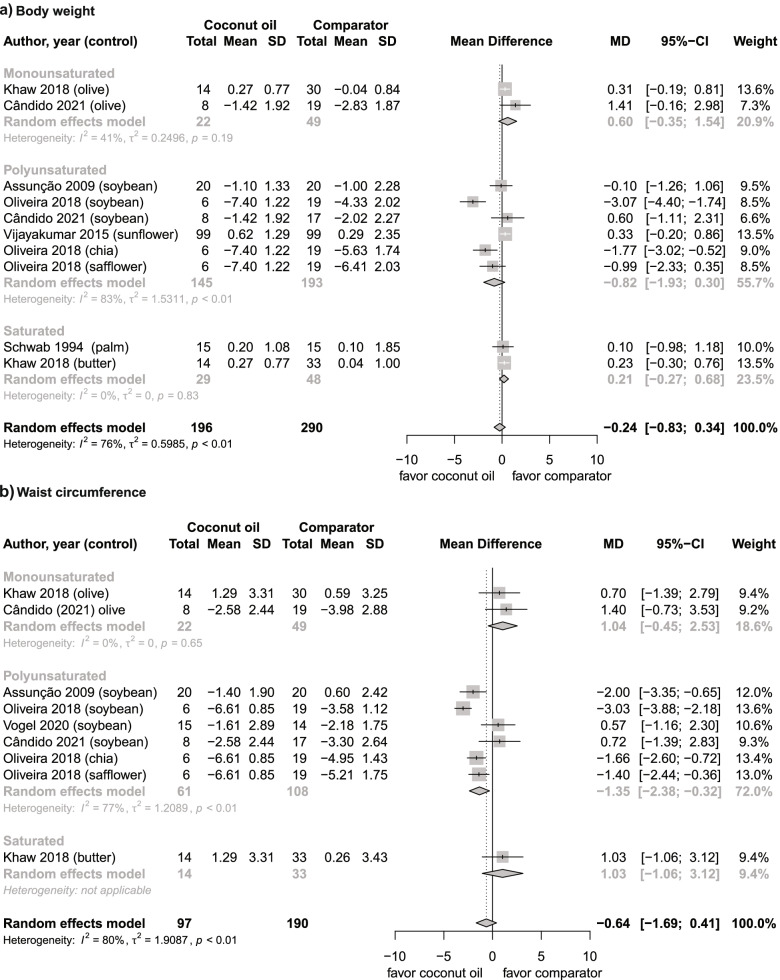
Forest plots of RCTs analyzing the effects of coconut oil intake on the anthropometric profile. **a** body weight, kg; **b** waist circumference, cm. Individual trial-specific estimates and their 95% CIs are indicated by the black dots and the horizontal line, respectively. The center of the diamonds indicates the pooled estimates and the width of the diamonds indicate the corresponding 95% CI

Six studies [16, 17, 39, 42, 43, 51] were included in the meta-analysis. Overall, weight loss was similar for those receiving coconut oil in comparison to those receiving other oils or fat (Fig. 2a).

The changes in body weight with coconut oil were also not significantly different in comparison to PUFA-rich oils, SFA-rich oils/fats, and MUFA-rich oils (Fig. 2a).

We also performed subgroup analyses considering the type of control group, gender, geographical region, studies in subjects with overweight/obesity, time of follow-up (< 1 year vs. ≥ 1 year), and the presence of co-interventions. These analyses did not explain the heterogeneity between groups and showed no changes in the direction of the results (Supplementary Figures S1, S2, S3, S4, S5, S6, S7, S8).

Additionally, two crossover studies found no differences between the consumption of coconut oil and other oils and fats on body weight [26, 46].

##### Waist circumference

Seven studies analyzed the effects of coconut oil on waist circumference [16, 39, 40, 42, 43, 46, 49]. These studies included 347 participants (80.1% females, 23 to 66 years).

Five studies were included in the meta-analysis [16, 39, 42, 43, 49]. Overall, the effect of coconut oil on waist circumference was not different in comparison to other interventions (Fig. 2b).

In order to understand the heterogeneity found, we performed a subgroup analysis. A small yet significant reduction in waist circumference is perceived while comparing the consumption of coconut oil with PUFA-rich oils,) but not with MUFA-rich oils (Supplementary Figure S9).

We also performed subgroup analyses considering the type of control group, gender, geographical region, studies in subjects with overweight/obesity, and the presence of co-interventions. These analyses did not explain the heterogeneity between groups and showed no changes in the direction of the results (Supplementary Figures S9, S10, S11, S12, S13, S14, S15).

In one crossover study, the consumption of coconut oil decreased waist circumference in comparison to safflower oil [40].

##### Body composition

Six studies analyzed the effect of coconut oil on body fat distribution [16, 17, 39, 40, 46, 49]. These studies included 460 participants (35.4% females, 29 to 68 years).

Five studies [16, 17, 39, 42, 49] were included in the meta-analysis. Overall, the effect of coconut oil intake on total body fat did not differ in comparison to other oils or fats (Supplementary Figure S16). Additionally, in comparison to PUFA- and MUFA-rich oils, the effect on total body fat was not different (Supplementary Figure S17).

Only one crossover study analyzed the effect of coconut oil on fat mass, including only postmenopausal women (*n* = 12, 100% females, 57.8 ± 3.7 years) [46]. The comparator was safflower, and there was no difference in body fat distribution between groups.

Two studies analyzed the effect of coconut oil on lean mass (*n* = 41, 29% females, 35–61 years) [46, 49]. The comparators were safflower and soybean oils, and, once again, coconut oil did not cause changes in lean mass in comparison with other oils (Supplementary Table S2).

#### Lipid profile

##### LDL-C

Seventeen studies analyzed the effects of coconut oil on LDL-C [16–18, 26, 39–51]. These studies included 515 participants (50% females, 18 to 68 years).

**Fig. 3:**
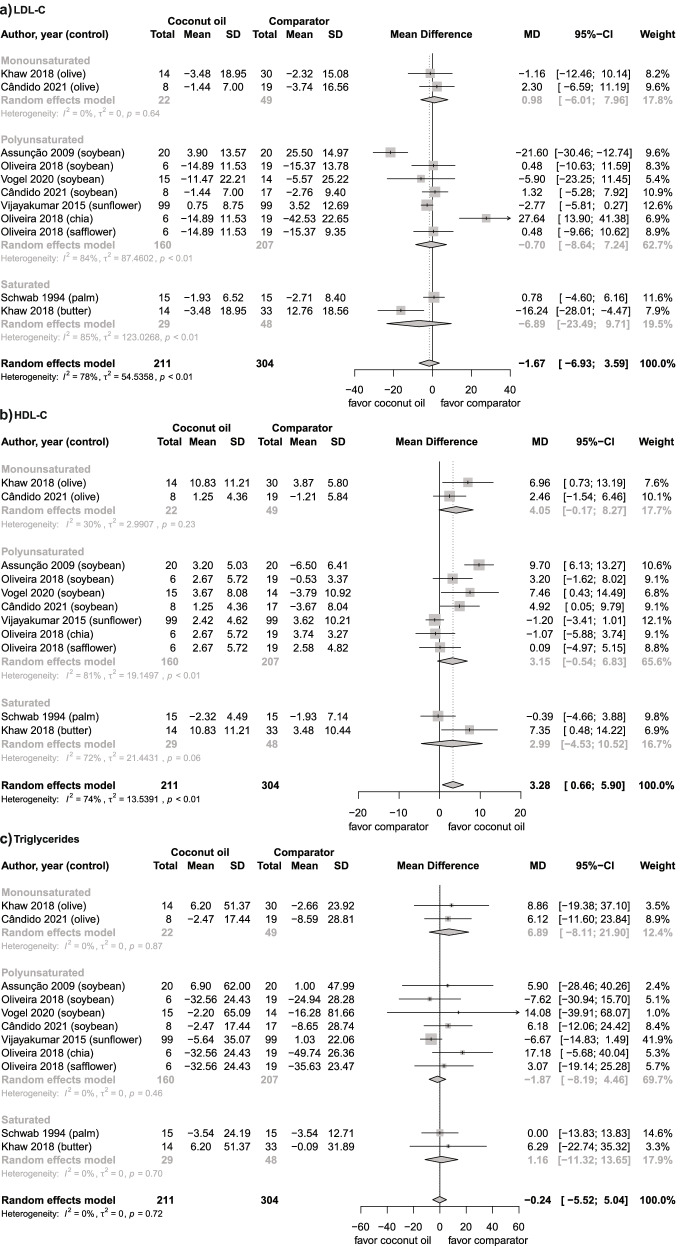
Forest plots of RCTs analyzing the effects of coconut oil intake on the lipid profile. **a** LDL-C, mg/dL; **b** HDL-C, mg/dL; **c** Triglycerides, mg/dL. Individual trial-specific estimates and their 95% CIs are indicated by the black dots and the horizontal line, respectively. The center of the diamonds indicates the pooled estimates and the width of the diamonds indicate the corresponding 95% CI

Seven studies [16, 17, 39, 42, 43, 49, 51] were included in the meta-analysis. Overall, the intake of coconut oil did not change LDL-C in comparison to other oils/fats (Fig. 3).

Coconut oil intake did not increase LDL-C as compared to PUFA-rich oils, SFA-rich oils/fats, and MUFA-rich oils (Fig. 3a).

We performed subgroup analyses considering the type of control group, gender, geographical region, studies in subjects with overweight/obesity, time of follow-up (< 1 year vs. ≥ 1 year), and the presence of co-interventions. These analyses did not explain the heterogeneity between groups and showed no changes in the direction of the results (Supplementary Figures S18, S19, S20, S21, S22, S23, S24, S25).

When analyzing the results of crossover studies, we observed that the intake of coconut oil increases LDL-C levels in comparison to butter, lard, and other oils [18, 26, 40, 41, 44–48, 50].

##### HDL-C

Seventeen studies analyzed the effects of coconut oil on HDL-C [16–18, 26, 39–51]. These studies included 515 participants (50% females, 18 to 68 years).

Seven studies [16, 17, 39, 42, 43, 49, 51] were included in the meta-analysis. Overall, the intake of coconut oil increased HDL-C by 3.28 mg/dL (Fig. 3).

We also performed subgroup analyses considering the type of control group, gender, geographical region, time of follow-up (< year vs. ≥ 1 year), and studies in overweight/obesity subjects (Figures S26, S27, S28, S29, S30, S31, S32). These analyses did not explain the heterogeneity between groups. However, when analyzed in different comparisons, the relative type of oil in the control group and studies only conducted in women, the significant increase in levels of HDL-C no longer existed. An additional subgroup analysis demonstrated that a significant increase in levels of HDL-C with coconut oil intake in comparison to other oils/fats was only identified in studies that included lifestyle interventions (Figure S33).

While analyzing the crossover studies [17, 25, 39, 40, 43–47, 49], we observed that the intake of coconut oil increases HDL-C in comparison to butter, lard, and other oils (data not shown).

##### Triglycerides

Seventeen studies analyzed the effects of coconut oil on TG levels [16–18, 26, 39–51]. These studies included 515 participants (50% females, 18 to 68 years).

Seven studies [16, 17, 39, 42, 43, 49, 51] were included in the meta-analysis. Overall, the intake of coconut oil did not change TG levels (Fig. 3).

The effect of coconut oil on TG was also not significant in comparison to MUFA-rich oils, PUFA-rich oils, and SFA-rich oils/fats (Fig. 3c).

We performed subgroup analyses considering the type of control group, gender, geographical region, studies in overweight/obesity subjects, time of follow-up (< 1 year vs. ≥ 1 year), and the presence of co-interventions. These analyses did not explain the heterogeneity between groups and showed no changes in the direction of the results (Supplementary Figures S34, S35, S36, S37, S38, S39, S40, S41).

Crossover studies [17, 25, 39, 40, 43–47, 49] showed that the intake of coconut oil increases TG levels in comparison to butter, lard, and other oils.

### Risk of bias and certainty of evidence

Detailed results of the assessment of risk of bias are summarized in Supplementary Figures S42, S43, S44, S45, S46. RCTs were overall rated either as having a high risk of bias or presenting some concerns in all analyzed outcomes. Risk of bias arose mainly from poor reporting of the randomization process and from deviations from intended interventions, in addition to carryover effects in crossover trials.

The certainty of evidence was rated as very low due to risk of bias and inconsistency in all analyzed outcomes, as follows (Supplementary Table S7).

## Discussion

This systematic review and meta-analysis of RCTs shows that, compared with the dietary consumption of other types of oils and fats, the intake of coconut oil is not superior in reducing body weight or abdominal circumference nor in changing body composition, LDL-C levels, TG, and TC/HDL-C ratio. Subgroup analyses comparing coconut oil with different types of oils based on their fatty acid composition have also confirmed our findings. However, increased levels of HDL-C were observed with the intake of coconut oil in comparison with that of other oils and fats.

Regarding the outcomes that were not included in meta-analyses, only two [17, 43] of the seven studies included in the systematic review that assessed glycemic control had the appropriate minimum follow-up time to analyze changes in HbA1c measures, given that the optimal timeframe to analyze alterations of HbA1c after dietary interventions is 12 weeks. Individual data from these studies do not suggest an impact of coconut oil intake on fasting glycemia, HbA1c, and estimates of β-cell function and insulin sensitivity, in line with findings from other previously published meta-analyses [29, 52].

Unlike other meta-analyses [28, 29, 52], we included studies that evaluated the effect of coconut oil on arterial blood pressure and we observed higher levels of systolic and diastolic blood pressure when coconut oil was compared with a placebo. When comparing coconut oil with olive oil and butter, only diastolic blood pressure levels increased [18, 39]. Despite scarce data addressing the effect of coconut oil on blood pressure, a cross-sectional study conducted in Southern India using a seven-day food survey found that the intake of coconut oil is associated with a higher risk of hypertension [53]. Although the mechanisms related to this finding remain unclear, foods rich in SFAs, such as coconut oil, can induce the development of central adiposity and insulin resistance, both phenomena related to the development of hypertension, which might explain these findings [11–14, 54].

Regarding markers of subclinical inflammation, as in other published meta-analyses [29, 52], we did not find a reduction in US-CRP with the intake of coconut oil in comparison to soybean oil, olive oil, and butter. Studies that evaluated the antioxidant potential effect of coconut oil are mostly performed in vitro, and their data should not be extrapolated to clinical practice [55]. Among the SFAs, lauric acid, which is roughly 50% of coconut oil composition, has the greatest inflammatory potential, resulting in an unfavorable rationale for conducting experimental studies evaluating the effect of the dietary consumption of coconut oil for this aim [56].

In line with previously published meta-analyses [28, 29, 52], we observed an increase in HDL-C levels with coconut oil in comparison with other oils and fats, which was also confirmed while comparing coconut oil intake with oils rich in MUFAs and PUFAs. These findings may be a result of its composition being predominantly made up of SFAs, resulting in a superior increase in HDL-C levels compared to oils/fats rich in MUFAs and PUFAs [29, 57]. However, neither Mendelian randomization analyses looking at genetic variants related to higher HDL levels [58], nor a meta-analysis of 108 RCTs evaluating the effects of different interventions that increase HDL-C levels [59] demonstrated that this increment protects against cardiovascular disease. In fact, dietary fat both increases transport rate and decreases the fractional catabolic rate of HDL cholesterol esther and apo A-I, intensifying the reverse cholesterol transport, only as an adaptation to the high load of a high fat diet [60, 61]. However, the consumption of SFA-rich oils, such as coconut oil, may not increase the apolipoprotein E-rich sub-fractions, which are mediators of cholesterol’s reverse transport, a main mechanism by which HDL-C exerts its cardio-protective effects [62]. Thus, it does not seem reasonable to advise the intake of coconut oil based on a possible protection against CVD derived from its effect on HDL-C.

An increase in plasmatic levels of HDL-C was observed with coconut oil intake compared with other oils while analyzing different studies (Fig. 3b), which could be explained by the fact that, in most of those studies, participants were exposed to co-interventions, including diet [16, 42, 43, 49, 51] and physical activity [16, 43] – which may have a significant impact on HDL-C levels [58, 59]. In fact, in one of these studies, participants significantly lost more weight, and, in two of them, there was a greater reduction in waist circumference with coconut oil compared to soybean oil. These results may have been driven by the real impact of coconut oil on HDL-C levels and may explain the heterogeneity that was found [16, 43].

In this review, changes in body weight were similar between coconut oil and other oils. In only one study, the group receiving coconut oil lost more body weight [16]. This result might be explained by the introduction of systematic error due to an imbalance of co-interventions, which might have been introduced as a result of lack of blinding of the staff who applied the lifestyle interventions. Similary, among the five studies which analyzed the impact of coconut oil in comparison to other oils/fats on central obesity, the two studies which demonstrated that the coconut oil group had a more significant reduction in waist circumference also applied lifestyle co-interventions in a similar manner, possibly resulting in the same forementioned systematic error [16, 43]. Subgroup analyses for studies regarding co-interventions have shown no differences in changes of body weight and waist circumference between coconut oil and diet interventions with other oils (Supplementary Figure S33 and S15).

Previous meta-analyses [29, 52] found higher LDL-C levels with the consumption of coconut oil in comparison with the intake of other oils and fats. These two reviews included crossover trials, and, in one of them [29], oils used in different arms causing very distinct responses in LDL-C levels were grouped as a single intervention against coconut oil [16]. We believe that this may explain the differences in findings between our meta-analysis and previously published ones. In line with our findings, Teng et al. (2020), in their analysis comparing coconut oil to other oils, did not find differences in levels of LDL-C, either [28]. Similar to what was found previously [29, 52], we also did not identify differences in changes of TG levels, TC/HDL-C ratio, LDL-C/HDL-C ratio, and body composition between the consumption of coconut oil and other oils/fats.

LDL-C concentration is one of the main targets for cardiovascular protection. However, some subtypes of LDL-C, especially the slow dense LDL-Cs, have been associated with a higher risk of atherogenesis [63]. Lipoprotein (a) (Lp[a]), a genetic variant of LDL, has also gained attention because of its considerable dyslipidemic potential [64]. There is still no clear evidence that reducing Lp(a) levels results in protection for cardiovascular outcomes [65], nor do we know how nonpharmacological treatments affect Lp(a) [66]. It seems that a healthy lifestyle can promote favorable changes in subclasses of lipoproteins [67], and that the characteristics of fatty acids could influence these changes [68].

None of the studies included in this systematic review assessed the subclasses of lipoproteins or Lp(a). However, a crossover trial including 31 women evaluated the effect of three different margarines, one of them containing 80% coconut oil, on plasma postprandial levels of some hemostatic variables and on fasting Lp(a). Data from only 11 subjects were evaluated, and there was a statistically significant reduction in Lp(a) in the margarine with coconut oil per se, and the total dietary composition (especially carbohydrates and total fat) was different between groups, which can influence the results [66]. New RCTs with higher methodological rigor are needed to confirm the potential of coconut oil in reducing Lp(a).

It is important to highlight that published meta-analyses about the topic included crossover studies with methodological limitations. In this meta-analysis, we only included crossover RCTs when it was possible to determine the order of the interventions and where the baselines and final averages of each arm were available. We then obtained the initial and final values of each outcome in each arm of the study before the participant was allocated to the other arm. This reduces the chance of the residual effects (carry-over) of the former intervention on the next one [36]. We contacted the authors of crossover studies and received these data from the authors of one study, which we included in our analysis [51]. In addition to that, we included two new RCTs [42, 49] that had not been included in the most recent meta-analysis [52].

This systematic review has some limitations. Generally, studies presented a small sample size with a short follow-up, which limits the analysis of the effects of a dietary intervention on cardiometabolic parameters. Therefore, the results must be interpreted with caution. Moreover, there was a limited number of studies analyzing the effect of the consumption of coconut oil on parameters other than lipid profile and body weight, such as body composition and glycemic and inflammatory profiles. The included studies also differ considerably from each other regarding population size and gender composition, time of follow-up, daily quantity of coconut oil consumed, type of coconut oil (virgin, extra virgin), product/vehicle for consumption (e.g.: as a capsule, as a supplement, heated as oil to cook with, or in preparations such as for muffins or crackers). Although this makes it difficult to compare different interventions, we were able to perform subgroup analyses comparing coconut oil with oils/fats with different fatty acid content in their compositions: SFA-, MUFA-, and PUFA-rich oils/fats. We also performed subgroup analyses according to the presence of other dietary interventions and/or physical activity, which may influence the effects attributed to coconut oil on the cardiometabolic parameters which were analyzed.

Up until now, the scientific community has lacked studies with a long-term follow-up and with a significant number of participants that evaluate the effect of coconut oil consumption on cardiovascular outcomes.

Conducting new RCTs examining cardiovascular safety comparing coconut oil with PUFA- and MUFA-rich oils evaluating traditional markers does not seem to be justifiable even though coconut oil is part of the diet in South Asian countries [20–22]. Moreover, in Western countries, stimulating the consumption of SFA-rich oils to the detriment of PUFA- and MUFA-rich oils may lead to an excessive intake of SFAs in populations that already have a diet rich in them [69].

## Conclusions

The dietary consumption of coconut oil instead of the consumption of PUFA- and MUFA-rich oils with well-established cardio-protective effects should not be encouraged in societies that are not used to consuming it. Moreover, educational strategies should be implemented to make populations, especially those used to consuming coconut oil, aware of the potential risks related with this intake. These populations should also be informed and encouraged to replace it with cardio-metabolically healthy options linked with a reduction in rates of CVD.

## Supplementary Information

**Additional file 1. **Supplementary material.

## Data Availability

The datasets used and/or analysed during the current study are available from the corresponding author on reasonable request.

## Abbreviations

CVD: Cardiovascular disease
GRADE: Grading of Recommendations, Assessment, Development and Evaluations
HDL-C: High-density lipoprotein cholesterol
HOMA: The homeostasis model assessment
HbA1c: Glycated hemoglobin
IL: Interleukins
IFN-g: Interferon-gamma
LDL-C: Low-density lipoprotein cholesterol
Lp(a): Lipoprotein [a]
MUFA: Monounsaturated fatty acid
PRISMA: Preferred Report Items for Systematic Reviews and Meta-Analysis
PUFA: Polyunsaturated fatty acid
RCTs: Randomized clinical trials
RoB: The risk of bias
Robvis: Risk-of-bias VISualization
SFA: Saturated fatty acid
TC: Total cholesterol
TcHc: Total homocysteine
TG: Triglycerides
US-CRP: Ultra-sensitive c-reactive protein
