# Supplementary material for: The effects of coconut oil on the cardiometabolic profile: a systematic review and meta-analysis of randomized clinical trials
Source: Lipids Health Dis. 2022 Aug 31;21:83. doi: 10.1186/s12944-022-01685-z (PMC9429773; doi:10.1186/s12944-022-01685-z)
Supplement: Supplementary file 1 — Additional file 1. Supplementary material. [file 12944_2022_1685_MOESM1_ESM.pdf]

SUPPLEMENTARY MATERIAL:

EFFECTS OF COCONUT OIL ON THE CARDIOMETABOLIC PROFILE: SYSTEMATIC REVIEW AND META-ANALYSIS OF RANDOMIZED CLINICAL TRIALS

Summary

Appendix I - Search strategy and search terms **4**
Full search strategy and search terms in Pubmed: 4
Full search strategy and search terms in Embase: 4
Full search strategy and search terms in LILACS: 4
Supplementary Tables **4**
Table S1. Detailed reasons for the exclusion of studies in the full text assessment of eligibility stage 4
Table S2. Summary of randomized clinical trials investigating the effect of coconut oil intake on anthropometric profile 5
Table S3. Summary of randomized clinical trials investigating the effect of coconut oil intake on glycemic profile 10
Table S4. Summary of randomized clinical trials investigating the effect of coconut oil on arterial blood pressure 14
Table S5. Summary of randomized clinical trials investigating the effect of coconut oil on the inflammatory profile 15
Table S6. Summary of randomized clinical trials investigating the effect of coconut oil on changes in the lipid profile 17
Table S7. Grading of Recommendations Assessment, Development and Evaluations (GRADE) - Coconut oil compared to other oils, fat or placebo for health outcomes 27
Appendix II - Additional results **28**
Lipid Profile 28
LDL-C to HDL-C ratio and TC: HDL-C ratio *28*
Appendix II - Figure 1. Forest plots of randomized controlled clinical trials investigating the effects of coconut oil intake on TC:HDL-C ratios 28
Appendix II - Figure 2. Forest plots of randomized controlled clinical trials investigating the effects of coconut oil intake vs MUFA and PUFA rich oils on TC:HDL-C ratios 29
Glycemic profile 29
Fasting blood glucose *29*
HbA1c *29*
Effects of coconut oil on insulin levels, β-cell function and indices of insulin sensitivity *30*
Appendix II - Figure 3. Forest plots of randomized controlled clinical trials investigating the effects of coconut oil intake on fasting blood glucose (mg/dL) 30
Appendix II - Figure 4. Forest plots of randomized controlled clinical trials investigating the effects of coconut oil intake vs PUFA and MUFA rich oils on fasting blood glucose (mg/dL). 31
Blood pressure 31
Systolic Blood Pressure *31*
Diastolic Blood Pressure *31*
Inflammatory profile 31
Appendix II - Figure 5. Forest plots of randomized controlled clinical trials investigating the effects of coconut oil intake on US-CRP (mg/dL) 32
Supplementary figures **33**
Figure S1. Forest plot of randomized controlled clinical trials investigating the effects in body weight (kg) of coconut oil intake versus PUFA and MUFA rich oils 33

Figure S2. Forest plot of randomized controlled clinical trials investigating the effect in body weight (kg) of coconut oil versus olive oil 33
Figure S3. Forest plot of randomized controlled clinical trials investigating investigating the effect in body weight (kg) of coconut oil versus soybean oil 34
Figure S4. Forest plot of randomized controlled clinical trials investigating the effect in body weight (kg) of coconut oil versus other oils in studies carried out in women 34
Figure S5. Forest plot of the randomized controlled clinical trials investigating the effect of coconut oil versus other oils in body weight (kg) of studies conducted in Brazil 34
Figure S6. Forest plot of randomized controlled clinical trials investigating the effect of coconut oil versus other oils/fats in body weight (kg) of studies carried out in patients with overweight/obesity 35
Figure S7. Forest plot of randomized controlled clinical trials investigating the effect on body weight (kg) of coconut oil versus other oils/fats without the long term study of Vijayakumar et al. 35
Figure S8. Forest plot of randomized controlled clinical trials investigating the effect in body weight (kg) of coconut oil versus other oils/fats in studies including co-intervention 36
Figure S9. Forest plot of randomized controlled clinical trials investigating the effects in waist circumference (cm) of coconut oil intake versus PUFA and MUFA rich oils 36
Figure S10. Forest plot of randomized controlled clinical trials investigating the effect in waist circumference (cm) of coconut oil versus olive oil 37
Figure S11. Forest plot of randomized controlled clinical trials investigating the effect in waist circumference (cm) of coconut oil versus soybean oil 37
Figure S12. Forest plot of randomized controlled clinical trials investigating the effect in waist circumference (cm) of coconut oil versus other oils when analyzing studies carried out in women 37
Figure S13. Forest plot of randomized controlled clinical trials investigating the effect in waist circumference (cm) of coconut oil versus other oils when analyzing studies conducted in Brazil 38
Figure S14. Forest plot of randomized controlled clinical trials investigating the effect in waist circumference (cm) of coconut oil versus other oils or/fat in patients with overweight/obesity 38
Figure S15 - Forest plot of randomized controlled clinical trials investigating the effect in waist circumference (cm) of coconut oil versus other oils/fats in studies including co-intervention 39
Figure S16. Forest plot of the randomized controlled clinical trials investigating the effects in % body fat of coconut oil intake in comparison to other oils/fat 39
Figure S17. Forest plot of randomized controlled clinical trials investigating the effect in % body fat of coconut oil intake vs PUFA and MUFA rich oils 40
Figure S18. Forest plot of randomized controlled clinical trials investigating the effects in LDL-C (mg/dL) of coconut oil intake vs PUFA and MUFA rich oils 40
Figure S19. Forest plot of randomized controlled clinical trials investigating the effect in LDL-C (mg/dL) of coconut oil versus olive oil 40
Figure S20. Forest plot of randomized controlled clinical trials investigating the effect in LDL-C (mg/dL) of coconut oil versus soybean oil 41
Figure S21. Forest plot of randomized controlled clinical trials investigating the effect in LDL-C (mg/dL) of coconut oil versus other oils when analyzing studies carried out in women 41
Figure S22. Forest plot of randomized controlled clinical trials investigating the effect in LDL-C (mg/dL) of coconut oil versus other oils when analyzing studies conducted in Brazil in LDL-C 41
Figure S23. Forest plot of randomized controlled clinical trials investigating the effect in LDL-C (mg/dL). of coconut oil versus other oils or/fat in patients with overweight/obesity 42
Figure S24. Forest plot of randomized controlled clinical trials investigating the effect in LDL-C (mg/dL) of coconut oil versus other oils or fat without a long term study (Vijayakumar et al) 42
Figure S25. Forest plot of randomized controlled clinical trials investigating the effect in LDL-C (mg/dL) of coconut oil versus other oils or fat with co-intervention 43
Figure S26. Forest plot of randomized controlled clinical trials investigating the effects in HDL-C (mg/dL) of coconut oil intake vs PUFA and MUFA rich oils 43
Figure S27. Forest plot of randomized controlled clinical trials investigating the effect in HDL-C (mg/dL) of coconut oil versus olive oil 43

Figure S28. Forest plot of randomized controlled clinical trials investigating the effect in HDL-C (mg/dL) of coconut oil versus soybean oil 44
Figure S29. Forest plot of randomized controlled clinical trials investigating the effect in HDL-C (mg/dL) of coconut oil versus other oils when analyzing studies carried out in women 44
Figure S30. Forest plot of randomized controlled clinical trials investigating the effect in HDL-C (mg/dL) of coconut oil versus other oils when analyzing studies conducted in Brazil 44
Figure S31. Forest plot of randomized controlled clinical trials investigating the effect in HDL-C (mg/dL) of coconut oil versus other oils or fat in patients with overweight/obesity 45
Figure S32. Forest plot of randomized controlled clinical trials investigating the effect in HDL-C (mg/dL) of coconut oil versus other oils or fat without a long term study (Vijayakumar et al) 45
Figure S33. Forest plot of randomized controlled clinical trials investigating the effect in HDL-C (mg/dL) of coconut oil versus other oils or fat with co-intervention 46
Figure S34. Forest plot of randomized controlled clinical trials investigating the effects in TG (mg/dL) of coconut oil intake vs PUFA and MUFA rich oils 46
Figure S35. Forest plot of randomized controlled clinical trials investigating the effect in TG (mg/dL) of coconut oil versus olive oil 47
Figure S36. Forest plot of randomized controlled clinical trials investigating the effect in TG (mg/dL) of coconut oil versus soybean oil 47
Figure S37. Forest plot of randomized controlled clinical trials investigating the effect in TG (mg/dL) of coconut oil versus other oils when analyzing studies carried out in women 47
Figure S38. Forest plot of randomized controlled clinical trials investigating the effect in TG (mg/dL) of coconut oil versus other oils when analyzing studies conducted in Brazil 48
Figure S39. Forest plot of randomized controlled clinical trials investigating the effect in TG (mg/dL) of coconut oil versus other oils or fat in patients with overweight/obesity 48
Figure S40. Forest plot of randomized controlled clinical trials investigating the effect in TG (mg/dL) of coconut oil versus other oils or fat without a long term study (Vijayakumar et al) 48
Figure S41. Forest plot of randomized controlled clinical trials investigating the effect in TG (mg/dL) of coconut oil versus other oils or fat with co-intervention 49
Figure S42: RoB 2.0 risk of bias in RCTs assessing the effects of coconut oil in the lipid profile 50
Figure S43: RoB 2.0 risk of bias in RCTs assessing the effects of coconut oil in the anthropometric profile 51
Figure S44: RoB 2.0 risk of bias in RCTs assessing the effects of coconut oil in the glycemic profile 52
Figure S45: RoB 2.0 risk of bias in RCTs assessing the effects of coconut oil in blood pressure 53
Figure S46: RoB 2.0 risk of bias in RCTs assessing the effects of coconut oil in the inflammatory profile 53
PRISMA 2020 Checklist 54
References 57

Appendix I - Search strategy and search terms

Full search strategy and search terms in Pubmed:

(((("coconut oil" [Supplementary Concept]) OR "coconut oil") OR coconut)) AND ((randomized controlled trial[pt] OR controlled clinical trial[pt] OR randomized controlled trials[mh] OR random allocation[mh] OR double-blind method[mh] OR single-blind method[mh] OR clinical trial[pt] OR clinical trials[mh] OR ("clinical trial"[tw]) OR ((singl*[tw] OR doubl*[tw] OR trebl*[tw] OR tripl*[tw]) AND (mask*[tw] OR blind*[tw])) OR ("latin square"[tw]) OR placebos[mh] OR placebo*[tw] OR random*[tw] OR research design[mh:noexp] OR follow-up studies[mh] OR prospective studies[mh] OR cross-over studies[mh] OR control*[tw] OR prospective*[tw] OR volunteer*[tw] NOT (animal[mh] NOT human[mh]))

Full search strategy and search terms in Embase:

('adult'/exp OR 'adult' OR 'adults' OR 'grown-ups' OR 'grownup' OR 'grownups') AND ('coconut oil'/exp OR 'coconut butter' OR 'coconut fat' OR 'coconut oil' OR 'coconut oil emulsion' OR 'copra oil' OR 'oil, coconut') AND ('randomized controlled trial'/exp OR 'controlled trial, randomized' OR 'pragmatic clinical trial' OR 'pragmatic clinical trials' OR 'randomised controlled study' OR 'randomised controlled trial' OR 'randomized controlled study' OR 'randomized controlled trial' OR 'trial, randomized controlled')

Full search strategy and search terms in LILACS:

(tw:(óleo de coco)) AND (tw:(ensaio clínico))

Supplementary Tables

Table S1. Detailed reasons for the exclusion of studies in the full text assessment of eligibility stage

**Record**	**Reason for exclusion**
Francisco A O Júnior, et al., Coconut Oil Supplementation Does Not Affect Blood Pressure Variability and Oxidative Stress: A Placebo-Controlled Clinical Study in Stage-1 Hypertensive Patients. Nutrients, 2021; 28;13(3):798. doi: 10.3390/nu13030798.	Combination of interventions in groups
Mendis, S., et al. The effect of daily consumption of coconut fat and soya-bean fat on plasma lipids and lipoproteins of young normolipidemic men. Br J Nutr, 1990;63(3):547-52. doi: 10.1079/bjn19900141	Non-randomized clinical trial
Muller, H, et al. The serum LDL/HDL cholesterol ratio is influenced more favorably by exchanging saturated with unsaturated fat than by reducing saturated fat in the diet of women. J Nutr, 2003;133(1):78-83. doi: 10.1093/jn/133.1.78.	Mixing more than one oil in the same food (eg margarine, coconut oil, soy oil), which does not allow us to know the real effects of coconut oil on the outcomes studied.
Ng, T K. et al. Nonhypercholesterolaemic effects of a palm-oil diet in Malaysian volunteers. Am J Clin Nutr, 1991; 53(4 Suppl):1015S-1020S. doi: 10.1093/ajcn/53.4.1015S.	Inadequate intervention

Panth, N., et al. Medium-chain fatty acids lower postprandial lipemia: A randomized crossover trial. Clin Nutr, 2020; 39(1):90-96. doi: 10.1016/j.clnu.2019.02.008.	Insufficient follow-up (<7 days)
Sciarrilo, C M., et al. **Postprandial Lipemic Responses to Various Sources of Saturated and Monounsaturated Fat in Adults. Nutrients, 2019;** May; 11(5): 1089. doi: 10.3390/nu11051089.	Insufficient follow-up (<7 days)
Trepanowski J F., et al. A 21-day Daniel fast with or without krill oil supplementation improves anthropometric parameters and the cardiometabolic profile in men and women. Nutr Metab (Lond), 2012; 13;9(1):82. doi: 10.1186/1743-7075-9-82.	Data from the placebo and intervention groups were pooled, not being able to analyze the real effects of coconut oil on the outcomes of interest.
Valente FX., et al. Effects of coconut oil consumption on energy metabolism, cardiometabolic risk markers, and appetitive responses in women with excess body fat. Eur J Nutr. 2018; 57(4):1627-1637. doi: 10.1007/s00394-017-1448-5.	Insufficient follow-up (<7 days)

Table S2. Summary of randomized clinical trials investigating the effect of coconut oil intake on anthropometric profile

**Author and Year**	**Study design (Country)**	**Follow-up**	**Sample**	**Intervention**	**Comparator**	**Last measurements of anthropometric profile**
Assunção (2009)	Randomized clinical trial (Brazil)	12 weeks	n = 40 women with abdominal obesity<br>Age = 29.8 ± 6.6 years	30 ml of coconut oil should be added to the three main meals of the day, in the	30 ml of soybean oil should be added to the three main meals of the day, in the	Body weight (kg): soybean oil (75 ± 9.1) > coconut oil (72.1 ± 9.1)*<br>BMI (kg/m²): soybean oil (30.7 ± 3.3) > coconut oil (30.5 ± 3.6)*

			BMI = 31.1 ± 3.4 kg/m²	common preparation of meals	common preparation of meals	Waist circumference (cm): coconut oil = soybean oil (97 ± 7)
Cândido (2021)	Randomized clinical trial (Brazil)	9 weeks	n = 52 women with BMI between 26 e 35 kg/m², %G > 30%<br>Age = 26.81 ± 0.74	Vitamin breakfast prepared with 25 ml of coconut oil, skimmed milk powder and some fruit flavoring, chocolate or cappuccino	Vitamin breakfast prepared with 25 ml of soybean oil, skimmed milk powder and some fruit flavoring, chocolate or cappuccino<br><br>Vitamin breakfast prepared with 25 ml of olive oil, skimmed milk powder and some fruit flavoring, chocolate or cappuccino	Body weight (kg): soybean oil (77.24 ± 2.08) > coconut oil (75.99 ± 2.92) > olive oil (75.81 ± 1.65)<br>Waist circumference (cm): coconut oil (94.17 ± 2.24) > olive oil (93.58 ± 1.91) > soybean oil (92.93 ± 1.87)<br>Total fat (%): soybean oil (46.54 ± 0.90) > coconut oil (45.67 ± 1.29) > olive oil (45.27 ± 1.07)
Chinwong (2017)	Randomized<br>crossover trial, open-label<br>(Thailand)	8 weeks	n = 32 healthy individuals<br>Age = 21 ± 0.7 years<br>BMI = 20.8 ± 3.4 kg/m²	30 ml/day of coconut oil extra virgin	30 ml/day of 2% carboxymethylcellulose solution (CMC) solution	Body weight (kg): coconut oil (59.20 ± 12.57) > CMC solution (58.73 ± 12.02)<br>BMI (kg/m²): coconut oil (20.88 ± 3.55) > CMC solution (20.71 ± 3.33)
Harris (2017)	Randomized<br>crossover trial	4 weeks	n = 12 postmenopausal women	Ingestion of 30 ml of coconut oil per day in ready-made preparations (smoothies-like beverages or in the	Ingestion of 30ml of safflower oil per day in ready-made preparations (smoothies-like beverages	Body Weight (kg): coconut oil = safflower oil (68.9 ± 11.5)

	(EUA)		Age = 57.8 ± 3.7 years<br>BMI = 26.4 ± 4.4 kg/m²	preparation of salad dressings).	or in the preparation of salad dressings).	Waist circumference (cm): safflower oil (87.1 ± 11.9) > coconut oil (85.5 ± 11)<br>Total fat (%): coconut oil = safflower oil (37.5 ± 6)<br>Fat mass (kg): coconut oil = safflower oil (25.7 ± 8)<br>Lean mass (kg): coconut oil = safflower oil (41.5 ± 4.5)
Khaw<br>(2018)	Randomized clinical trial<br>(UK)	4 weeks	n = 94 healthy individuals<br>Age = 59.9 ± 6.1 years<br>BMI = 25.1 ± 4.2 kg/m²	Coconut oil: 50g of coconut oil incorporated in the usual daily diet in substitution of other fats or ingested as a supplement.	Butter: 50 g of butter incorporated in the usual daily diet in substitution of other fats or ingested as a supplement.<br>Olive oil: 50 g of olive oil incorporated in the usual daily diet in substitution of other fats or ingested as a supplement.	Body weight (kg): coconut oil (74 ± 15.6) > butter (70.9 ± 11.8) > olive oil (70.4 ± 14.0)<br>BMI (kg/m²): coconut oil (25.6 ± 4.6) > olive oil (24.9 ± 4.5) > butter (24.8 ± 3.6)<br>Waist circumference (cm): coconut oil (86.6 ± 13.6) > olive oil (86.3 ± 12.1) > butter (84.0 ± 8.6)<br>Body fat (%): olive oil (30.9 ± 9.5) > coconut oil (29.6 ± 10.3) > butter (29.6 ± 8.7)
Lu<br>(1997)	Randomized<br>crossover trial	3 weeks	n = 15 healthy women<br>Age = 20.0 ± 2.0 years	Coconut oil: 10% of daily VCT from coconut oil	A16 oil: 10% of daily VCT from oil A16 (transgenic soybean oil, composed of	Body Weight (kg): coconut oil = A16 oil = soybean oil (63.30 ± 7.00) (N/S)

	(EUA)		BMI = 22.6 ± 2.4 kg/m²		a lower ratio of 18: 3 without trans fats)<br><br>Soybean oil: 10% of daily VCT from soybean oil	BMI (kg/m²): coconut oil = A16 oil = soybean oil (22.80 ± 2.50)
Oliveira-de-Lira (2018)	Randomized Clinical Trial<br>(Brazil)	8 weeks	n = 75 obese women<br>Age = 34.07 ± 5.4 years	Coconut oil: 6 ml/day supplemented in capsules 30 min before main meals.	Safflower oil: 6 ml/day supplemented in capsules 30 min before main meals.<br><br>Chia oil: 6 ml/day supplemented in capsules 30 min before main meals<br><br>Soybean oil: 6 ml/day supplemented in capsules 30 min before main meals.	Body Weight (kg): soybean oil (82.98 ± 8.09) > safflower oil (82.72 ± 7.67) > chia oil (80.6 ± 6.79) > coconut oil (79.57 ± 8.12)*<br><br>BMI (kg/m²): soybean oil (32.66 ± 2.86) > safflower oil (32.33 ± 2.44) > chia oil (31.26 ± 1.96) > coconut oil (30.76 ± 2.33)*<br><br>Waist circumference (cm): soybean oil (94.79 ± 2.66) > chia oil (94.68 ± 4.93) > safflower (94.32 ± 6.25) > coconut oil (91.89 ± 6.05)*<br><br>Body fat (%): chia oil (40.84 ± 3.33) > soybean oil (39.73 ± 3.37) > sunflower oil (39.62 ± 4.53) > coconut oil (37.57 > 4.03)*<br><br>Lean mass (kg): coconut oil (62.32 ± 4.49) > safflower oil (60.38 ± 4.53) > soybean oil

						(60.27 ± 3.37) > chia oil (59.16 ± 3.33)*
Schwab<br>(1994)	Randomized clinical trial (Finland)	4 weeks	n = 15 healthy women<br>Age = 23.9 ± 4.6 years<br>BMI = 21.4 ± 1.9 kg/m²	Refined coconut oil (16 to 26 g/day of coconut oil = 4% of the daily VCT). This diet also contained oils from other sources: rapeseed oil (5 to 8g / day), olive oil (3 to 4.5g / day) and sunflower oil (2 to 3.5 g/day).	Refined palm oil, bleached and deodorized (22 to 33 g/day of palm oil = 4% of daily VCT). This diet also contained soybean oil (2 to 5 g/day) as a source of fat.	Body Weight (kg): coconut oil = palm oil (58.9 ± 7.35)
Vijayakumar<br>(2015)	Randomized<br>clinical trial<br>(India)	2 years	n = 198 individuals with CVD<br>Age = 59.0 ± 8.7 years<br>BMI = 24.7 ± 4.7 kg/m²	15% of the daily VCT of a trademark coconut oil to be used as cooking oil.	15% of the daily VCT of a trademark sunflower oil to be used as cooking oil.	Body weight (kg): sunflower oil (64.8 ± 9.0) > coconut oil (64.23 ± 8.78)<br>BMI (kg/m²): coconut oil (24.72 ± 3.07) > sunflower oil (24.54 ± 3.07)<br>Body Fat (%): coconut oil (17.48 ± 2.91) > sunflower oil (17.39 ± 3.62)<br>Waist hip ratio: coconut oil (0.97 ± 0.05) > sunflower oil (0.96 ± 0.05)
Vogel<br>(2020)	Randomized clinical trial<br>(Brazil)	45 days	n = 29 men with obesity I<br>Age = between 20–59 years	Addition of 1 tablespoon (12ml) of coconut oil<br>to dinner	Addition of 1 tablespoon (12ml) of soybean oil<br>to dinner	BMI (kg/m²): coconut oil (32.28 ± 1.83) > soybean oil (31.17 ± 1.65)

						Waist circumference (cm): coconut oil (107.13 ± 4.38) > soybean oil (106.17 ± 4.60)<br>Body fat (%): coconut oil (25.94 ± 3.64) > soybean oil (24.06 ± 5.01)<br>Lean mass (kg): soybean oil (74.06 ± 3.64) > coconut oil (72.58 ± 3.46)<br>Waist hip ratio: soybean oil (0.96 ± 0.05) > coconut oil (0.94 ± 0.05)

* Significantly different (P<0.05). BMI: body mass index; VCT: total caloric value; CVD: cardiovascular disease.

Table S3. Summary of randomized clinical trials investigating the effect of coconut oil intake on glycemic profile

**Author and Year**	**Study design (Country)**	**Follow-up**	**Sample**	**Intervention**	**Comparator**	**Last measurements of glycemic profile (mg/dL), except where specified)**
Assunção (2009)	Randomized clinical trial (Brazil)	12 weeks	n = 40 women with abdominal obesity<br>Age = 29.8 ± 6.6 years<br>BMI = 31.1 ± 3.4 kg/m²	30 ml of coconut oil should be added to the three main meals of the day, in the common preparation of meals	30 ml of soybean oil should be added to the three main meals of the day, in the common preparation of meals	Glucose: coconut oil (82.8 ± 5.4) > soybean oil (78.5 ± 9.9)<br>Insulin (mIu/DL): coconut oil (9.8 ± 4.1) > soybean oil (7.6 ± 2.1)<br>HOMA-β: coconut oil (39.4 ± 18) > soybean oil (31.8 ± 9.8)

						HOMA-S: coconut oil (2 ± 0.9) > soybean oil (1.48 ± 0.45)*
Cândido (2021)	Randomized clinical trial (Brazil)	9 weeks	n = 52 women with BMI between 26 e 35 kg/m², %G > 30%<br>Age = 26.81 ± 0.74	Vitamin breakfast prepared with 25 ml of coconut oil, skimmed milk powder and some fruit flavoring, chocolate or cappuccino	Vitamin breakfast prepared with 25 ml of soybean oil, skimmed milk powder and some fruit flavoring, chocolate or cappuccino<br>Vitamin breakfast prepared with 25 ml of olive oil, skimmed milk powder and some fruit flavoring, chocolate or cappuccino	Glucose: coconut oil (85.69 ± 2.11) > olive oil (84.28 ± 1.19) > soybean oil (82.65 ± 0.01)*<br>Insulin (mIu/DL): soybean oil (9.19 ± 1.12) > coconut oil (8.03 ± 0.95) > olive oil (7.99 ± 0.76)
Heber (1992)	Randomized crossover trial (USA)	3 weeks	n = 9 healthy men	35% of the calories of the day were derived from LIP and of these, 50% were from coconut oil, which was incorporated into muffins or biscuits. Each muffin or cookie provided 13.7 g of LIP of the oil test.	35% of the calories of the day were derived from LIP and of these, 50% were from palm oil or hydrogenated soybean oil which was incorporated into muffins or biscuits. Each muffin or cookie provided 13.7 g of LIP of the oil test.	Glucose: hydrogenated soybean oil (81.0 ± 6.0) > coconut oil (78.0 ± 2.0) > palm oil (69.0 ± 7.0)<br>Insulin (mIu/Dl): coconut oil (14.0 ± 2.0) > hydrogenated soybean oil (12.0 ± 4.0) > palm oil (11.0 ± 1.0)
Khaw (2018)	Randomized clinical trial (UK)	4 weeks	n = 94 healthy individuals<br>Age = 59.9 ± 6.1 years<br>BMI = 25.1 ± 4.2 kg/m²	Coconut oil: 50 g of coconut oil incorporated in the usual daily diet in substitution of other fats or ingested as a supplement.	Butter: 50 g of butter incorporated in the usual daily diet in substitution of	Glucose: butter (97.2 ± 10.8) > olive oil (95.4 ± 10.8) > coconut oil (95.4 ± 9)

					other fats or ingested as a supplement.<br><br>Olive oil: 50 g of olive oil incorporated in the usual daily diet in substitution of other fats or ingested as a supplement.	
Oliveira-de-Lira (2018)	Randomized Clinical Trial<br>(Brazil)	8 weeks	n = 75 obese women<br>Age = 34.07 ± 5.4 years	Coconut oil: 6 ml/day supplemented in capsules 30 min before main meals.	Safflower oil: 6 ml/day supplemented in capsules 30 min before main meals.<br><br>Chia oil: 6 ml/day supplemented in capsules 30 min before main meals.<br><br>Soybean oil: 6 ml/day supplemented in capsules 30 min before main meals.	A1c (%): chia oil (4.95 ± 0.24) > safflower oil (4.91 ± 0.30) > soybean oil (4.89 ± 0.29) > coconut oil (4.58 ± 0.21)*
Vijayakumar (2015)	Randomized clinical trial<br>(India)	2 years	n = 198 individuals with CVD<br>Age = 59.0 ± 8.7 years<br>BMI = 24.7 ± 4.7 kg/m²	15% of the daily VCT of a trademark coconut oil to be used as cooking oil.	15% of the daily VCT of a trademark sunflower oil to be used as cooking oil.	A1c (%): sunflower oil (6.77 ± 1.28) > coconut oil (6.54 ± 1.32)

Vogel<br>(2020)	Randomized clinical trial<br>(Brazil)	45 days	n = 29 men with obesity I<br>Age = between 20–59 years	Addition of 1 tablespoon (12ml) of coconut oil to dinner	Addition of 1 tablespoon (12ml) of soybean oil<br>to dinner	Glucose: soybean oil (85.43 ± 5.93) > coconut oil (78.73 ± 10.97)<br>Insulin (mIu/Dl): soybean oil (9.85 ± 9.93) > coconut oil (5.13 ± 3.79)<br>HOMA-IR: soybean oil (2.16 ± 2.17) > coconut oil (0.92 ± 0.63)

* Significantly different (P<0.05). BMI: body mass index; LIP: lipids; CVD: cardiovascular disease; VCT: total caloric value

Table S4. Summary of randomized clinical trials investigating the effect of coconut oil on arterial blood pressure

**Author and Year**	**Study design (Country)**	**Follow-up**	**Sample**	**Intervention**	**Comparator**	**Last measurements of blood pressure ( ) or changes [ ] (mm Hg)**
Chinwong (2017)	Randomized crossover trial, open-label (Thailand)	8 weeks	n = 32 healthy individuals<br>Age = 21 ± 0.7 years<br>BMI = 20.8 ± 3.4 kg/m²	30 ml/day of extra virgin coconut oil	30 ml/day of 2% carboxymethylcellulose solution (CMC) solution	SBP: CMC solution (117.63 ± 13.49) > coconut oil (114.84 ± 11.29)<br>DBP: coconut oil (70.41 ± 6.42) > CMC solution (69.50 ± 13.28)
Khaw (2018)	Randomized clinical trial (UK)	4 weeks	n = 94 healthy individuals<br>Age = 59.9 ± 6.1 years<br>BMI = 25.1 ± 4.2 kg/m²	Coconut oil: 50 g of coconut oil incorporated in the usual daily diet in substitution of other fats or ingested as a supplement.	Butter: 50 g of butter incorporated in the usual daily diet in substitution of other fats or ingested as a supplement.<br><br>Olive oil: 50 g of olive oil incorporated in the usual daily diet in substitution of other fats or ingested as a supplement.	SBP: coconut oil [0.18 ± 11.46] > butter [-3.79 ± 11.11] > olive oil [- 3.67 ± 8.23]<br>DBP: coconut oil [-2.02 ± 5.71] > butter [-1.33 ± 6.24] > olive oil [-0.45 ± 8.48]

* Significantly different (P<0.05). BMI: body mass index; SBP: systolic blood pressure; DPB: diastolic blood pressure

Table S5. Summary of randomized clinical trials investigating the effect of coconut oil on the inflammatory profile

**Author and Year**	**Study design (Country)**	**Follow-up**	**Sample**	**Intervention**	**Comparator**	**Last measurements of inflammatory profile**
Assunção (2009)	Randomized clinical trial (Brazil)	12 weeks	n = 40 women with abdominal obesity<br>Age = 29.8 ± 6.6 years<br>BMI = 31.1 ± 3.4 kg/m²	30 ml of coconut oil should be added to the three main meals of the day, in the common preparation of meals	30 ml of soybean oil should be added to the three main meals of the day, in the common preparation of meals	US-CRP (mg/dL): soybean oil (4.2 ± 3.2) > coconut oil (3.7 ± 1.7)<br>Fibrinogen (mg/dL): coconut oil (243.8 ± 41.9) > soybean oil (243.6 ± 43.9)
Khaw (2018)	Randomized clinical trial (UK)	4 weeks	n = 94 healthy individuals<br>Age = 59.9 ± 6.1 years<br>BMI = 25.1 ± 4.2 kg/m²	Coconut oil: 50 g of coconut oil incorporated in the usual daily diet in substitution of other fats or ingested as a supplement.	Butter: 50 g of butter incorporated in the usual daily diet in substitution of other fats or ingested as a supplement.<br>Olive oil: 50 g of olive oil incorporated in the usual daily diet in substitution of other fats or ingested as a supplement.	US-CRP (mg/dL): olive oil (0.19 ± 0.2) > butter (0.16 ± 0.11) > coconut oil (0.14 ± 0.13)

Vijayakumar (2015)	Randomized crossover trial (India)	2 years	n = 198 individuals with CVD<br>Age = 59.0 ± 8.7 years<br>BMI = 24.7 ± 4.7 kg/m²	15% of the daily VCT of a trademark coconut oil to be used as cooking oil.	15% of the daily VCT of a trademark sunflower oil to be used as cooking oil.	US-CRP (IU/L): sunflower oil (1.43 ± 1.72) > coconut oil (1.23 ± 1.59)
Voon (2011)	Randomized crossover trial (Malaysia)	5 weeks	n = 45 normal and overweight healthy adults<br>Age: 30.1 ± 8.3 years<br>BMI = 23.1 ± 3.7 kg/m²	Meals with 30% energy from fat, two-thirds of which was from coconut oil (20% total energy)	Meals with 30% energy from fat, two-thirds of which was from palm oil or extra virgin olive oil (20% total energy)	tcHcy (µmol/L): coconut oil (9.13 ± 3.17) > palm oil (8.88 ± 3.05) > olive oil (8.76 ± 2.96)<br>US-CRP (mg/dL): olive oil (2.19 ± 2.36) > palm oil (2.15 ± 2.89) > coconut oil (1.96 ± 2.01)<br>IL-1β (pg/mL): coconut oil (25.93 ± 71.05) > olive oil (23.63 ± 57.95) > palm oil (23.09 ± 57.93)<br>IL-6 (pg/mL): coconut oil (9.91 ± 44.07) > olive oil (8.71 ± 31.15) > palm oil (8.52 ± 32.19)<br>IFN- γ (pg/mL): palm oil (17.04 ± 37.78) > olive oil (16.2 ± 36.68) > coconut oil (11.53 ± 30.78)<br>IL-8 (pg/mL): olive oil (71.02 ± 130.1) > palm oil (67.15 ±

						108.46) > coconut oil (47.35 ± 85.3)

* Significantly different (P<0.05). BMI: body mass index; LIP: lipids; CVD: cardiovascular disease; VCT: total caloric value

Table S6. Summary of randomized clinical trials investigating the effect of coconut oil on changes in the lipid profile

**Author and Year**	**Study design (Country)**	**Follow-up**	**Sample**	**Intervention**	**Comparator**	**Last measurements of lipids ( ) or changes in lipids [ ] (mg/dL, except where specified)**
Assunção (2009)	Randomized clinical trial (Brazil)	12 weeks	n = 40 women with abdominal obesity<br>Age = 29.8 ± 6.6 years<br>BMI = 31.1 ± 3.4 kg/m²	30 ml of coconut oil should be added to the three main meals of the day, in the common preparation of meals	30 ml of soybean oil should be added to the three main meals of the day, in the common preparation of meals	TC: soybean oil (209.3 ± 28.5) > coconut oil (198.1 ± 39.0) *<br>LDL-C: soybean oil (134.1 ± 28.7) > coconut oil (116.5 ± 36.8) *<br>HDL-C: coconut oil (48.7 ± 2.4) > soybean oil (45.0 ± 5.6)<br>TG: coconut oil (179.7 ± 93.7) > soybean oil (148.2 ± 64.8)<br>LDL-C:HDL-C ratio: soybean oil (3.1 ± 0.8) > coconut oil (2.41 ± 0.8) *

Cândido<br>(2021)	Randomized clinical trial (Brazil)	9 weeks	n = 52 women with BMI between 26 e 35 kg/m², %G > 30%<br>Age = 26.81 ± 0.74	Vitamin breakfast prepared with 25 ml of coconut oil, skimmed milk powder and some fruit flavoring, chocolate or cappuccino	Vitamin breakfast prepared with 25 ml of soybean oil, skimmed milk powder and some fruit flavoring, chocolate or cappuccino<br><br>Vitamin breakfast prepared with 25 ml of olive oil, skimmed milk powder and some fruit flavoring, chocolate or cappuccino	TC: coconut oil (173.50 ± 5.55) > olive oil (165.16 ± 6.22) > soybean oil (151.59 ± 5.81)<br>LDL-C: coconut oil (106.69 ± 4.79) > olive oil (95.89 ± 4.64) > soybean oil (85.82 ± 4.64)<br>HDL-C: olive oil (48.26 ± 2.27) > coconut oil (46.37 ± 2.54) > soybean oil (42.27 ± 3.28)<br>TG: olive oil (99.18 ± 8.56) >coconut oil (87 ± 7.20) > soybean oil (80.41 ± 8.35)<br>VLDL: olive oil (19.83 ± 1.71) > coconut oil (17.40 ± 1.44) > soybean oil (16.08 ± 1.67)
Chinwong (2017)	Randomized<br>crossover trial, open-label<br>(Thailand)	8 weeks	n = 32 healthy individuals<br>Age = 21 ± 0.7 years<br>BMI = 20.8 ± 3.4 kg/m²	30 ml/day of coconut oil extra virgin	30 ml/day of 2% carboxymethylcellulose (CMC) solution	TC: coconut oil (187.7 ± 34.5) > CMC solution (183.7 ± 33.7)<br>LDL-C: coconut oil (110.5 ± 30.5) > CMC solution (110.2 ± 32.0)<br>HDL-C: coconut oil (64.2 ± 9.9) > CMC solution (59.0 ± 10.2)*<br>TG: CMC solution (72.3 ± 28.5) > coconut oil (64.7 ± 23.5)

Cox<br><br>(1995)	Randomized<br><br>crossover trial<br><br>(New Zealand)	6 weeks	n = 28 individuals<br><br>TC: 5.5– 7.9 mmol/L<br><br>TG: <3 mmol/L<br><br>Age: 29 – 67 years<br><br>BMI = 25.1 ± 4.2 kg/m²	Three diets, each one followed for a 6-week period. Total fat supplied 36% of energy and carbohydrate 47% of energy.<br><br>Coconut diet: SFA from coconut oil supplied 20% of energy.	Butter diet: SFA from butter supplies ~20% of total energy.<br><br>Safflower diet: 10% of energy from safflower oil; SFA and PUFA each 10% of total energy.	TC: butter (263.0 ± 33.0) > coconut oil (249.0 ± 29.0) > safflower oil (233.0 ± 29.0) *<br><br>LDL-C: butter (175.0 ± 30.0) > coconut oil (163.0 ± 29.0) > safflower oil (151.0 ± 28.0) *<br><br>HDL-C: coconut oil (57.0 ± 15.0) > butter (56.0 ± 14.0) > safflower oil (54.0 ± 13.0)<br><br>TG: butter (177.0 ± 115.0) > coconut oil (159.0 ± 89.0) > safflower oil (151.0 ± 89.0)
Ganji<br><br>(1996)	Randomized<br><br>crossover trial<br><br>(EUA)	7 days	n = 10 healthy individuals<br><br>Age = 31.0 ± 5.0 years<br><br>BMI = 22.3 ± 1.7 kg/m²	Coconut oil was incorporated in the preparation of a loaf, with 42 g of coconut oil, making up 20% of the VCT. Participants should consume 1/3 of this bread in each of the three main meals.<br><br>Coconut oil plus psyllium fiber was incorporated in the preparation of a loaf, with 42 g of coconut oil, making up 20% of the VCT. Participants should consume 1/3 of this bread in each of the three main meals + 20 g of psyllium	Soybean oil was incorporated in the preparation of a loaf with 42 g of soybean oil, making up 20% of the VCT. Participants should consume 1/3 of this bread in each of the three main meals.	Coconut and soybean oil:<br><br>TC: coconut oil (204.9 ± 32.5) > soybean oil (191.0 ± 24.0) *<br><br>LDL-C: coconut oil (126.8 ± 30.2) > soybean oil (111.8 ± 23.2)<br><br>HDL-C: coconut oil (53.3 ± 0.3) > soybean oil (52.2 ± 8.5) *<br><br>TG: coconut oil (158.5 ± 53.1) > soybean oil (131.1 ± 39.0) *<br><br>VLDL: coconut oil (25.1 ± 13.1) > soybean oil (25.1 ± 10.0)<br><br>LDL-C/HDL-C ratio: coconut oil (2.40 ± 0.90) > soybean oil (2.20 ± 0.70)

				fiber per day divided into three equal doses.<br><br>Soybean oil was incorporated in the preparation of a loaf with 42 g of soybean oil, making up 20% of the VCT. Participants should consume 1/3 of this bread in each of the three main meals.<br><br>Soybean oil plus psyllium fiber was incorporated in the preparation of a loaf with 42 g of soybean oil, making up 20% of the VCT. Participants should consume 1/3 of this bread in each of the three main meals + 20 g of psyllium fiber per day divided into three equal doses.		Coconut and soybean oil + psyllium fiber:<br><br>TC: coconut oil (192.6 ± 28.2) > soybean oil (177.1 ± 32.1) *<br><br>LDL-C: coconut oil (112.5 ± 28.2) > soybean oil (100.5 ± 28.2) *<br><br>HDL-C: coconut oil (53.2 ± 9.7) > soybean oil (53.7 ± 8.9)<br><br>TG: coconut oil (141.7 ± 47.9) > soybean oil (134.6 ± 54.0) VLDL: coconut oil (26.2 ± 12.0) > soybean oil (23.9 ± 8.1)<br><br>LDL-C/HDL-C ratio: coconut oil (2.2 ± 0.6) > soybean oil (1.8 ± 0.5)*
Harris<br><br>(2017)	Randomized<br><br>crossover trial<br><br>(EUA)	4 weeks	n = 12 postmenopausal women<br><br>Age = 57.8 ± 3.7 years<br><br>BMI = 26.4 ± 4.4 kg/m²	Ingestion of 30 ml of coconut oil per day in ready-made preparations (smoothies-like beverages or in the preparation of salad dressings).	Ingestion of 30ml of safflower oil per day in ready-made preparations (smoothies-like beverages or in the	TC: coconut oil (237.8 ± 24.1) > safflower oil (219.3 ± 22.8)*<br><br>LDL-C: coconut oil (137.5 ± 27.2) > safflower oil (126.8 ± 25.7)*

					preparation of salad dressings).	HDL-C: coconut oil (70.5 ± 18.8) > safflower oil (62.9 ± 14.5)*<br>TG: safflower oil (118.3 ± 112.7) > coconut oil (107.5 ± 80.6)<br>CT:HDL-C ratio: safflower oil (3.8 ± 1.2) > coconut oil (3.7 ± 1.3)
Heber<br>(1992)	Randomized<br>crossover trial<br>(USA)	3 weeks	n = 9 healthy men	35% of the calories of the day were derived from LIP and of these, 50% were from coconut oil, which was incorporated into muffins or biscuits. Each muffin or cookie provided 13.7g of LIP for the oil test.	35% of the calories of the day were derived from LIP and of these, 50% were from palm oil or hydrogenated soybean oil which was incorporated into muffins or biscuits. Each muffin or cookie provided 13.7g of LIP for the oil test.	TC: coconut oil (195.0 ± 21.0) > palm oil (173.0 ± 21.0) > hydrogenated soybean oil (168.0 ± 15.0)*<br>LDL-C: coconut oil (129.0 ± 24.0) > palm oil (115.0 ± 21.0) > hydrogenated soybean oil (111.0 ± 18.0)*<br>HDL-C: coconut oil (42.1 ± 12.0) > palm oil (41.0 ± 15.0) > hydrogenated soybean oil (39.0 ± 9.0)<br>TG: coconut oil (110.0 ± 69.0) > hydrogenated soybean oil (104.0 ± 60.0) > palm oil (79.0 ± 18.0)
Khaw<br>(2018)	Randomized clinical trial<br>(UK)	4 weeks	n = 94 healthy individuals<br>Age = 59.9 ± 6.1 years<br>BMI = 25.1 ± 4.2 kg/m²	Coconut oil: 50 g of coconut oil incorporated in the usual daily diet in substitution of other fats or ingested as a supplement.	Butter: 50 g of butter incorporated in the usual daily diet in substitution of other fats or ingested as a supplement.	TC: coconut oil (239.7 ± 34.8) ><br>butter = olive oil (232.0 ± 38.7)*<br>LDL-C: butter (146.9 ± 35) > olive oil (139.2 ± 39.0)> coconut oil (131.5 ± 35.0)*

					Olive oil: 50 g of olive oil incorporated in the usual daily diet in substitution of other fats or ingested as a supplement.	HDL-C: coconut oil (88.9 ± 27.0) > olive oil = butter (77.3 ± 23.2)*<br>TG: coconut oil (97.4 ± 70.8) > olive oil (97.4 ± 53.1) > butter (88.6 ± 44.3)<br>CT/HDL-C ratio: olive oil (3.3 ± 1.2) > butter (3.3 ± 0.9) > coconut oil (2.9 ± 0.9)*
Lu<br>(1997)	Randomized<br>crossover trial<br>(EUA)	3 weeks	n = 15 healthy women<br>Age = 20.0 ± 2.0 years<br>BMI = 22.6 ± 2.4 kg/m²	Coconut oil: 10% of daily VCT from coconut oil	A16 oil: 10% of daily VCT from oil A16 (transgenic soybean oil, composed of a lower ratio of 18: 3 without trans fats)<br><br>Soybean oil: 10% of daily VCT from soybean oil	TC: A16 oil [-19.7 ± 20.9] > soybean oil<br>[-13.5 ± 20.1] > coconut oil [-9.2 ± 14.7]<br>LDL-C: A16 oil [-10.0 ± 19.7] > soybean oil<br>[-4.2 ± 20.1] > coconut oil [-2.3 ± 15.8]<br>HDL-C: A16 oil [-8.1 ± 7.0] > soybean oil [-7.0 ± 5.4] > coconut oil [-3.5 ± 8.1]*<br>TG: coconut oil [-13.3 ± 26.6] > A16 oil<br>[-9.7 ± 29.2] > soybean oil [-8.8 ± 28.3]<br>LDL-C/HDL-C ratio: A 16 oil = soybean oil [0.1 ± 0.4] > coconut oil [0.1 ± 0.3]

McKenney<br><br>(1995)	Randomized<br><br>crossover trial<br><br>(EUA)	6 weeks	n = 11 individuals with TC altered<br><br>Age = 58.0 ± 8 years	Coconut oil was added as the main ingredient in oat biscuits with raisins.	Canola oil has been added as the main ingredient in oat biscuits with raisins.	TC: coconut oil (233.3 ± 19.0) > canola oil (213.1 ± 23.4)*<br><br>LDL-C: coconut oil (155.4 ± 19.5) > canola oil (138.1 ± 17.0)*<br><br>HDL-C: coconut oil (53.9 ± 15.9) > canola oil (51.7 ± 15.5)*<br><br>TG: canola oil (121.3 ± 54.2) > coconut oil (120.0 ± 47.7)*<br><br>CT/HDL-C ratio: coconut oil (4.30 ± 1.10) > canola oil (4.10 ± 0.80)
Maki<br><br>(2018)	Randomized crossover trial<br><br>(EUA)	4 weeks	n = 25 individuals<br><br>Age = 45.2 ± 2.3 years<br><br>BMI = 27.7 ± 0.8	Consumption of four products made with coconut oil per day, which could be three types of muffins and three types of rolls. Each product was made with one tablespoon of coconut oil (13.6 g), consisting on consumption of 54.4 g of oil per day.	Consumption of four products made with corn oil per day, which could be three types of muffins and three types of rolls. Each product was made with one tablespoon of corn oil (13.6 g), consisting on consumption of 54.4 g of oil per day.	TC: coconut oil [7.1, IC95%: -1.1; 13.1] > corn oil [-0.5, IC95%: -5.7; 9.7]*<br><br>LDL-C: coconut oil [4.6, IC95%: -2,5; 17.5] > corn oil [-2.7, IC95%: -8.9; 11.5]<br><br>HDL: coconut oil [6.5, IC95%: 2.7; 17.8] > corn oil [5.4, IC95%: 1.4; 10.3]*<br><br>TC:HDL-C: corn oil [-4.3 IC95%: -11.7; 1.8] > coconut oil [-3.3, IC 95%: -15; 2.8]*<br><br>TG: coconut oil [6, IC 95%: -3.0; 13.2] > corn oil [-2.1, IC 95%: -9.7; 20.6]

Oliveira-de-Lira (2018)	Randomized Clinical Trial<br>(Brazil)	8 weeks	n = 75 obese women<br>Age = 34.07 ± 5.4 years	Coconut oil: 6 ml/day supplemented in capsules 30 min before main meals.	Safflower oil: 6 ml/day supplemented in capsules 30 min before main meals.<br><br>Chia oil: 6 ml/day supplemented in capsules 30 min before main meals<br><br>Soybean oil: 6 ml/day supplemented in capsules 30 min before main meals.	TC: coconut oil (198.0 ± 17.6) > soybean oil (195.7 ± 26.2) > chia oil (187.1 ± 17.0) > safflower oil (182.9 ± 19.1)*<br>LDL-C: safflower oil (130.6 ± 24.3) > coconut oil (128.3 ± 17.7) > soybean oil (127.5 ± 23.2) > chia oil (123.6 ± 18.2)*<br>HDL-C: coconut oil (55.6 ± 6.4) > soybean oil (49.9 ± 7.1) > chia oil (49.0 ± 5.9) > safflower oil (47.1 ± 10.0)*<br>TG: soybean oil (107.5 ± 39.2) > coconut oil (98.3 ± 29.1) > safflower oil (93.9 ± 36.5) > chia oil (88.0 ± 24.4)*<br>VLDL: soybean oil (20.0 ± 8.0) > chia oil (18.0 ± 5.1) > coconut oil (17.8 ± 3.2) > safflower oil (15.7 ± 4.5)
Reiser<br>(1985)	Randomized<br>crossover trial<br>(USA)	5 weeks	n = 19 normolipidemic<br>male medical students<br>(12 completed all three diets)	35% of total energy from fat,<br>being 60% fat from coconut oil,<br>lard, or safflower oil	Habitual diet at baseline and during washout periods	TC: coconut oil (168.0 ± 3.0) > lard (155.0 ± 3.0) > safflower oil (141.0 ± 3.1)*<br>LDL-C: coconut oil (110.0 ± 4.1) > lard (98.0 ± 4.5) > safflower oil (90.0 ± 4.7)*<br>HDL-C: coconut oil (46.0 ± 1.1) > lard = safflower oil (40.0 ± 1.2) *

						TG: lard (88.0 ± 3.5) > coconut oil (78 ± 3.6) > safflower oil (72.0 ± 3.7)*
Schwab<br><br>(1994)	Randomized crossover clinical trial (Finland)	4 weeks	n = 15 healthy women<br><br>Age = 23.9 ± 4.6 years<br><br>BMI = 21.4 ± 1.9 kg/m²	Refined coconut oil (16 to 26 g/day of coconut oil = 4% of the daily VCT). This diet still contained oils from other sources: rapeseed oil (5 to 8g/day), olive oil (3 to 4.5g/day) and sunflower oil (2 to 3.5g/day).	Refined palm oil bleached and deodorized (22 to 33 g/day of palm oil = 4% of daily VCT). This diet still contained soybean oil (2 to 5 g/day) as a source of fat.	TC: palm oil (189.9 ± 28.5) > coconut oil (187.5 ± 24.1)<br><br>LDL-C: palm oil (113.3 ± 19.5) > coconut oil (110.2 ± 18.0)<br><br>HDL-C: palm oil (58.8 ± 12.0) > coconut oil (57.6 ± 10.5)<br><br>TG: coconut oil (77.1 ± 30.9) > palm oil (77.1 ± 27.4)<br><br>VLDL: coconut oil (19.7 ± 7.5) > palm oil (17.8 ± 7.5) *
Vijayakumar<br><br>(2015)	Randomized clinical<br><br>trial<br><br>(India)	2 years	n = 198 individuals with CVD<br><br>Age = 59.0 ± 8.7 years<br><br>BMI = 24.7 ± 4.7 kg/m²	15% of the daily VCT of a trademark coconut oil to be used as cooking oil.	15% of the daily VCT of a trademark sunflower oil to be used as cooking oil.	TC: sunflower oil (151.6 ± 44.5) > coconut oil (149.3 ± 28.6)<br><br>LDL-C: coconut oil (91.0 ± 21.9) > sunflower oil (89.6 ± 29.0)<br><br>HDL-C: sunflower oil (44.4 ± 16.3) > coconut oil (43.2 ± 10.8)<br><br>TG: sunflower oil (112.2 ± 45.1) > coconut oil (109.3 ± 47.1)<br><br>VLDL: sunflower oil (22.5 ± 9.7) > coconut oil (21.8 ± 9.4)

Vogel<br>(2020)	Randomized clinical trial<br>(Brazil)	45 days	n = 29 mens with obesity I<br>Age = between 20–59 years	Addition of 1 tablespoon (12 ml) of coconut oil<br>to dinner	Addition of 1 tablespoon (12 ml) of soybean oil to dinner	TC: soybean oil (177.07 ± 39.44) > coconut oil (171.47 ± 49.44)<br>LDL-C: soybean oil (116.29 ± 26.55) > coconut oil (101 ± 37.17)<br>HDL-C: coconut oil (43.07 ± 14.86) >soybean oil (35.93 ± 7.77)<br>TG: coconut oil (138.87 ± 78.28) > soybean oil (119.50 ± 74.13)<br>VLDL: coconut oil (27.53 ± 15.74) > soybean oil (24.85 ± 16.82)<br>TC: HDL-C: soybean oil (5.07 ± 1.35) > coconut oil (4.30 ± 1.58)
Voon<br>(2011)	Randomized<br>crossover trial<br>(Malaysia)	5 weeks	n = 45 normal and overweight healthy adults<br>Age: 30.1 ± 8.3 years<br>BMI = 23.1 ± 3.7 kg/m²	Meals with 30% energy from fat, two-thirds of which was from coconut oil (20% total energy)	Meals with 30% energy from fat, two-thirds of which was from palm oil or extra virgin olive oil (20% total energy)	TC: coconut oil (191.4 ± 26.7) > palm oil (186.0 ± 28.6) > olive oil (179.8 ± 27.5)*<br>LDL-C: coconut oil (127.6 ± 29) > palm oil (123.7 ± 27.5) > olive oil (118.3 ± 24.7)*<br>HDL-C: coconut oil (53.0 ± 11.6) > palm oil (50.6 ± 10.0) > olive oil (49.5 ± 8.9)*<br>TG: coconut oil (79.7 ± 34.5)> palm oil (75.3 ± 27.5) > olive oil (74.4 ± 32.8)

						TC:HDL-C ratio: palm oil (3.69 ± 0.90) > coconut oil (3.65 ± 0.95) > olive oil (3.63 ± 0.93)

* Significantly different (P<0.05). BMI: body mass index; TC: total cholesterol; LDL-C: low density lipoprotein; HDL-C: high density lipoprotein; TG: triglycerides, SFA: saturated fatty acid; VCT: total caloric value; LIP: lipids; MUFA: monounsaturated fatty acid; PUFA: polyunsaturated fatty acid; CVD: cardiovascular disease

Table S7. Grading of Recommendations Assessment, Development and Evaluations (GRADE) - Coconut oil compared to other oils, fat or placebo for health outcomes

Certainty assessment							Summary of findings				
							Study event rates (%)			Anticipated absolute effects	
Participants (studies)	Risk of bias	Inconsistency	Indirectness	Imprecision	Other	Overall certainty of evidence	With other oils, fat or placebo	With Coconut oil	Relative effect (95% CI)	Risk with other oils, fat or placebo	Risk difference with Coconut oil
**LDL-C**											
515 (7 RCTs)	very serious[a]	serious[b]	not serious	serious[c]	none	⊕◯◯◯ Very low	304	211	-	The mean lDL-c was **0** mg/dL	MD **1.67 mg/dL lower (6.93 lower to 3.59 higher)**

**HDL-C**											
515 (7 RCTs)	very serious[d]	serious[e]	not serious	not serious	none	⨁◯◯◯ Very low	304	211	-	The mean hDL-c was **0** mg/dL	MD **3.28 mg/dL higher (0.66 higher to 5.9 higher)**
**Triglycerides**											
515 (7 RCTs)	very serious[f]	not serious	not serious	serious[g]	none	⨁◯◯◯ Very low	304	211	-	The mean triglycerides were **0** mg/dL	MD **0.24 mg/dL lower (5.52 lower to 5.04 higher)**
**Body weight**											
486 (6 RCTs)	very serious[h]	serious[i]	not serious	not serious	none	⨁◯◯◯ Very low	290	196	-	The mean body weight was **0** kg	MD **0.24 kg lower (0.83 lower to 0.34 higher)**
**Waist circumference**											
287 (4 RCTs)	very serious[j]	serious[k]	not serious	not serious	none	⨁◯◯◯ Very low	190	97	-	The mean waist circumference was **0** cm	MD **0.64 cm lower (1.69 lower to 0.41 higher)**

Total body fat

445 (5 RCTs)	very serious[l]	serious[m]	not serious	not serious	none	⨁◯◯◯ Very low	269	176	-	The mean total body fat was **0** %	MD **0.10 % lower (0.56 lower to 0.36 higher)**

Fasting blood glucose

212 (4 RCTs)	very serious[n]	not serious	not serious	serious[o]	none	⨁◯◯◯ Very low	133	79	-	The mean total fasting blood glucose was **0** mg/dL	MD **0.82 mg/dl lower (1.18 lower to 2.82 higher)**

US-CRP

131 (2 RCTs)	very serious[p]	not serious	not serious	not serious	none	⨁◯◯◯ Very low	83	48	-	The mean total USC-RP was **0** mg/dL	MD **0.04 mg/dl lower (0.91 lower to 0.82 higher)**

CI: confidence interval; **MD:** mean difference

Explanations

a. RCTs are at risk of bias due to: blinding of participants and/or outcome (in Khaw et al. and Vogel et al.) and selective reporting (in Schwab et al.). RCTs present an unclear risk of bias in: randomization (in Oliveira-de-Lira et al., Schwab et al., Vijayakumar et al.), allocation (in Assunção et al., Oliveira-de-Lira et al., Schwab et al., Vijayakumar et al., Vogel

et al.), participant blinding and/or outcome (in Candido et al., Khaw et al., Schwab et al., Vijayakumar et al., Vogel et al.) and selective reporting (in Assunção et al., Oliveira-de-Lira et al., Vijayakumar et al.).

b. Large amounts of statistical heterogeneity (I^2:78%); point estimates and confidence intervals vary considerably.

c. Imprecision due to wide confidence interval: in the worst scenario, it may increase 3.59 mg/dL; in the best scenario, it may decrease 6.93 mg/dL.

d. RCTs are at risk of bias due to: blinding of participants and/or outcome (in Khaw et al. and Vogel et al.) and selective reporting (in Schwab et al.). RCTs present an unclear risk of bias in: randomization (in Oliveira-de-Lira et al., Schwab et al., Vijayakumar et al.), allocation (in Assunção et al., Oliveira-de-Lira et al., Schwab et al., Vijayakumar et al., Vogel et al.), participant blinding and/or outcome (in Candido et al., Khaw et al., Schwab et al., Vijayakumar et al., Vogel et al.) and selective reporting (in Assunção et al., Oliveira-de-Lira et al., Vijayakumar et al.).

e. Large amounts of statistical heterogeneity (I^2:74%); point estimates and confidence intervals vary considerably.

f. RCTs are at risk of bias due to: blinding of participants and/or outcome (in Khaw et al. and Vogel et al.) and selective reporting (in Schwab et al.). RCTs present an unclear risk of bias in: randomization (in Oliveira-de-Lira et al., Schwab et al., Vijayakumar et al.), allocation (in Assunção et al., Oliveira-de-Lira et al., Schwab et al., Vijayakumar et al., Vogel et al.), participant blinding and/or outcome (in Candido et al., Khaw et al., Schwab et al., Vijayakumar et al., Vogel et al.) and selective reporting (in Assunção et al., Oliveira-de-Lira et al., Vijayakumar et al.).

g. Imprecision due to wide confidence interval: in the worst scenario, it may increase 5.04 mg/dL; in the best scenario, it may decrease 5.52 mg/dL.

h. RCTs are at risk of bias due to: blinding of participants and/or outcome (in Khaw et al. and Vogel et al.) and selective reporting (in Schwab et al.). RCTs present an unclear risk of bias in: randomization (in Oliveira-de-Lira et al., Schwab et al., Vijayakumar et al.), allocation (in Assunção et al., Oliveira-de-Lira et al., Schwab et al., Vijayakumar et al., Vogel et al.), participant blinding and/or outcome (in Candido et al., Khaw et al., Schwab et al., Vijayakumar et al., Vogel et al.) and selective reporting (in Assunção et al., Oliveira-de-Lira et al., Vijayakumar et al.).

i. Large amounts of statistical heterogeneity (I^2:76%); point estimates and confidence intervals vary considerably.

j. RCTs are at risk of bias due to: blinding of participants and/or outcome (in Khaw et al. and Vogel et al.) and selective reporting (in Schwab et al.). RCTs present an unclear risk of bias in: randomization (in Oliveira-de-Lira et al., Schwab et al., Vijayakumar et al.), allocation (in Assunção et al., Oliveira-de-Lira et al., Schwab et al., Vijayakumar et al., Vogel et al.), participant blinding and/or outcome (in Candido et al., Khaw et al., Schwab et al., Vijayakumar et al., Vogel et al.) and selective reporting (in Assunção et al., Oliveira-de-Lira et al., Vijayakumar et al.).

k. Large amounts of statistical heterogeneity (I^2:80%); point estimates and confidence intervals vary considerably.

l. RCTs are at risk of bias due to: blinding of participants and/or outcome (in Khaw et al. and Vogel et al.). RCTs present an unclear risk of bias in: randomization (in Oliveira-de-Lira et al., Vijayakumar et al.), allocation (in Oliveira-de-Lira et al., Vijayakumar et al., Vogel et al.), participant blinding and/or outcome (in Candido et al., Khaw et al., Vijayakumar et al., Vogel et al.) and selective reporting (in Oliveira-de-Lira et al. and Vijayakumar et al.).

m. Large amounts of statistical heterogeneity (I^2:75%); point estimates and confidence intervals vary considerably.

n. RCTs are at risk of bias due to: blinding of participants and/or outcome (in Khaw et al. and Vogel et al.). RCTs present an unclear risk of bias in: allocation (in Assunção et al., and Vogel et al.), participant blinding and/or outcome (in Candido et al., Khaw et al. and Vogel et al.) and selective reporting (in Assunção et al.).

o. Imprecision due to wide confidence interval: in the worst scenario, it may increase 2.62 mg/dL; in the best scenario, it may decrease 1.18 mg/dL.

p. RCTs are at risk of bias due to: blinding of participants and/or outcome (in Khaw et al.). RCTs present an unclear risk of bias in: allocation (in Assunção et al.), participant blinding and/or outcome (in Khaw et al.) and selective reporting (in Assunção et al.).

Appendix II - Additional results

Lipid Profile

LDL-C to HDL-C ratio and TC: HDL-C ratio

Three studies analyzed the effects of coconut oil on LDL-C to HDL-C ratio (n=65, 92% female, 18 to 36 years) [1-3]. The consumption of coconut oil reduced the LDL-C/HDL-C ratio in comparison to soybean and transgenic soybean oils [1,2]. Seven studies [4-10] analyzed the effects of coconut oil on TC: HDL-C ratio. These studies included 291 participants (70% females, 34 to 68 years).

Three studies [4,5,10] were included in the meta-analysis regarding TC:HDL-C ratio (-0.12; CI 95% -0.43 to 0.20; figure 1). We performed a subgroup analysis excluding a study that used a SAFs rich oil/fat as a comparator and the results did not change (vs butter**;** -0.06; CI 95% -0.46 to 0.34; figure 2) [5].

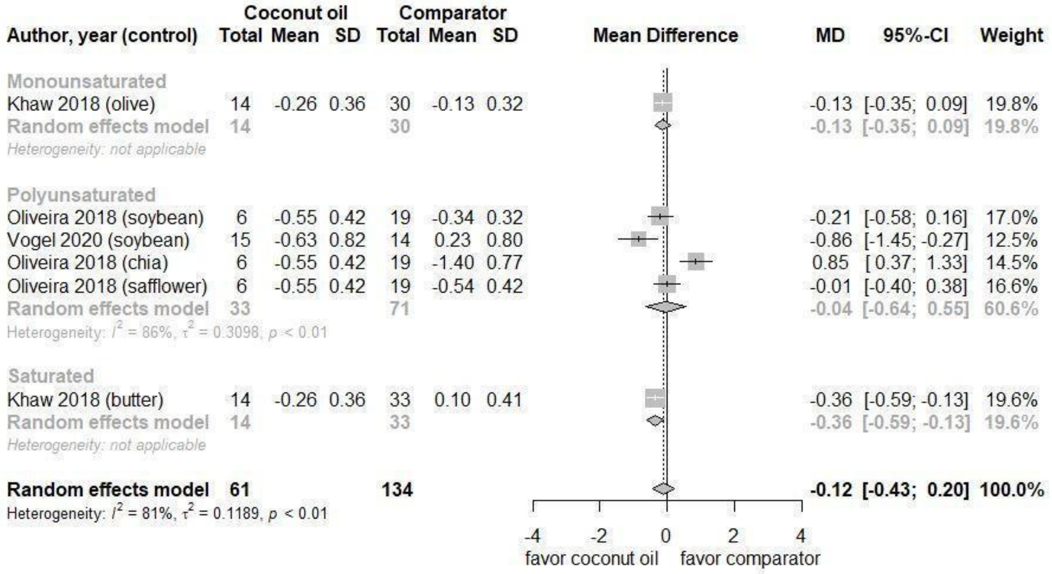

Appendix II - Figure 1. Forest plots of randomized controlled clinical trials investigating the effects of coconut oil intake on TC:HDL-C ratios

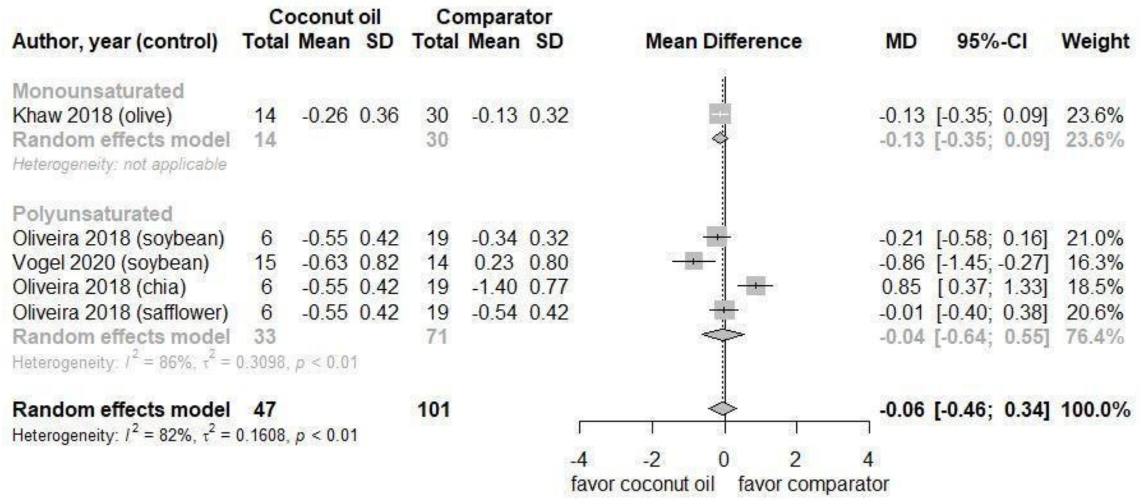

Appendix II - Figure 2. Forest plots of randomized controlled clinical trials investigating the effects of coconut oil intake vs MUFA and PUFA rich oils on TC:HDL-C ratios

Glycemic profile

Fasting blood glucose

Seven studies analyzed the effects of coconut oil on fasting glucose levels [2, 5, 6, 10-13]. These studies included 297 participants (69.3% females, 23 to 66 years). Four studies [2,5,10,13] were included in the meta-analysis. Overall, the effect of coconut oil intake on fasting glucose levels in comparison to other oils/fats did not differ (0.82 mg/dL; 95% CI -1.18 to 2.82 mg/dL; figure 3). We performed a subgroup analysis excluding a study that used a SAFs rich oil/fat as a comparator and the results did not change (vs butter 1.14 mg/dL; 95% CI -1.01 to 3.29 mg/dL; figure 4) [1]. The effect of coconut oil on fasting plasma glucose did not differ in comparison to PUFAs (0.37 mg/dL; 95% CI -3.37 to 4.12 mg/dL) and MUFAs (1.91 mg/dL; 95% CI -1.48 to 5.30 mg/dL).

A crossover study (n=9) [11] demonstrated that consumption of coconut oil increases blood glucose levels more than palm oil, but less than hydrogenated soybean oil.

HbA1c

Two studies analyzed this outcome (n=273, 32% female, 29 to 68 years) [4,14]. An 8-week study found significantly lower values of HbA1c when comparing coconut oil to PUFAs [4]. A

2-year follow-up study compared the consumption of coconut oil with PUFAs and found no difference between groups [14]. Results are shown in table S2.

Effects of coconut oil on insulin levels, β-cell function and indices of insulin sensitivity

A study observed that coconut oil increased β-cell function and insulin sensitivity in comparison to the consumption of soybean oil (n=40, 100% female, 29.8 ± 6.6 years, follow-up: 12 weeks) [2]. Results are shown in table S2.

One study analyzed the insulin resistance index (HOMA-IR), comparing coconut oil with soy oil, but found no difference between groups (n=29, 100% man, 35.27 ± 11.12 coconut oil group and 39.28 ± 9.06 soybean oil group, follow-up: 45 days) [10].

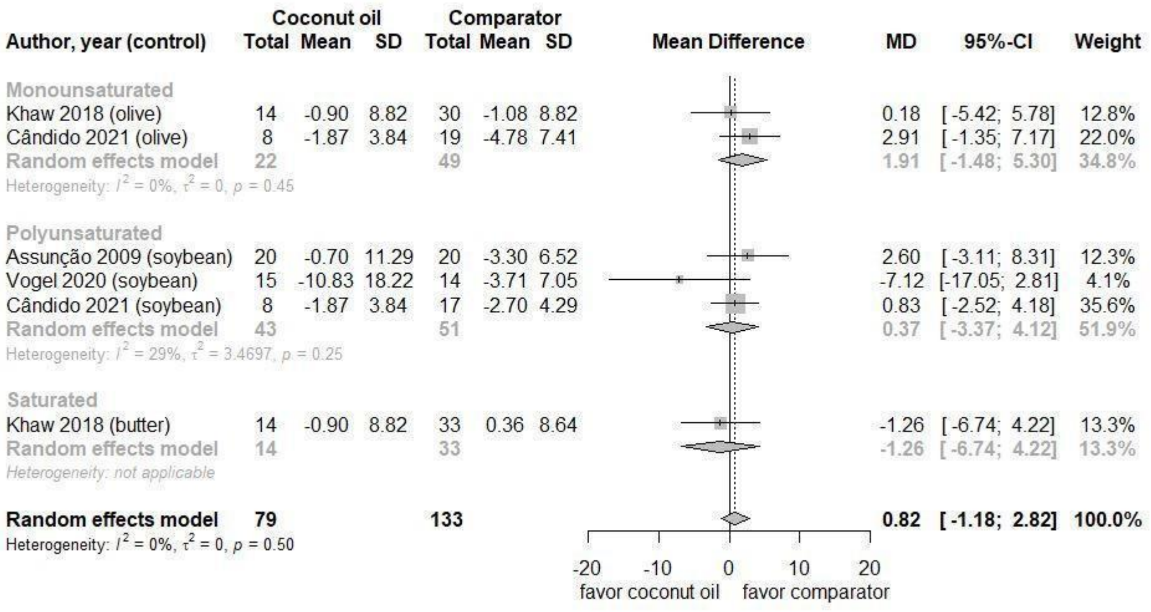

Appendix II - Figure 3. Forest plots of randomized controlled clinical trials investigating the effects of coconut oil intake on fasting blood glucose (mg/dL)

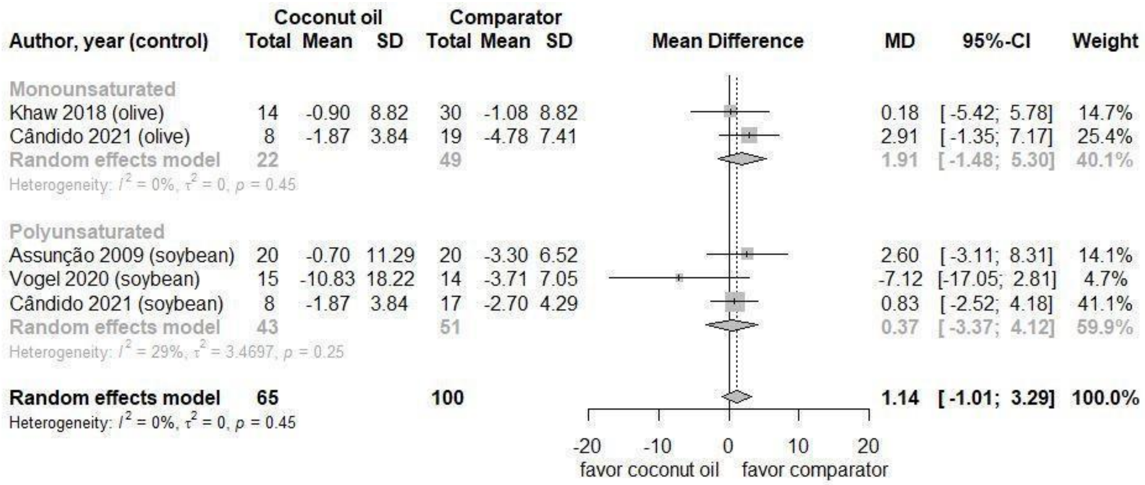

Appendix II - Figure 4. Forest plots of randomized controlled clinical trials investigating the effects of coconut oil intake vs PUFA and MUFA rich oils on fasting blood glucose (mg/dL).

Blood pressure

Systolic Blood Pressure

Two studies [5, 15] analyzed this outcome (n=126, 63% female, 20 to 66 years, follow-up of 4 to 8 weeks). When comparing the effect of coconut oil intake with placebo, higher levels of systolic blood pressure are observed [15]. However, when the effect of the intake of coconut oil is compared with olive oil or butter, lower levels of systolic blood pressure are observed [5]. We were not able to meta-analyze these data, since one study was a crossover trial and there was not enough data. Results are shown in table S3.

Diastolic Blood Pressure

Two studies [5,15] analyzed this outcome (n=126, 63% female, 20 to 66 years, follow-up of 4 to 8 weeks). When the effect of the intake of coconut oil is compared with placebo, olive oil and butter, higher levels of diastolic blood pressure are observed [5,15]. We were not able to meta-analyze these data, since one study was a crossover trial and there was not enough data. Results are shown in table S3.

Inflammatory profile

Four studies (follow-up 4 weeks to 2 years, n=377, 40% females, 22 to 68 years) analyzed the effects of coconut oil on US-CRP [2,5,6,14].

Two studies [2,5] were included in the meta-analysis. Overall, the effect of coconut oil intake on US-CRP in comparison to other oils/fats did not differ (-0.04 mg/dL; 95% CI -0.91 to 0.82

mg/dL; figure 5). A crossover study observed lower levels of US-CRP with the intake of coconut oil when compared to olive and palm oils [6].

One RCT study (follow-up 12 weeks, n=40, 100% female, 23.9 ± 4.6 years) analyzed the effects of coconut oil in fibrinogen. Coconut oil increased fibrinogen when compared to consumption of soybean oil [2].

A crossover study (follow-up 5 weeks, n=45, 80% female, 30.1 ± 8.3 years) observed that coconut oil consumption increased tcHcy, IL-1β, IL-6, IL-8 and *IFN- γ* when compared to the use of palm and extra virgin olive oil [6]. Results are shown in table S4.

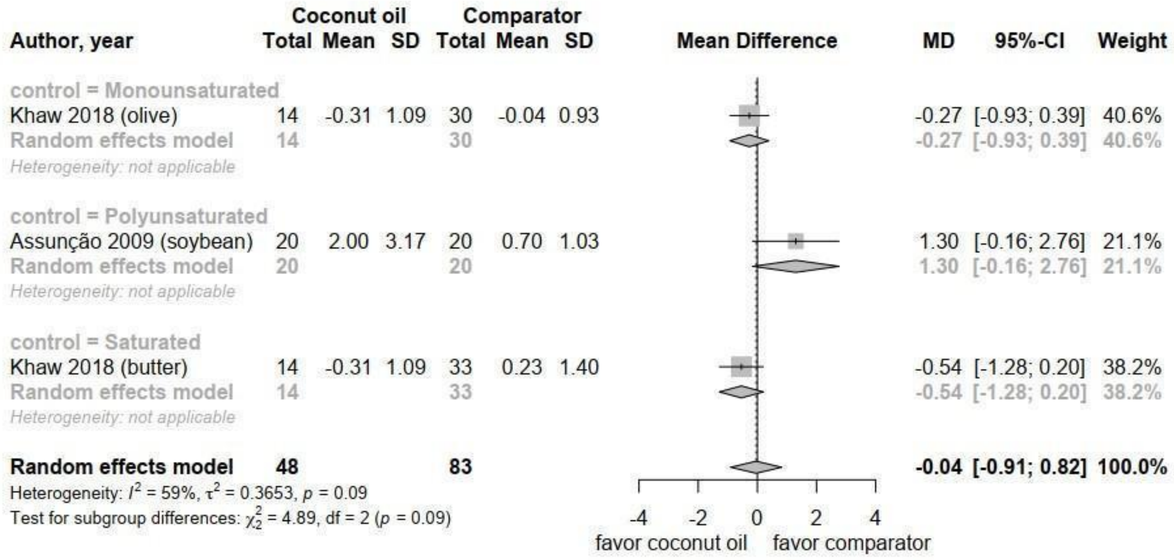

Appendix II - Figure 5. Forest plots of randomized controlled clinical trials investigating the effects of coconut oil intake on US-CRP (mg/dL)

Supplementary figures

Figure S1. Forest plot of randomized controlled clinical trials investigating the effects in body weight (kg) of coconut oil intake versus PUFA and MUFA rich oils

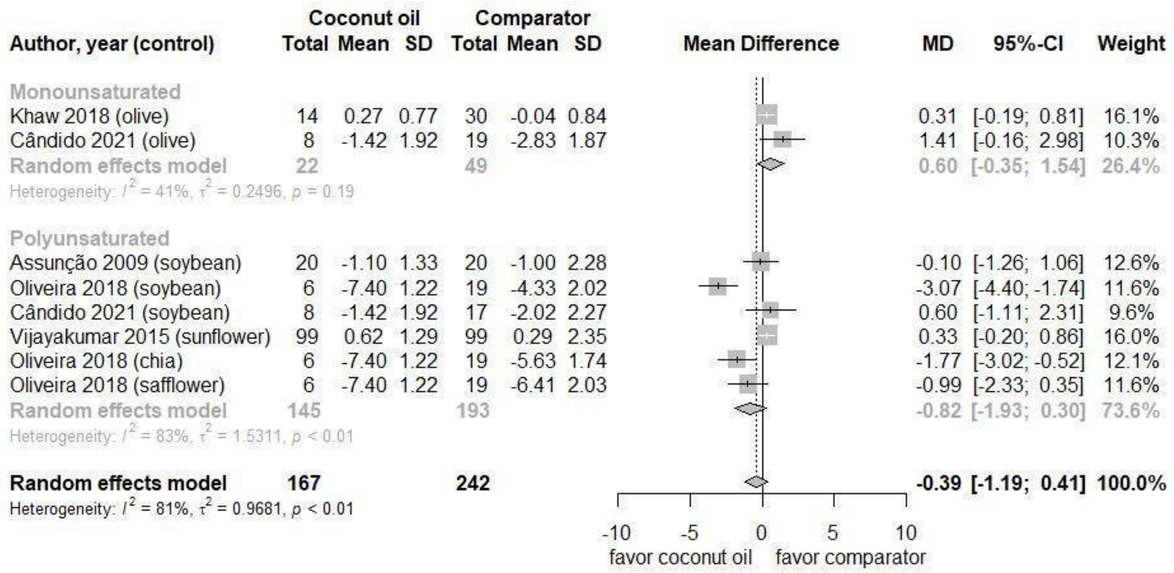

Figure S2. Forest plot of randomized controlled clinical trials investigating the effect in body weight (kg) of coconut oil versus olive oil

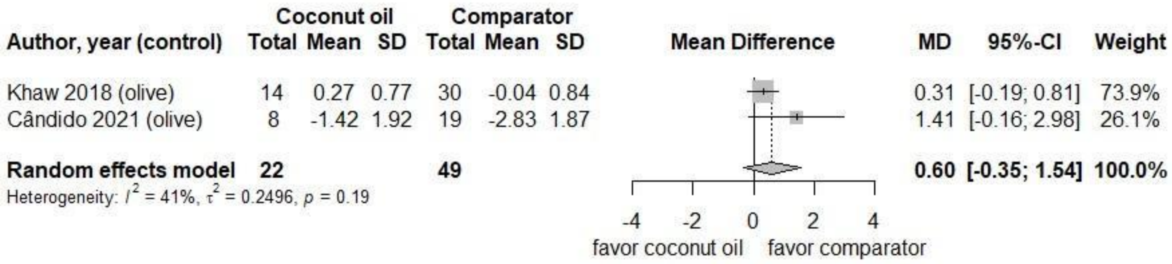

Figure S3. Forest plot of randomized controlled clinical trials investigating investigating the effect in body weight (kg) of coconut oil versus soybean oil

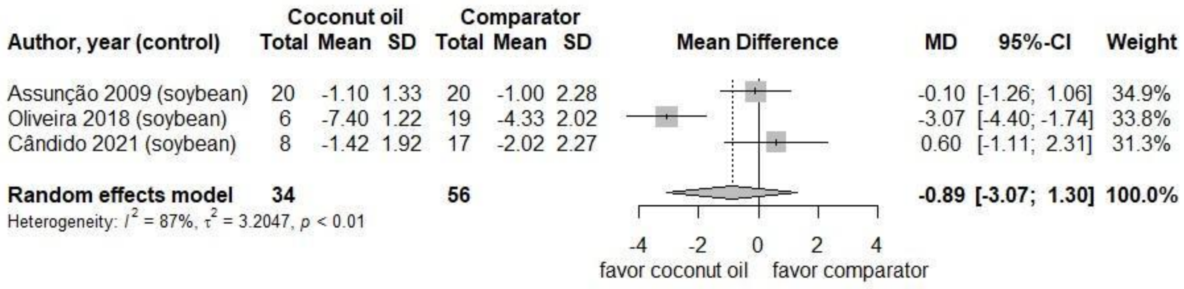

Figure S4. Forest plot of randomized controlled clinical trials investigating the effect in body weight (kg) of coconut oil versus other oils in studies carried out in women

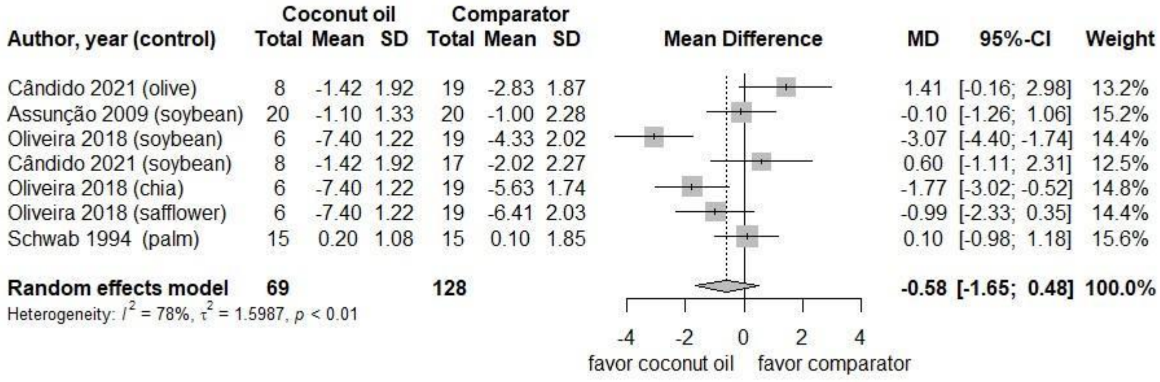

Figure S5. Forest plot of the randomized controlled clinical trials investigating the effect of coconut oil versus other oils in body weight (kg) of studies conducted in Brazil

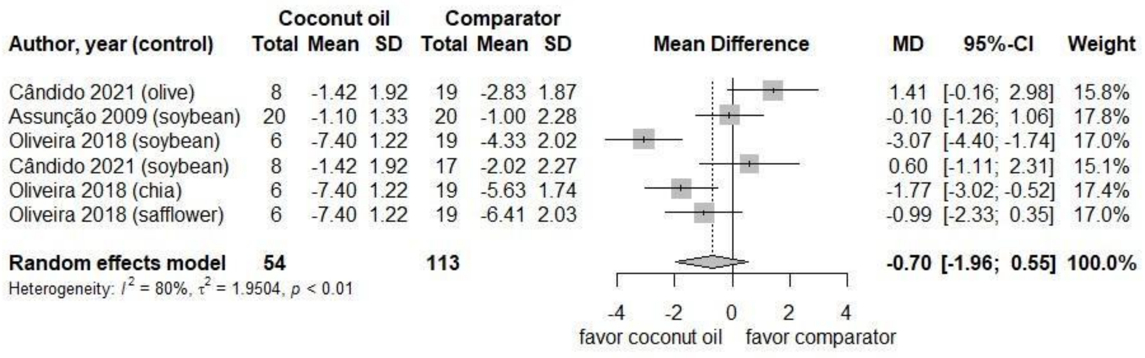

Figure S6. Forest plot of randomized controlled clinical trials investigating the effect of coconut oil versus other oils/fats in body weight (kg) of studies carried out in patients with overweight/obesity

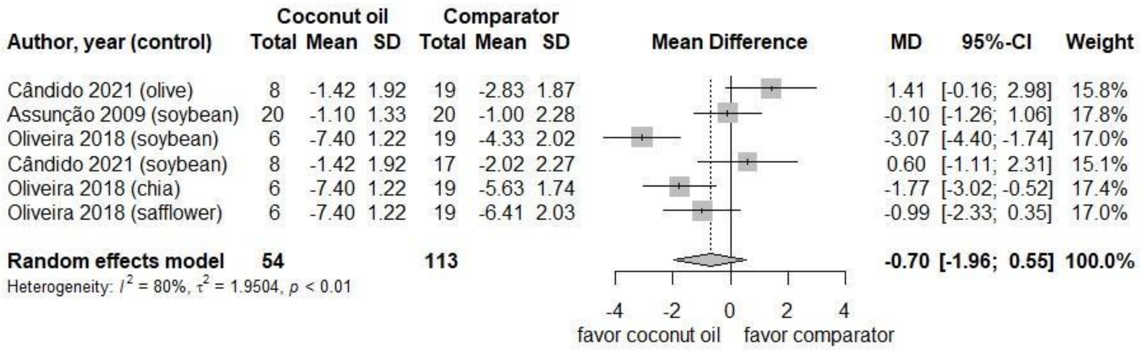

Figure S7. Forest plot of randomized controlled clinical trials investigating the effect on body weight (kg) of coconut oil versus other oils/fats without the long term study of Vijayakumar et al.

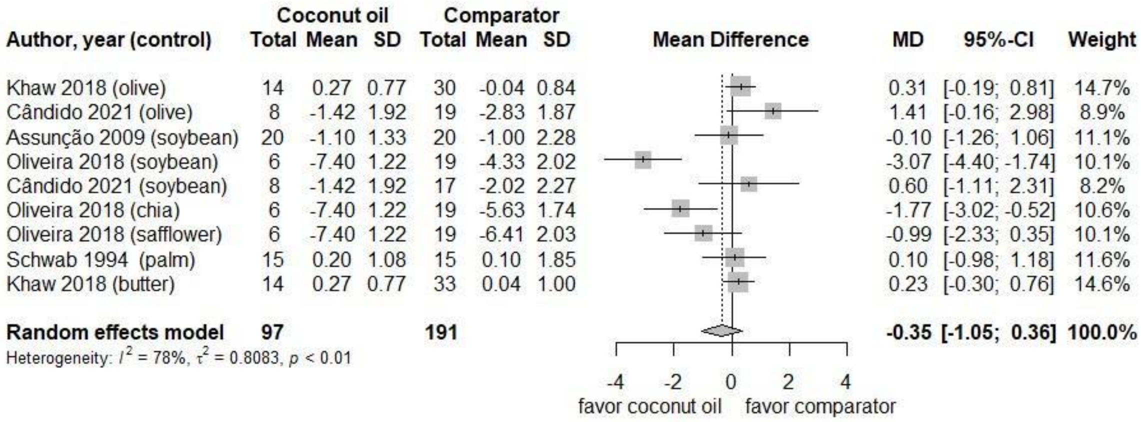

Figure S8. Forest plot of randomized controlled clinical trials investigating the effect in body weight (kg) of coconut oil versus other oils/fats in studies including co-intervention

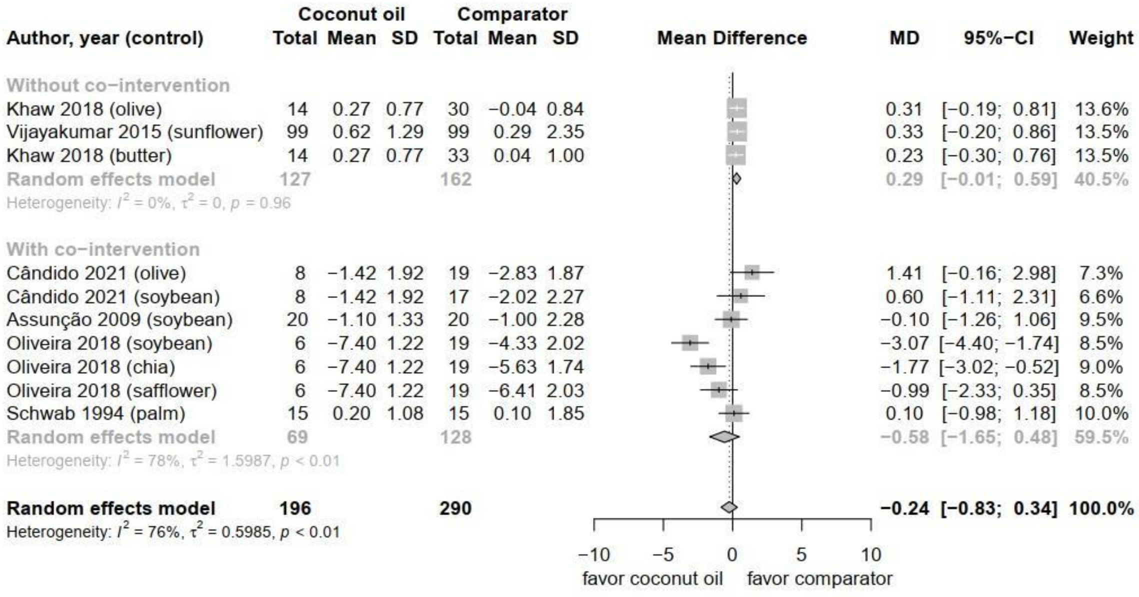

Figure S9. Forest plot of randomized controlled clinical trials investigating the effects in waist circumference (cm) of coconut oil intake versus PUFA and MUFA rich oils

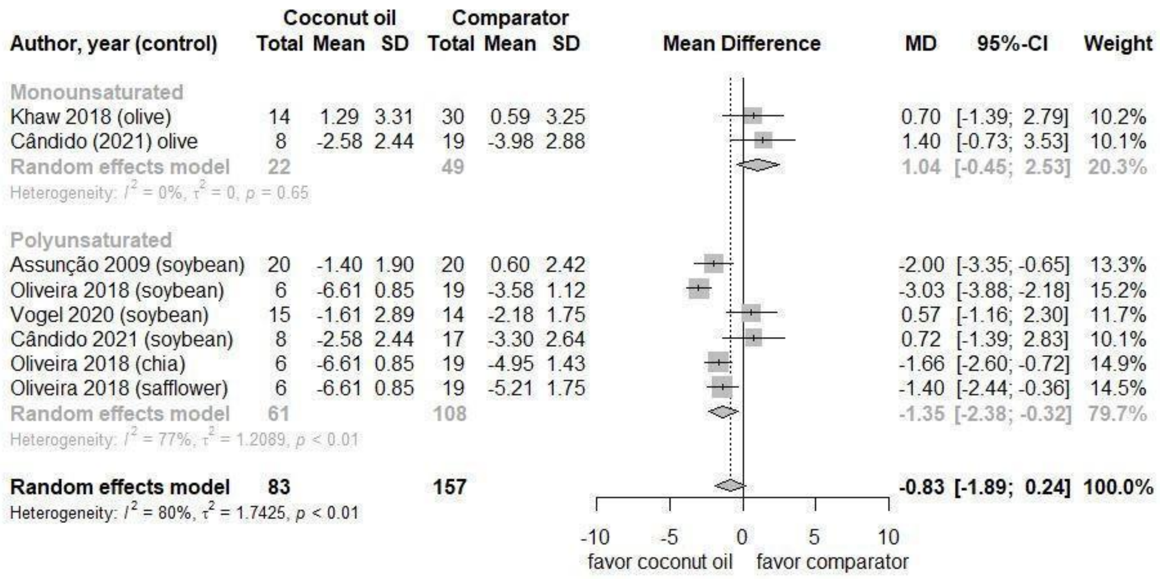

Figure S10. Forest plot of randomized controlled clinical trials investigating the effect in waist circumference (cm) of coconut oil versus olive oil

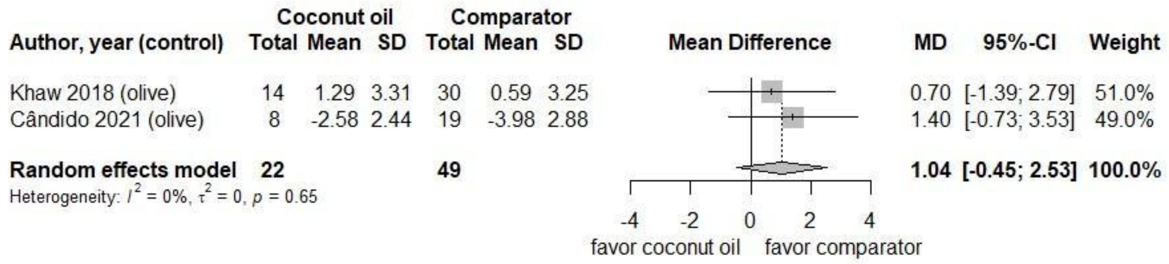

Figure S11. Forest plot of randomized controlled clinical trials investigating the effect in waist circumference (cm) of coconut oil versus soybean oil

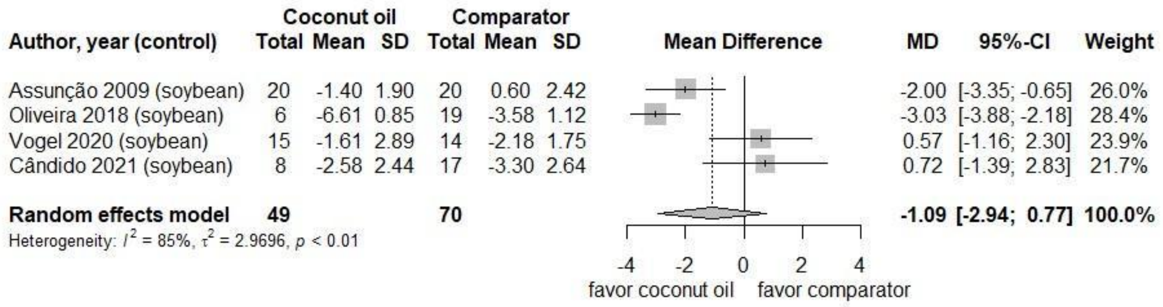

Figure S12. Forest plot of randomized controlled clinical trials investigating the effect in waist circumference (cm) of coconut oil versus other oils when analyzing studies carried out in women

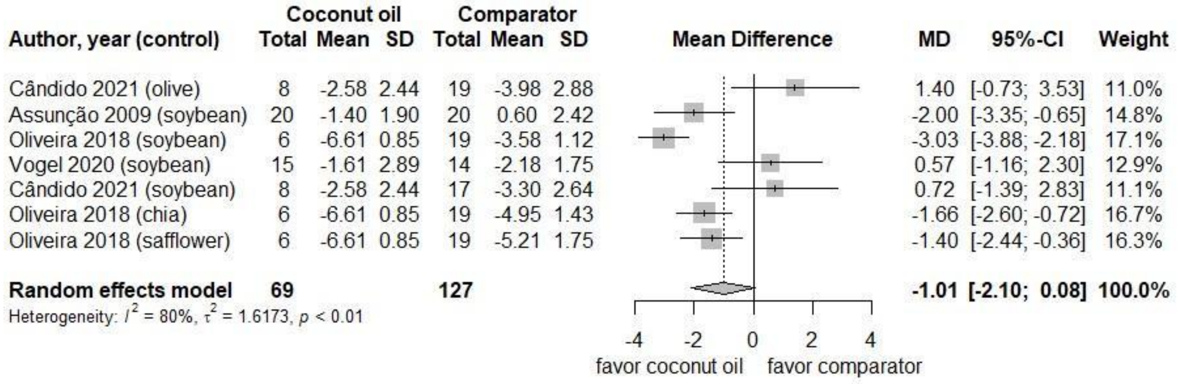

Figure S13. Forest plot of randomized controlled clinical trials investigating the effect in waist circumference (cm) of coconut oil versus other oils when analyzing studies conducted in Brazil

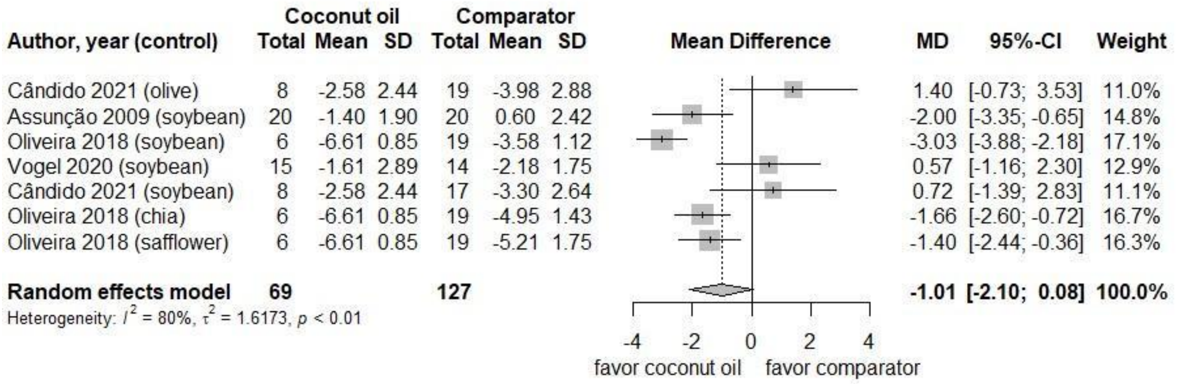

Figure S14. Forest plot of randomized controlled clinical trials investigating the effect in waist circumference (cm) of coconut oil versus other oils or/fat in patients with overweight/obesity

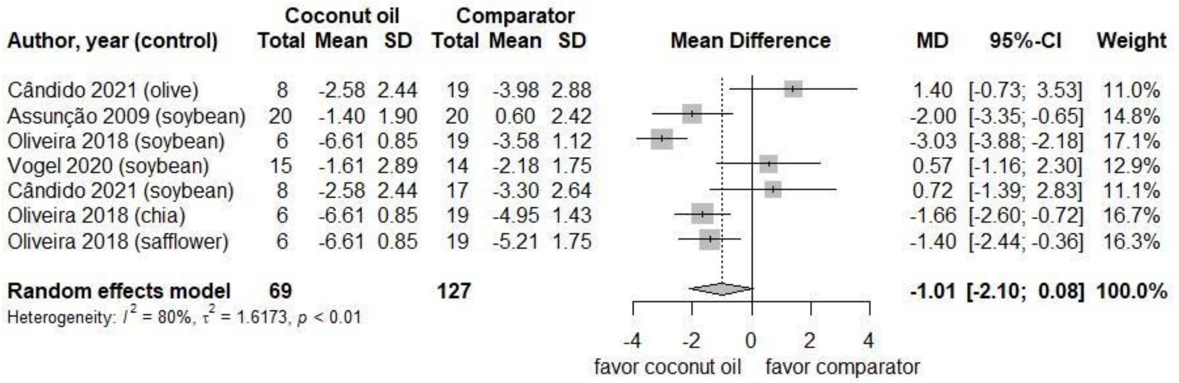

Figure S15 - Forest plot of randomized controlled clinical trials investigating the effect in waist circumference (cm) of coconut oil versus other oils/fats in studies including co-intervention

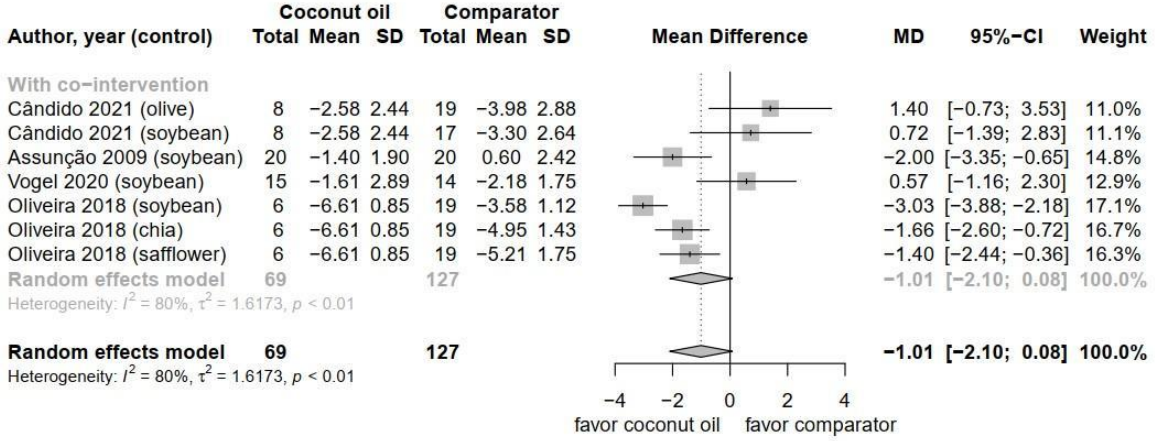

Figure S16. Forest plot of the randomized controlled clinical trials investigating the effects in % body fat of coconut oil intake in comparison to other oils/fat

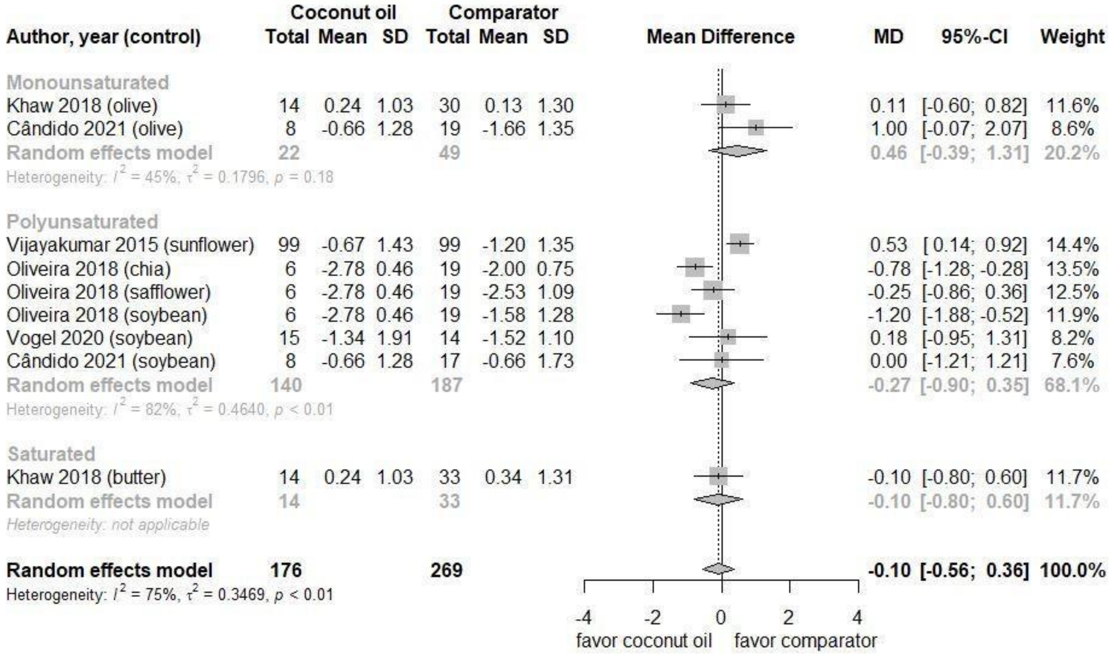

Figure S17. Forest plot of randomized controlled clinical trials investigating the effect in % body fat of coconut oil intake vs PUFA and MUFA rich oils

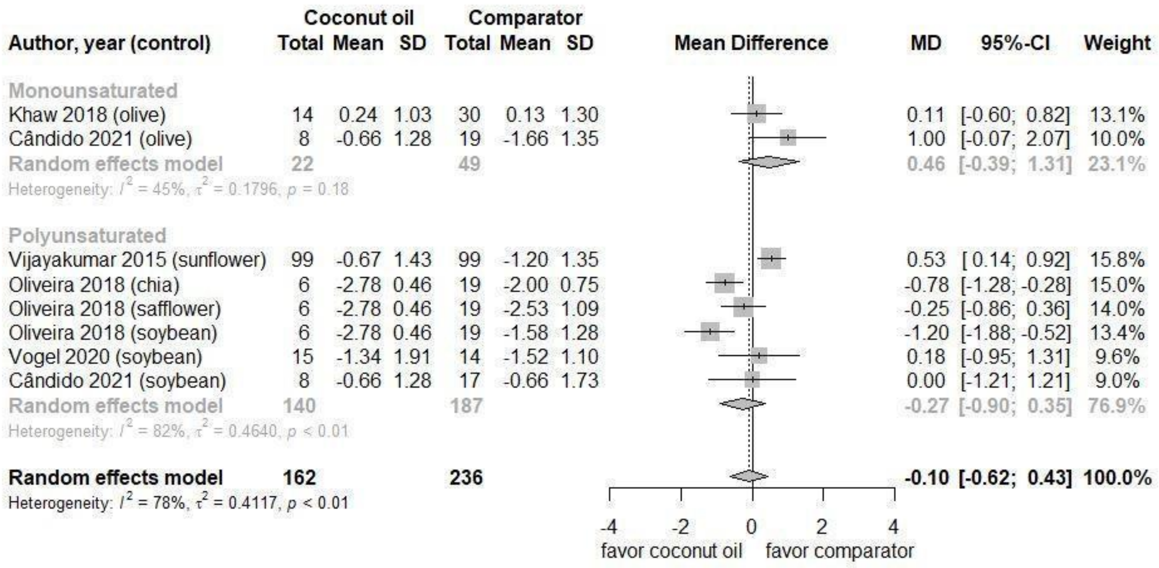

Figure S18. Forest plot of randomized controlled clinical trials investigating the effects in LDL-C (mg/dL) of coconut oil intake vs PUFA and MUFA rich oils

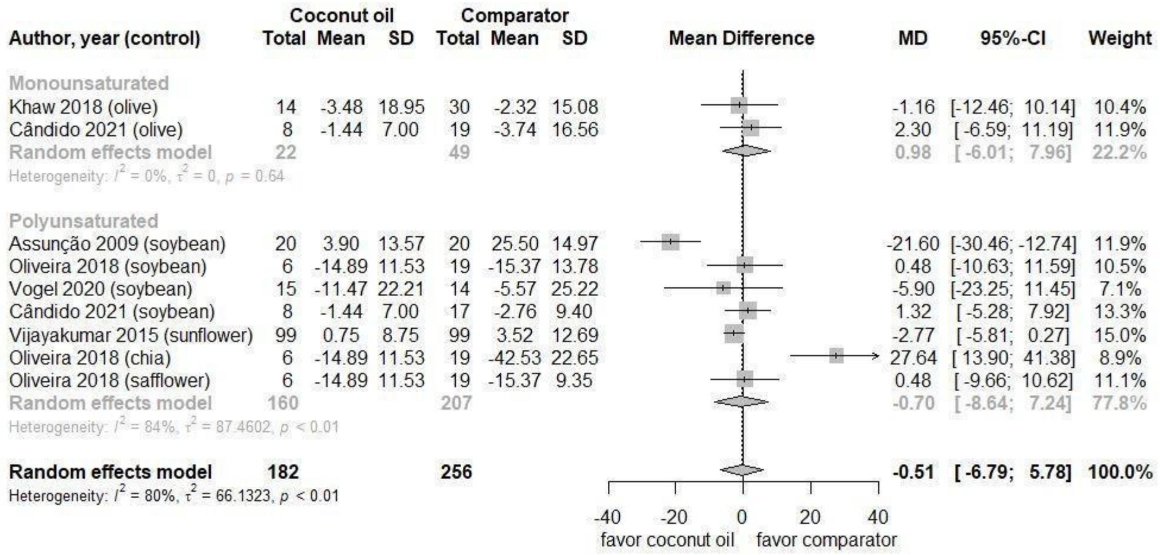

Figure S19. Forest plot of randomized controlled clinical trials investigating the effect in LDL-C (mg/dL) of coconut oil versus olive oil

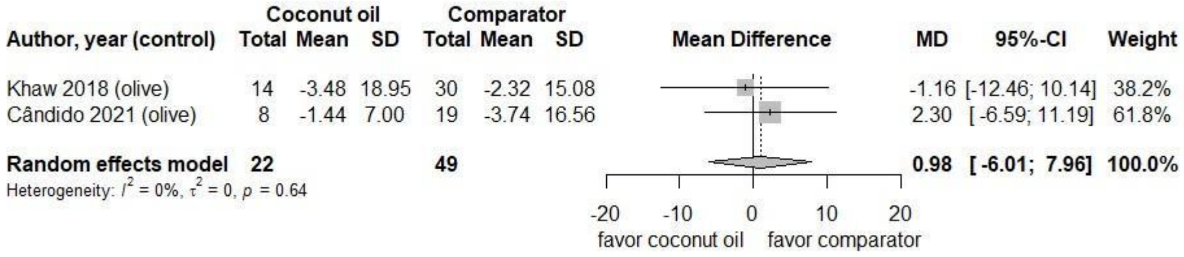

Figure S20. Forest plot of randomized controlled clinical trials investigating the effect in LDL-C (mg/dL) of coconut oil versus soybean oil

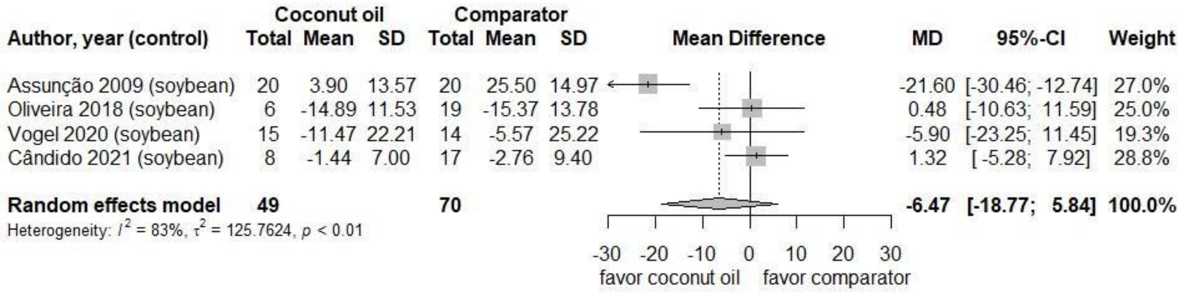

Figure S21. Forest plot of randomized controlled clinical trials investigating the effect in LDL-C (mg/dL) of coconut oil versus other oils when analyzing studies carried out in women

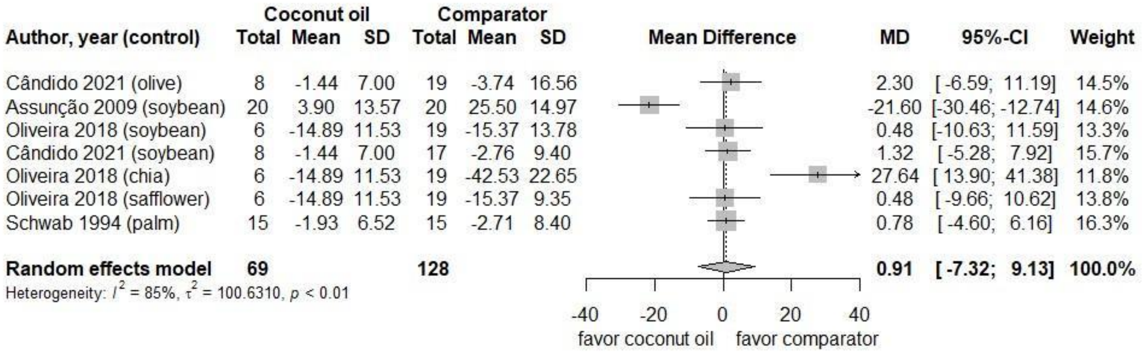

Figure S22. Forest plot of randomized controlled clinical trials investigating the effect in LDL-C (mg/dL) of coconut oil versus other oils when analyzing studies conducted in Brazil in LDL-C

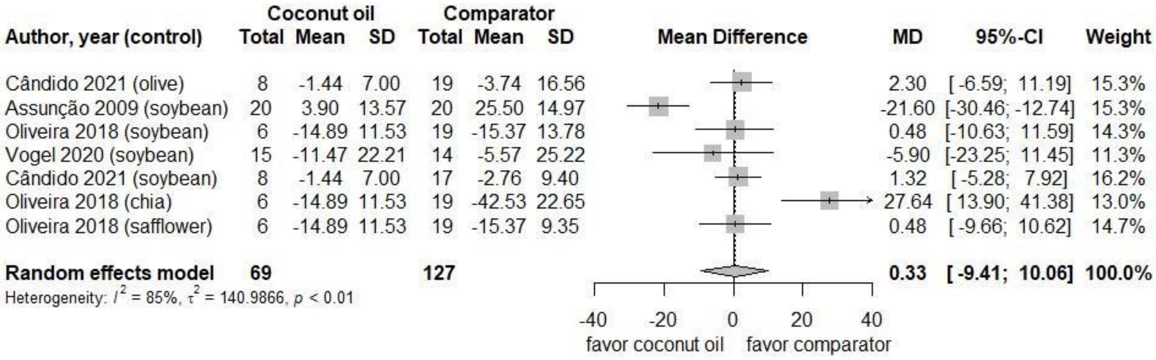

Figure S23. Forest plot of randomized controlled clinical trials investigating the effect in LDL-C (mg/dL). of coconut oil versus other oils or/fat in patients with overweight/obesity

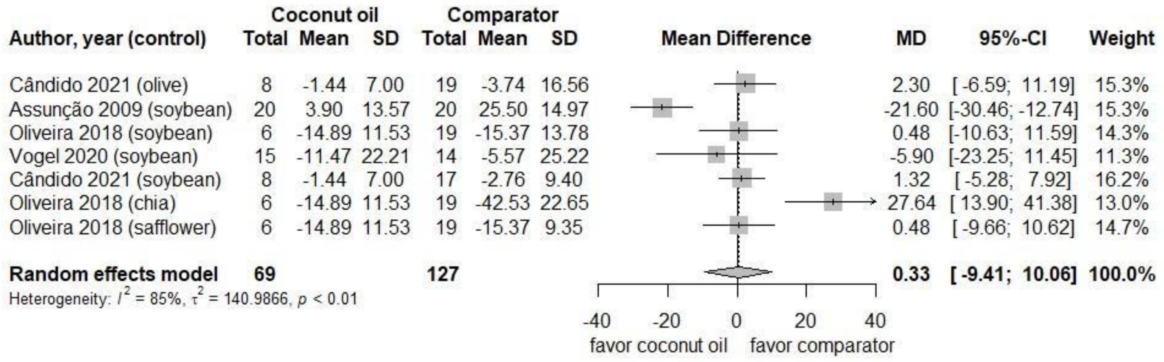

Figure S24. Forest plot of randomized controlled clinical trials investigating the effect in LDL-C (mg/dL) of coconut oil versus other oils or fat without a long term study (Vijayakumar et al)

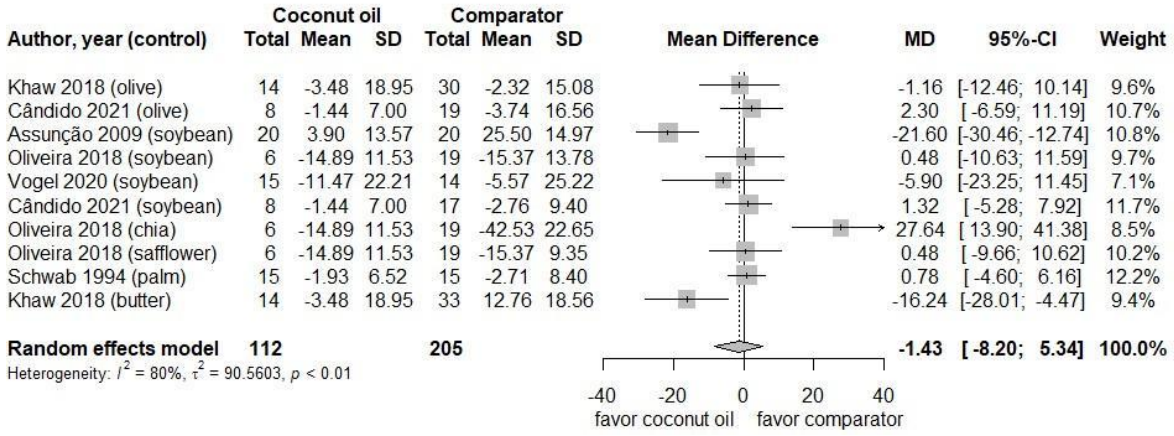

Figure S25. Forest plot of randomized controlled clinical trials investigating the effect in LDL-C (mg/dL) of coconut oil versus other oils or fat with co-intervention

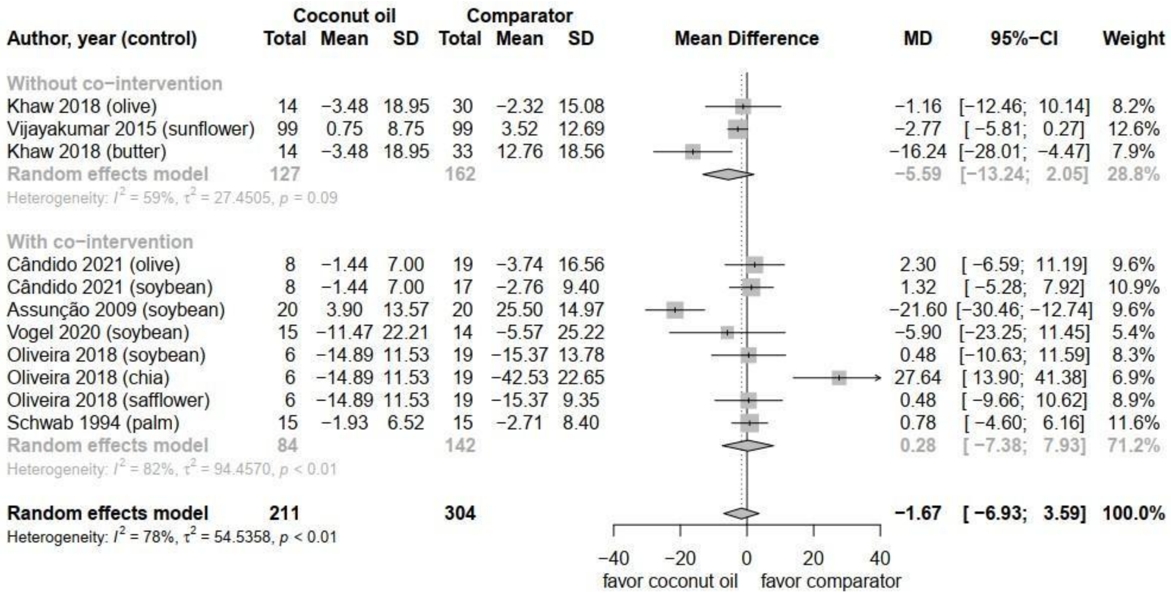

Figure S26. Forest plot of randomized controlled clinical trials investigating the effects in HDL-C (mg/dL) of coconut oil intake vs PUFA and MUFA rich oils

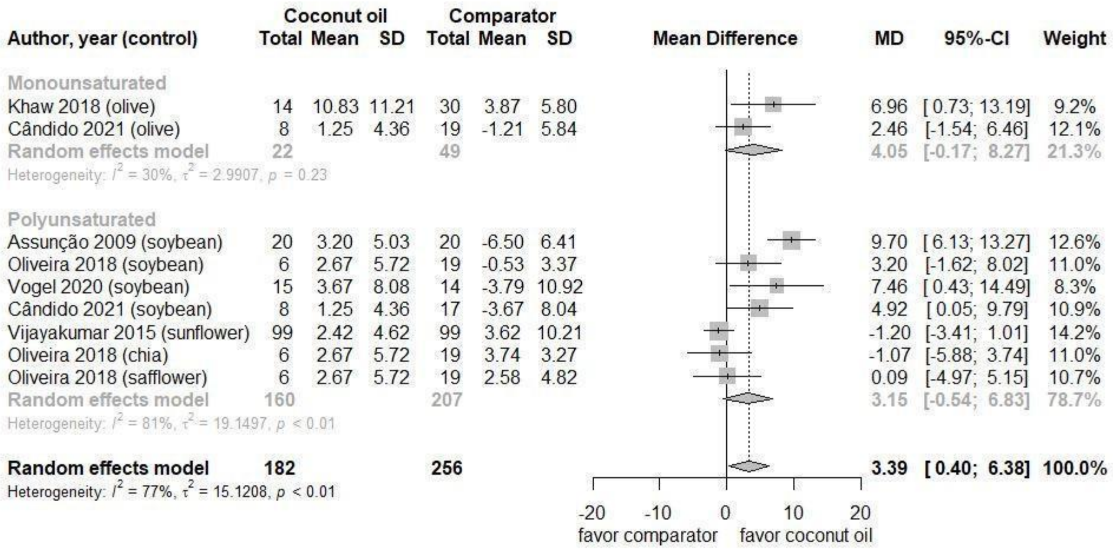

Figure S27. Forest plot of randomized controlled clinical trials investigating the effect in HDL-C (mg/dL) of coconut oil versus olive oil

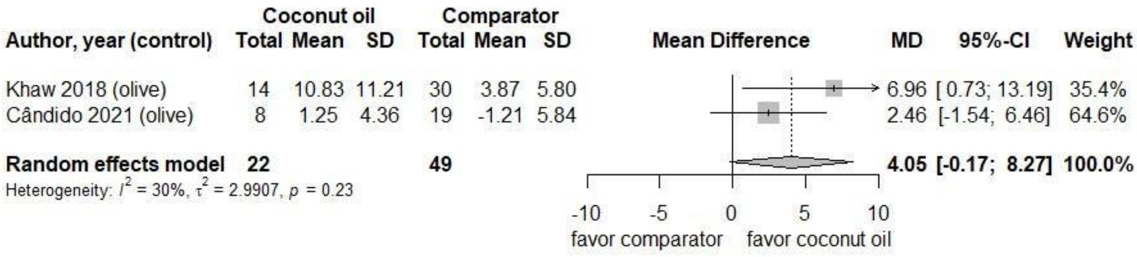

Figure S28. Forest plot of randomized controlled clinical trials investigating the effect in HDL-C (mg/dL) of coconut oil versus soybean oil

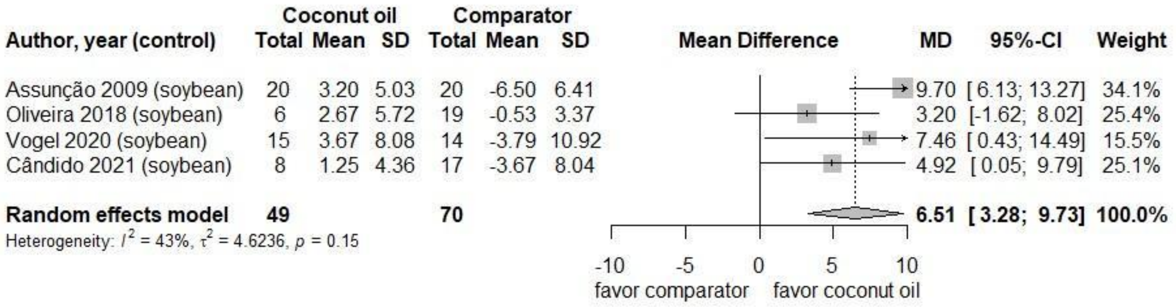

Figure S29. Forest plot of randomized controlled clinical trials investigating the effect in HDL-C (mg/dL) of coconut oil versus other oils when analyzing studies carried out in women

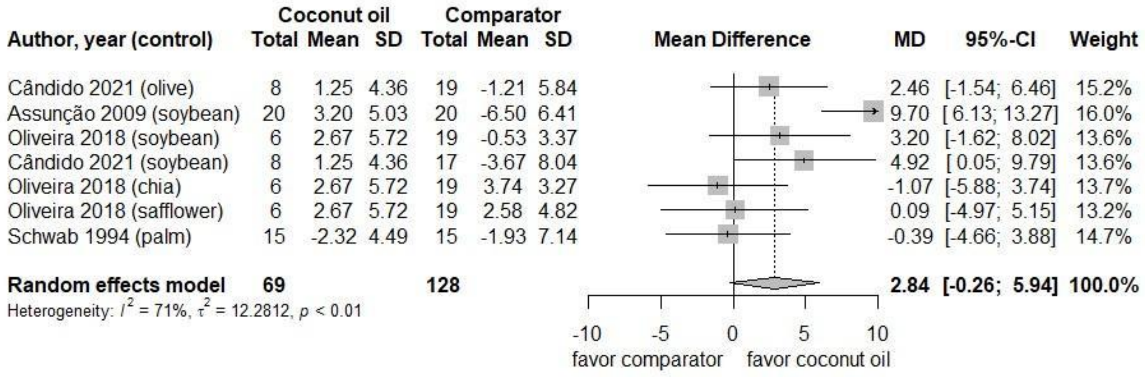

Figure S30. Forest plot of randomized controlled clinical trials investigating the effect in HDL-C (mg/dL) of coconut oil versus other oils when analyzing studies conducted in Brazil

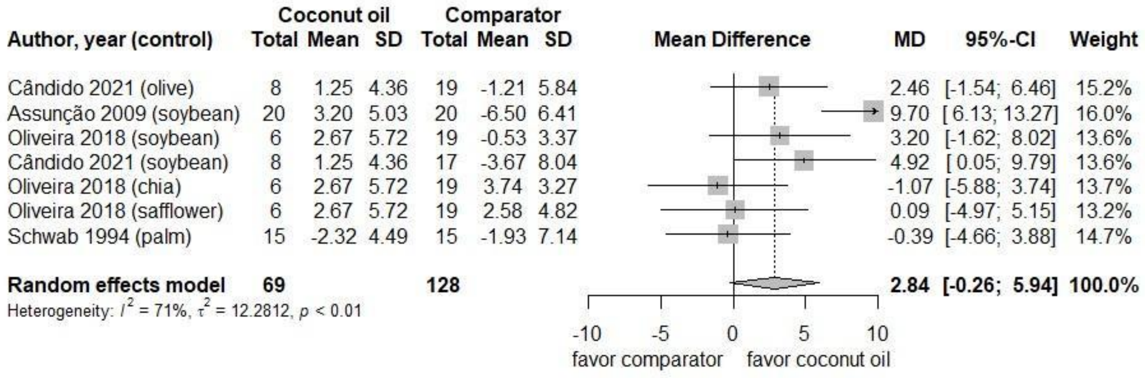

Figure S31. Forest plot of randomized controlled clinical trials investigating the effect in HDL-C (mg/dL) of coconut oil versus other oils or fat in patients with overweight/obesity

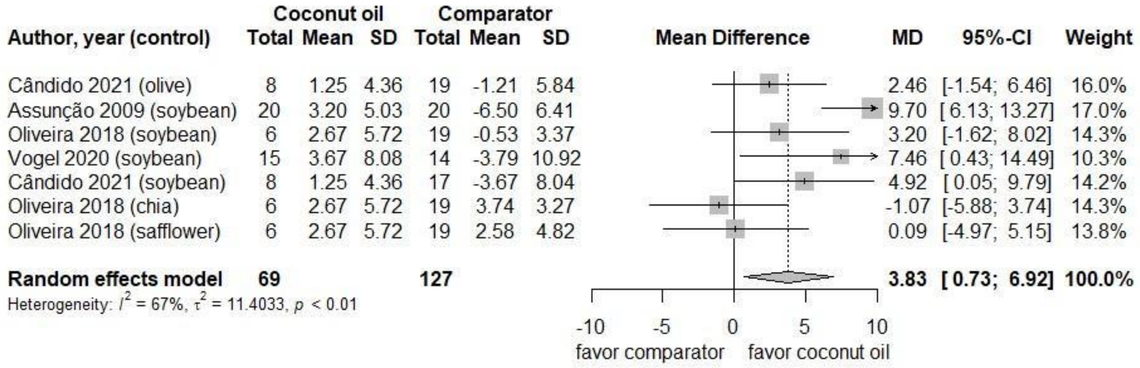

Figure S32. Forest plot of randomized controlled clinical trials investigating the effect in HDL-C (mg/dL) of coconut oil versus other oils or fat without a long term study (Vijayakumar et al)

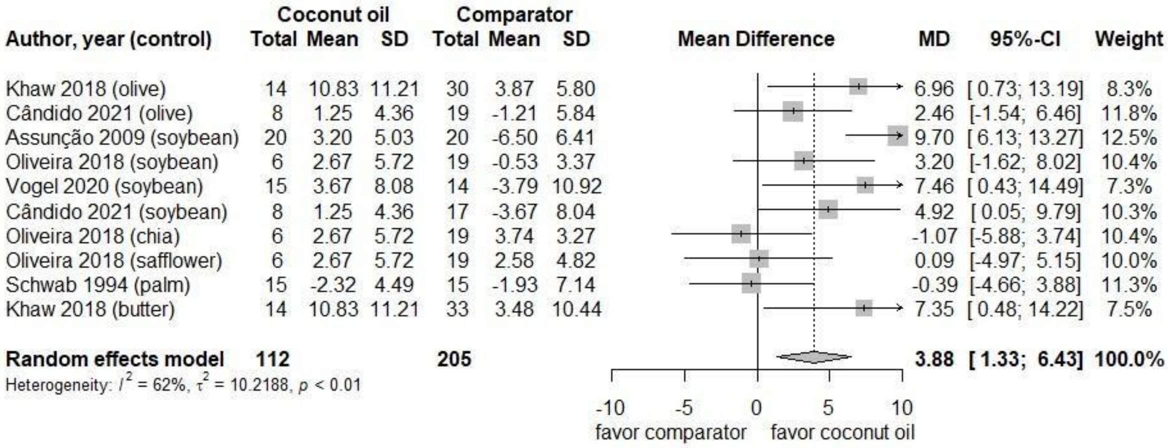

Figure S33. Forest plot of randomized controlled clinical trials investigating the effect in HDL-C (mg/dL) of coconut oil versus other oils or fat with co-intervention

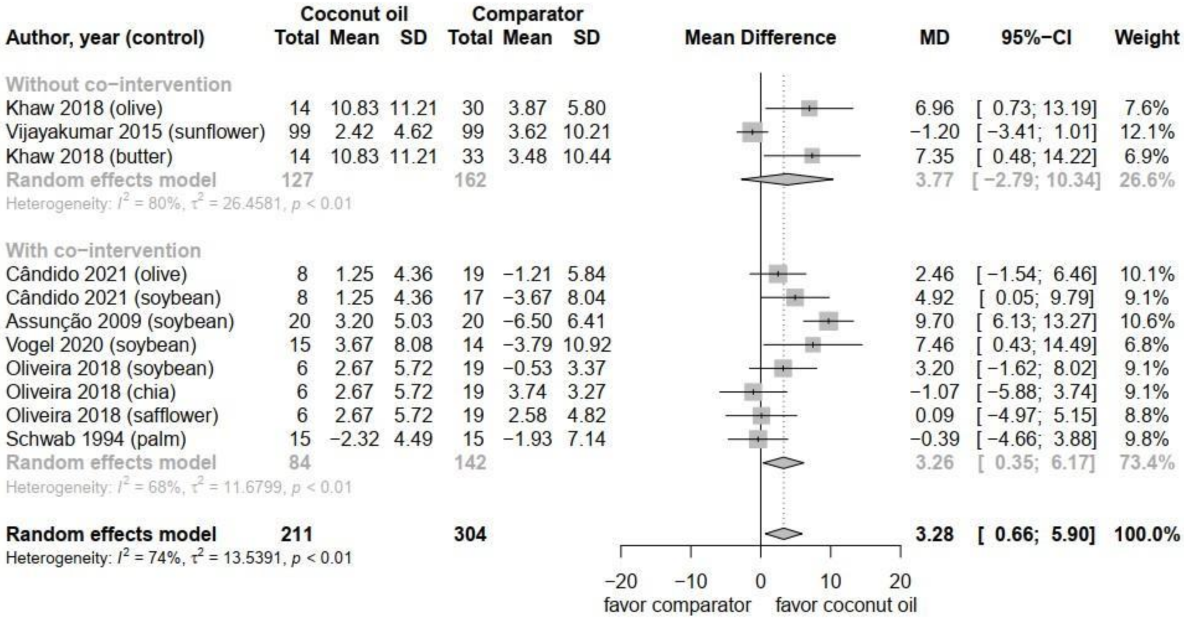

Figure S34. Forest plot of randomized controlled clinical trials investigating the effects in TG (mg/dL) of coconut oil intake vs PUFA and MUFA rich oils

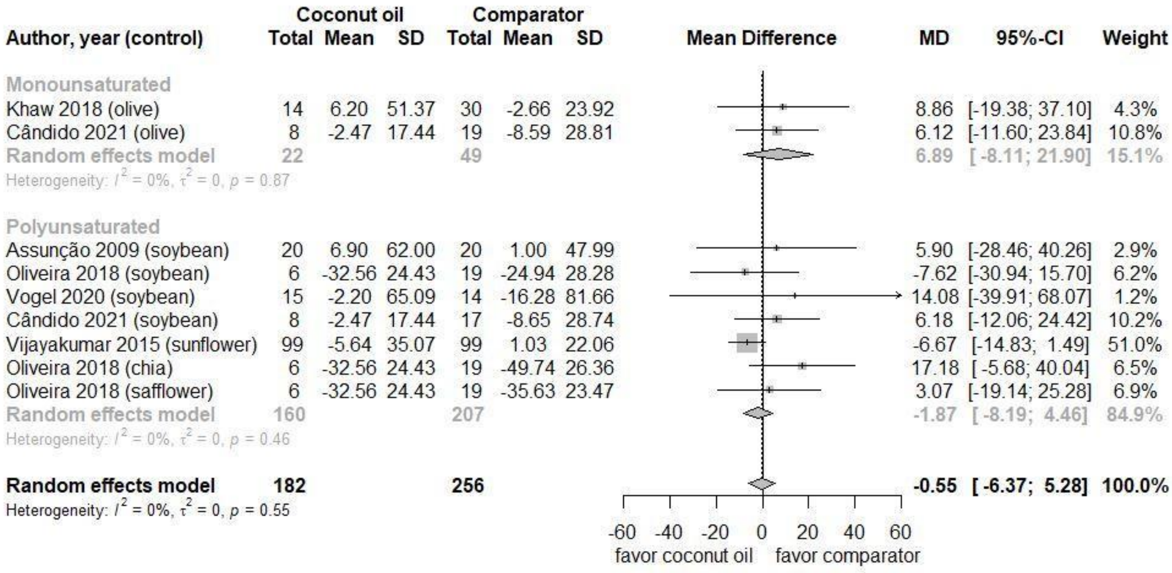

Figure S35. Forest plot of randomized controlled clinical trials investigating the effect in TG (mg/dL) of coconut oil versus olive oil

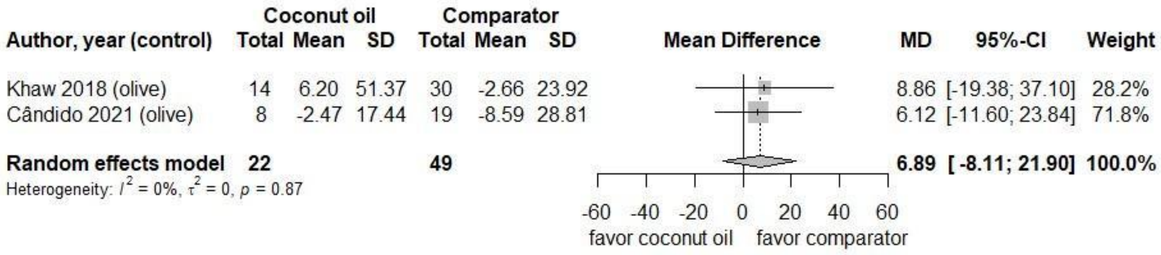

Figure S36. Forest plot of randomized controlled clinical trials investigating the effect in TG (mg/dL) of coconut oil versus soybean oil

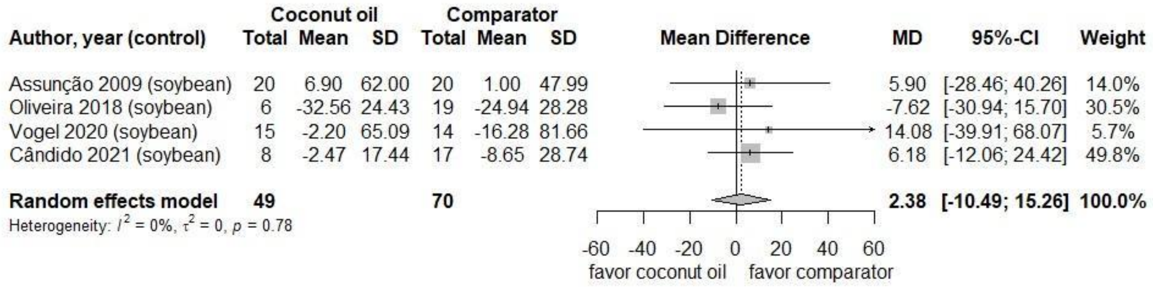

Figure S37. Forest plot of randomized controlled clinical trials investigating the effect in TG (mg/dL) of coconut oil versus other oils when analyzing studies carried out in women

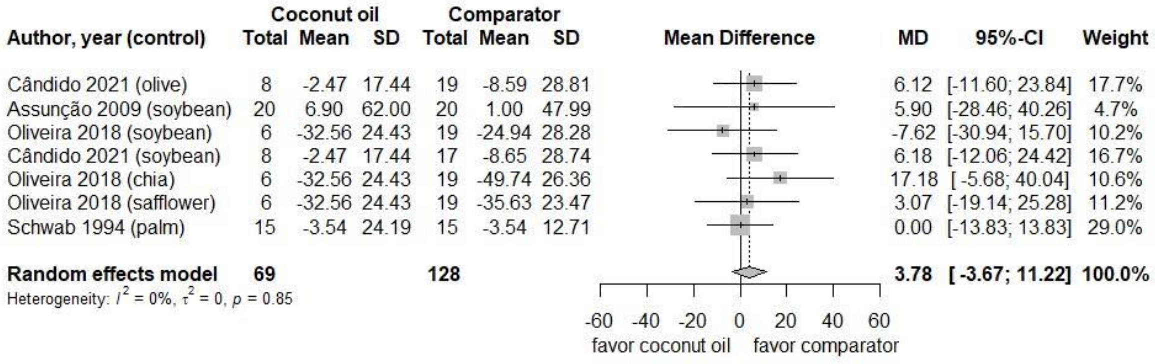

Figure S38. Forest plot of randomized controlled clinical trials investigating the effect in TG (mg/dL) of coconut oil versus other oils when analyzing studies conducted in Brazil

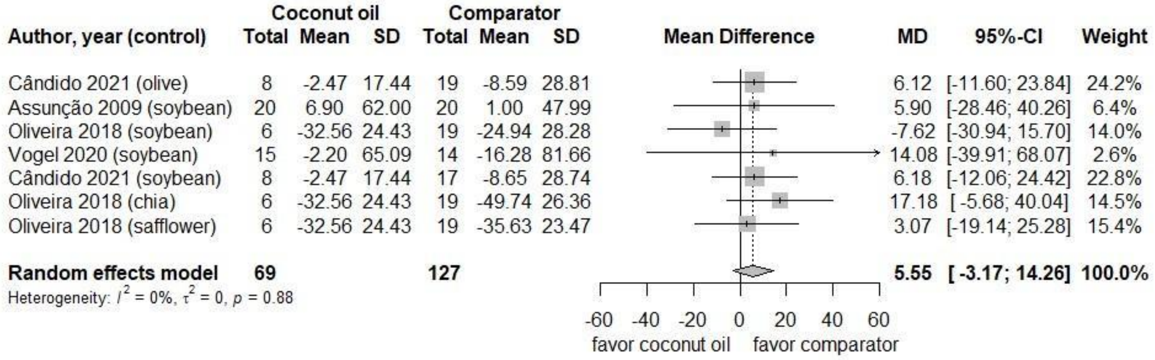

Figure S39. Forest plot of randomized controlled clinical trials investigating the effect in TG (mg/dL) of coconut oil versus other oils or fat in patients with overweight/obesity

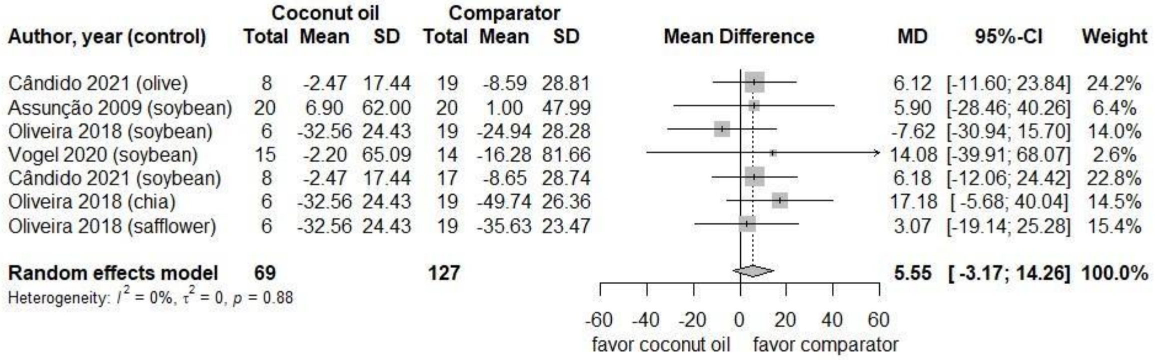

Figure S40. Forest plot of randomized controlled clinical trials investigating the effect in TG (mg/dL) of coconut oil versus other oils or fat without a long term study (Vijayakumar et al)

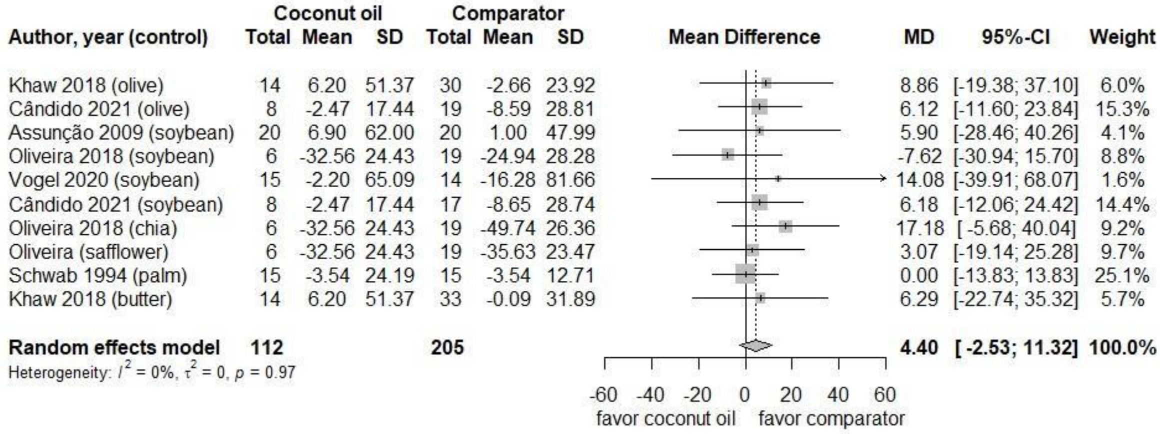

Figure S41. Forest plot of randomized controlled clinical trials investigating the effect in TG (mg/dL) of coconut oil versus other oils or fat with co-intervention

	Coconut oil			Comparator					
Author, year (control)	Total	Mean	SD	Total	Mean	SD	MD	95%−CI	Weight
Without co−intervention									
Khaw 2018 (olive)	14	6.20	51.37	30	−2.66	23.92	8.86	[−19.38; 37.10]	3.5%
Vijayakumar 2015 (sunflower)	99	−5.64	35.07	99	1.03	22.06	−6.67	[−14.83; 1.49]	41.9%
Khaw 2018 (butter)	14	6.20	51.37	33	−0.09	31.89	6.29	[−22.74; 35.32]	3.3%
Random effects model	127			162			−4.67	[−12.24; 2.90]	48.7%
Heterogeneity: $I^2 = 0\%$, $\tau^2 = 0$, $p = 0.44$									
With co−intervention									
Cândido 2021 (olive)	8	−2.47	17.44	19	−8.59	28.81	6.12	[−11.60; 23.84]	8.9%
Cândido 2021 (soybean)	8	−2.47	17.44	17	−8.65	28.74	6.18	[−12.06; 24.42]	8.4%
Assunção 2009 (soybean)	20	6.90	62.00	20	1.00	47.99	5.90	[−28.46; 40.26]	2.4%
Vogel 2020 (soybean)	15	−2.20	65.09	14	−16.28	81.66	14.08	[−39.91; 68.07]	1.0%
Oliveira 2018 (soybean)	6	−32.56	24.43	19	−24.94	28.28	−7.62	[−30.94; 15.70]	5.1%
Oliveira 2018 (chia)	6	−32.56	24.43	19	−49.74	26.36	17.18	[−5.68; 40.04]	5.3%
Oliveira 2018 (safflower)	6	−32.56	24.43	19	−35.63	23.47	3.07	[−19.14; 25.28]	5.7%
Schwab 1994 (palm)	15	−3.54	24.19	15	−3.54	12.71	0.00	[−13.83; 13.83]	14.6%
Random effects model	84			142			3.97	[−3.41; 11.34]	51.3%
Heterogeneity: $I^2 = 0\%$, $\tau^2 = 0$, $p = 0.90$									
**Random effects model**	**211**			**304**			**−0.24**	**[−5.52; 5.04]**	**100.0%**
Heterogeneity: $I^2 = 0\%$, $\tau^2 = 0$, $p = 0.72$									

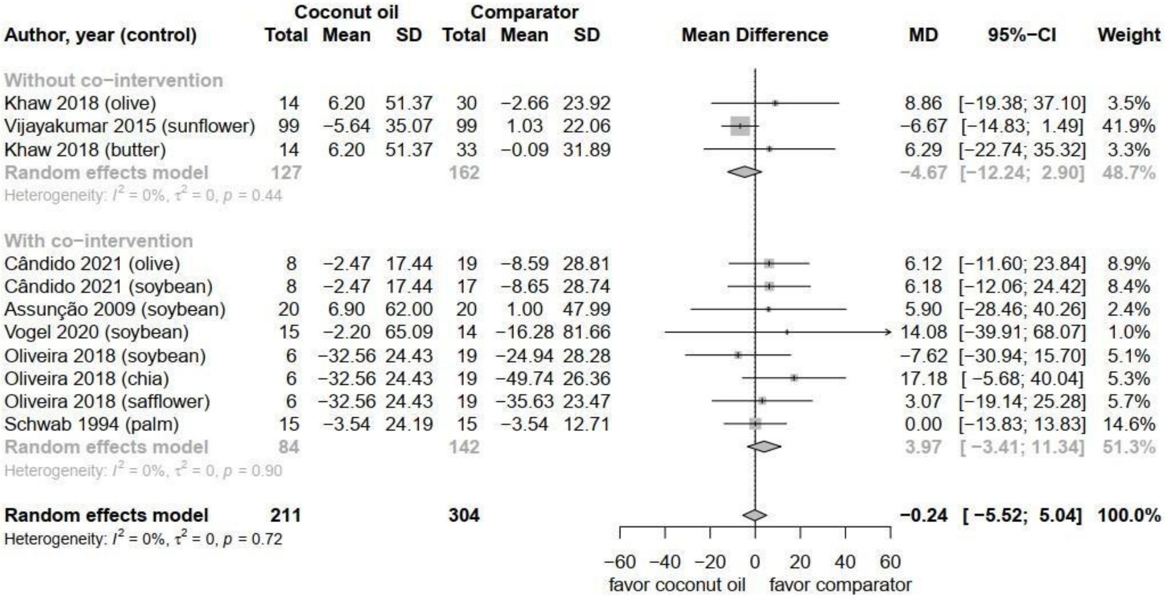

Figure S42: RoB 2.0 risk of bias in RCTs assessing the effects of coconut oil in the lipid profile

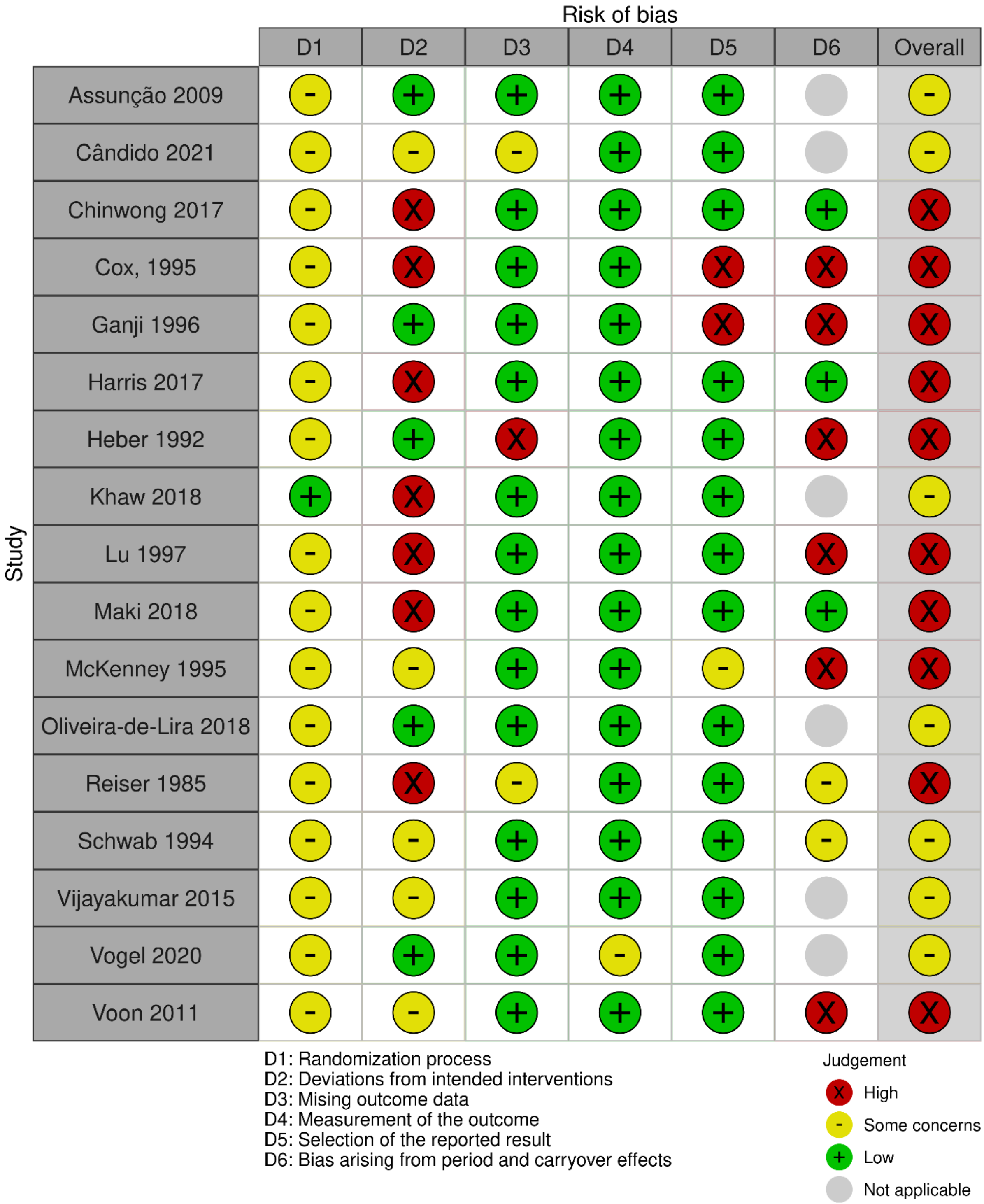

Figure S43: RoB 2.0 risk of bias in RCTs assessing the effects of coconut oil in the anthropometric profile

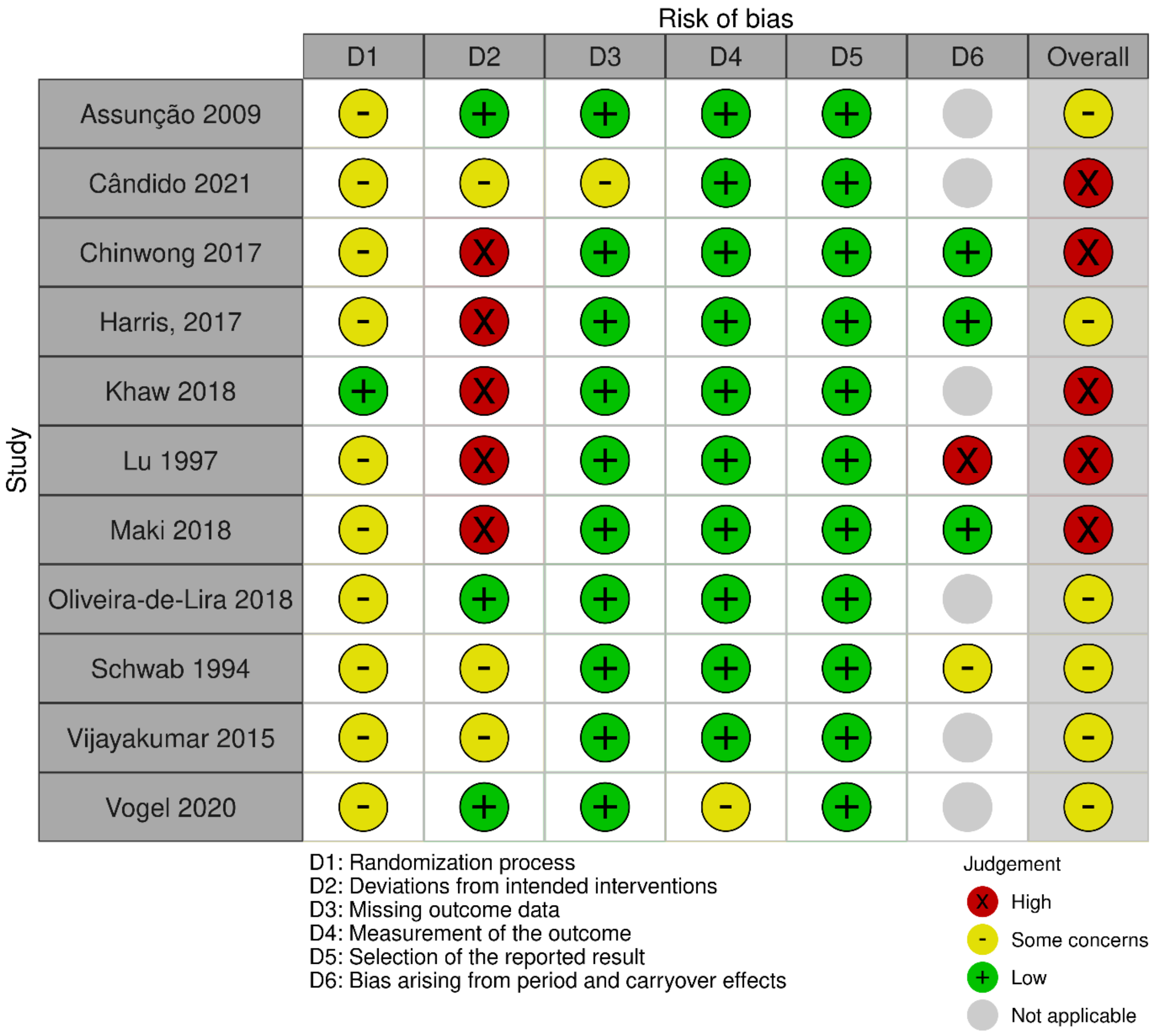

Figure S44: RoB 2.0 risk of bias in RCTs assessing the effects of coconut oil in the glycemic profile

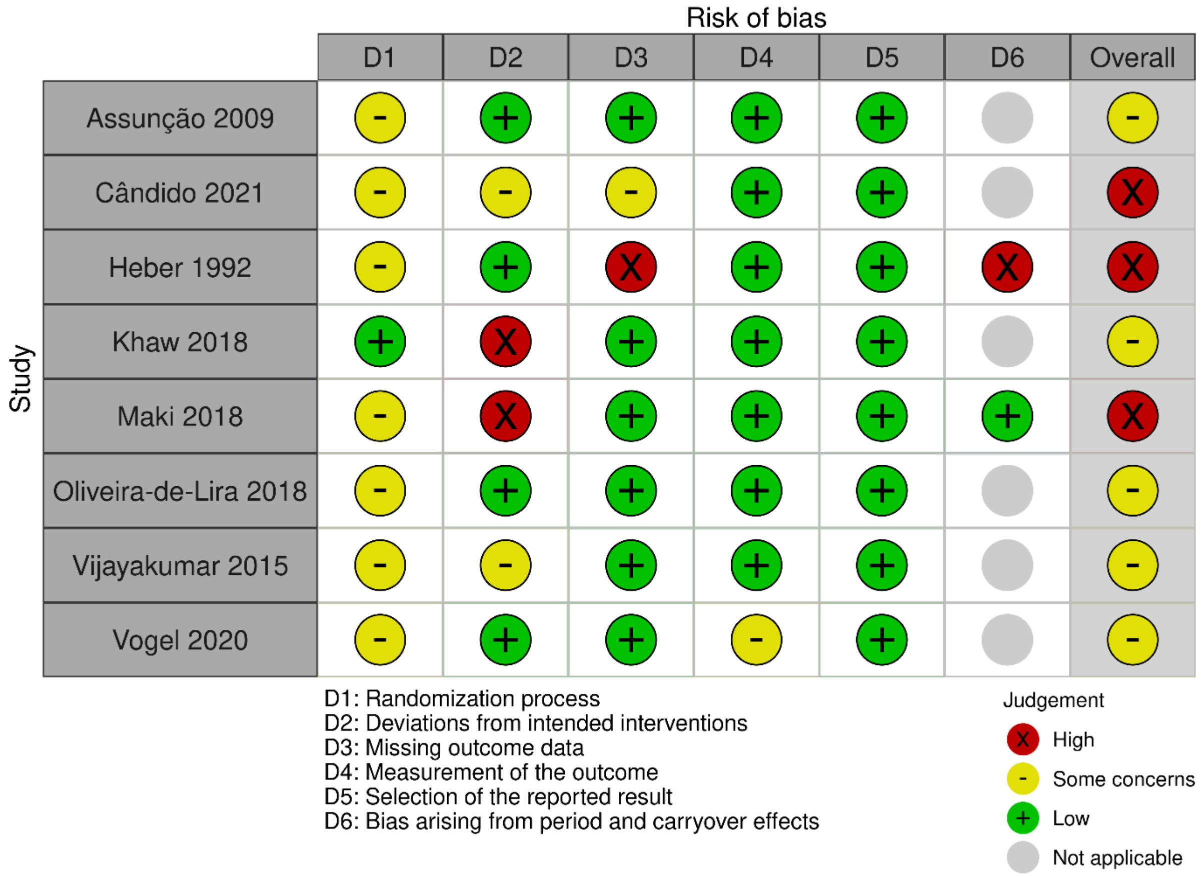

Figure S45: RoB 2.0 risk of bias in RCTs assessing the effects of coconut oil in blood pressure

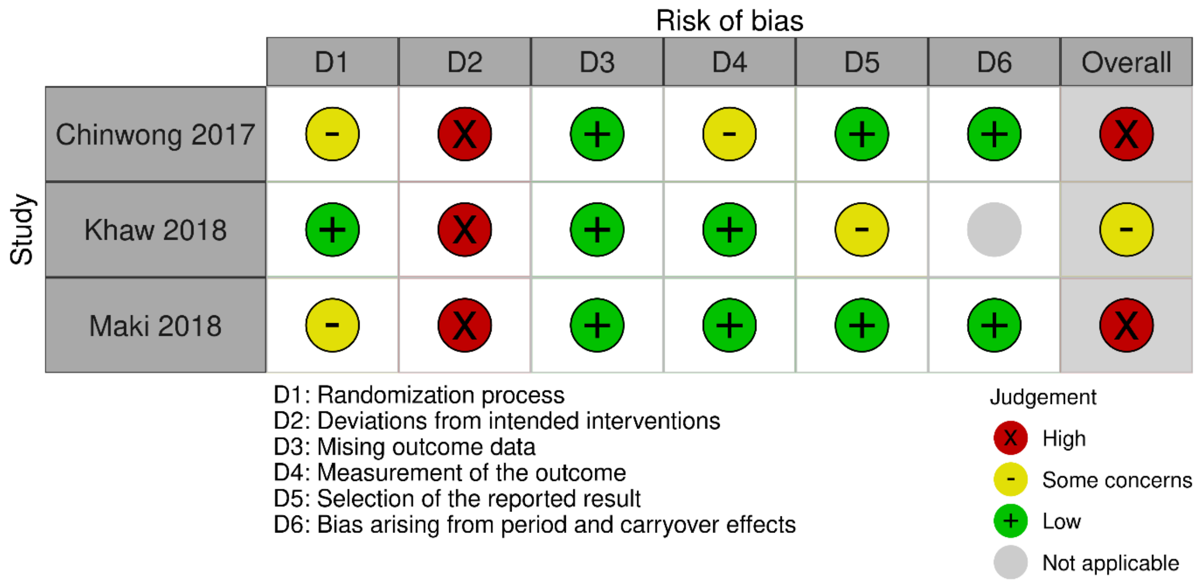

Figure S46: RoB 2.0 risk of bias in RCTs assessing the effects of coconut oil in the inflammatory profile

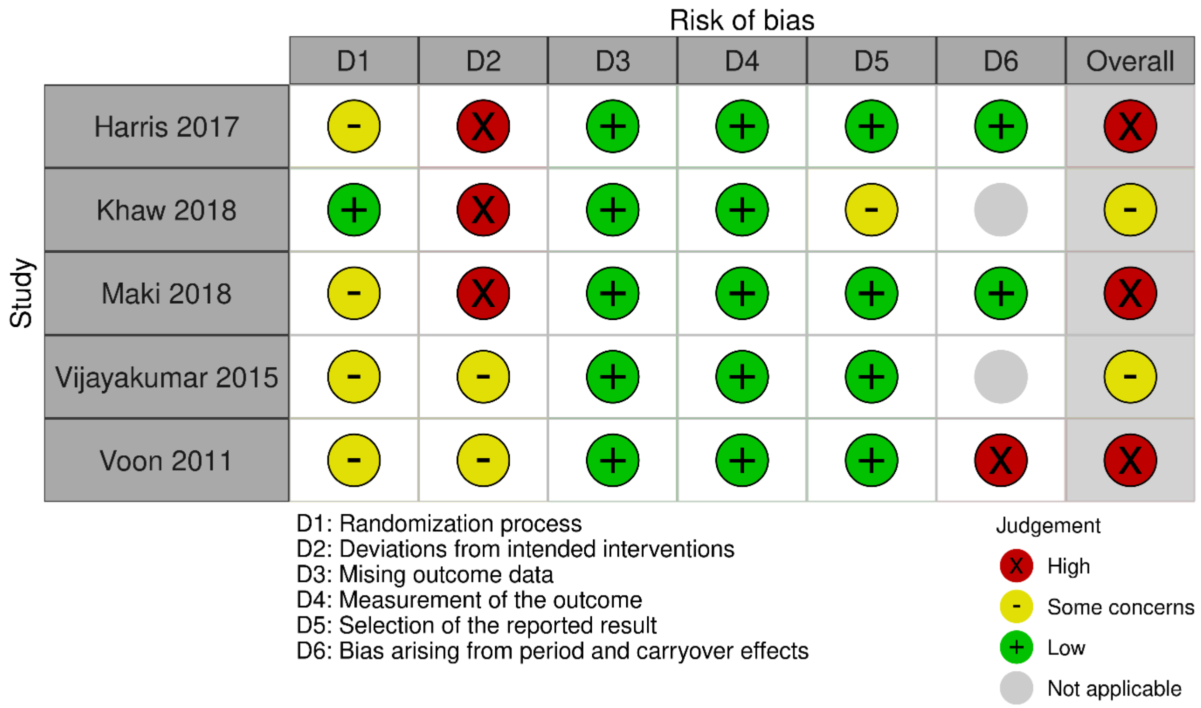

PRISMA 2020 Checklist

Section and Topic	Item #	Checklist item	Location where item is reported
**TITLE**			
Title	1	Identify the report as a systematic review.	Pg. 1
**ABSTRACT**			
Abstract	2	See the PRISMA 2020 for Abstracts checklist.	Pg. 2, 3
**INTRODUCTION**			
Rationale	3	Describe the rationale for the review in the context of existing knowledge.	Pg. 4, 5
Objectives	4	Provide an explicit statement of the objective(s) or question(s) the review addresses.	Pg. 5
**METHODS**			
Eligibility criteria	5	Specify the inclusion and exclusion criteria for the review and how studies were grouped for the syntheses.	Pg. 7, 8
Information sources	6	Specify all databases, registers, websites, organisations, reference lists and other sources searched or consulted to identify studies. Specify the date when each source was last searched or consulted.	Pg. 6
Search strategy	7	Present the full search strategies for all databases, registers and websites, including any filters and limits used.	Pg. 6
Selection process	8	Specify the methods used to decide whether a study met the inclusion criteria of the review, including how many reviewers screened each record and each report retrieved, whether they worked independently, and if applicable, details of automation tools used in the process.	Pg. 6
Data collection process	9	Specify the methods used to collect data from reports, including how many reviewers collected data from each report, whether they worked independently, any processes for obtaining or confirming data from study investigators, and if applicable, details of automation tools used in the process.	Pg. 6
Data items	10a	List and define all outcomes for which data were sought. Specify whether all results that were compatible with each outcome domain in each study were sought (e.g. for all measures, time points, analyses), and if not, the methods used to decide which results to collect.	Pg. 6, 7
	10b	List and define all other variables for which data were sought (e.g. participant and intervention characteristics, funding sources). Describe any assumptions made about any missing or unclear information.	Pg. 6, 7
Study risk of bias assessment	11	Specify the methods used to assess risk of bias in the included studies, including details of the tool(s) used, how many reviewers assessed each study and whether they worked independently, and if applicable, details of automation tools used in the process.	Pg. 7
Effect measures	12	Specify for each outcome the effect measure(s) (e.g. risk ratio, mean difference) used in the synthesis or presentation of results.	Pg. 8
Synthesis methods	13a	Describe the processes used to decide which studies were eligible for each synthesis (e.g. tabulating the study intervention characteristics and comparing against the planned groups for each synthesis (item #5)).	Pg. 7
	13b	Describe any methods required to prepare the data for presentation or synthesis, such as handling of missing summary statistics, or data conversions.	Pg. 8
	13c	Describe any methods used to tabulate or visually display results of individual studies and syntheses.	Pg. 8

Section and Topic	Item #	Checklist item	Location where item is reported
	13d	Describe any methods used to synthesize results and provide a rationale for the choice(s). If meta-analysis was performed, describe the model(s), method(s) to identify the presence and extent of statistical heterogeneity, and software package(s) used.	Pg. 8
	13e	Describe any methods used to explore possible causes of heterogeneity among study results (e.g. subgroup analysis, meta-regression).	Pg. 9
	13f	Describe any sensitivity analyses conducted to assess robustness of the synthesized results.	Pg. 9
Reporting bias assessment	14	Describe any methods used to assess risk of bias due to missing results in a synthesis (arising from reporting biases).	Pg. 7
Certainty assessment	15	Describe any methods used to assess certainty (or confidence) in the body of evidence for an outcome.	Pg. 7, 8
**RESULTS**			
Study selection	16a	Describe the results of the search and selection process, from the number of records identified in the search to the number of studies included in the review, ideally using a flow diagram.	Pg. 9 and fig. 1
	16b	Cite studies that might appear to meet the inclusion criteria, but which were excluded, and explain why they were excluded.	Figure 1 and Table S1
Study characteristics	17	Cite each included study and present its characteristics.	Pg. 9, 10 and table 1
Risk of bias in studies	18	Present assessments of risk of bias for each included study.	Pg. 15 and supplementary material
Results of individual studies	19	For all outcomes, present, for each study: (a) summary statistics for each group (where appropriate) and (b) an effect estimate and its precision (e.g. confidence/credible interval), ideally using structured tables or plots.	Figures 2 and 3
Results of syntheses	20a	For each synthesis, briefly summarise the characteristics and risk of bias among contributing studies.	Pg. 10-15
	20b	Present results of all statistical syntheses conducted. If meta-analysis was done, present for each the summary estimate and its precision (e.g. confidence/credible interval) and measures of statistical heterogeneity. If comparing groups, describe the direction of the effect.	Figures 2 and 3
	20c	Present results of all investigations of possible causes of heterogeneity among study results.	Pg. 9
	20d	Present results of all sensitivity analyses conducted to assess the robustness of the synthesized results.	Pg. 9-15
Reporting biases	21	Present assessments of risk of bias due to missing results (arising from reporting biases) for each synthesis assessed.	Pg. 15 and Figures S42-S46
Certainty of evidence	22	Present assessments of certainty (or confidence) in the body of evidence for each outcome assessed.	Pg. 15 and Table S7
**DISCUSSION**			
Discussion	23a	Provide a general interpretation of the results in the context of other evidence.	Pg. 15, 16
	23b	Discuss any limitations of the evidence included in the review.	Pg. 15-21

Section and Topic	Item #	Checklist item	Location where item is reported
	23c	Discuss any limitations of the review processes used.	Pg. 20, 21
	23d	Discuss implications of the results for practice, policy, and future research.	Pg. 21
**OTHER INFORMATION**			
Registration and protocol	24a	Provide registration information for the review, including register name and registration number, or state that the review was not registered.	Pg. 3
	24b	Indicate where the review protocol can be accessed, or state that a protocol was not prepared.	Pg. 3
	24c	Describe and explain any amendments to information provided at registration or in the protocol.	NA
Support	25	Describe sources of financial or non-financial support for the review, and the role of the funders or sponsors in the review.	Pg. 23
Competing interests	26	Declare any competing interests of review authors.	Pg. 23
Availability of data, code and other materials	27	Report which of the following are publicly available and where they can be found: template data collection forms; data extracted from included studies; data used for all analyses; analytic code; any other materials used in the review.	NA

References

[1] Lu Z, Hendrich S, Shen N, White PJ, Cook LR. Low linolenate and commercial soybean oils diminish serum HDL cholesterol in young free-living adult females. J Am Coll Nutr.1997;16(6):562-9.

[2] Assunção ML, Ferreira HS, dos Santos AF, Cabral CR, Jr., Florêncio TM. Effects of dietary coconut oil on the biochemical and anthropometric profiles of women presenting abdominal obesity. Lipids. 2009;44:593-601. https://doi.org/10.1007/s11745-009-3306-6.

[3] Ganji V, Kies CV. Psyllium husk fiber supplementation to the diets rich in soybean or coconut oil: hypocholesterolemic effect in healthy humans. Int J Food Sci Nutr. 1996;47(2):103-10. https://doi.org/10.3109/09637489609012571.

[4] Oliveira-de-Lira L, Santos EMC, de Souza RF, Matos RJB, Silva MCD, Oliveira LDS, et al. Supplementation-Dependent Effects of Vegetable Oils with Varying Fatty Acid Compositions on Anthropometric and Biochemical Parameters in Obese Women. Nutrients. 2018;10. https://doi.org/10.3390/nu10070932.

[5] Khaw KT, Sharp SJ, Finikarides L, Afzal I, Lentjes M, Luben R, et al. Randomised trial of coconut oil, olive oil or butter on blood lipids and other cardiovascular risk factors in healthy men and women. BMJ Open. 2018;8:e020167. https://doi.org/10.1136/bmjopen-2017-020167.

[6] Voon PT, Ng TK, Lee VK, Nesaretnam K. Diets high in palmitic acid (16:0), lauric and myristic acids (12:0 + 14:0), or oleic acid (18:1) do not alter postprandial or fasting plasma homocysteine and inflammatory markers in healthy Malaysian adults. Am J Clin Nutr. 2011;94(6):1451-7. https://doi.org/10.3945/ajcn.111.020107.

[7] McKenney JM, Proctor JD, Wright Jr JT, Kolinski RJ, Elswick Jr RK, Coaker JS. The effect of supplemental dietary fat on plasma cholesterol levels in lovastatin-treated hypercholesterolemic patients. Pharmacotherapy. 1995; 15(5 I):565-72. https://doi.org/10.1002/j.1875-9114.1995.tb02864.x.

[8] Harris M, Hutchins A, Fryda L. The impact of virgin coconut oil and high-oleic safflower oil on body composition, lipids, and inflammatory markers in postmenopausal women. Journal of Medicinal Food. 2017;20(4):345-51. https://doi.org/10.1089/jmf.2016.0114.

[9] Maki KC, Hasse W, Dicklin MR, Bell M, Buggia MA, Cassens ME, et al. Corn Oil Lowers Plasma Cholesterol Compared with Coconut Oil in Adults with Above-Desirable Levels of Cholesterol in a Randomized Crossover Trial. J Nutr. 2018;148(10):1556-63. https://doi.org/ 10.1093/jn/nxy156.

[10] Vogel CE, Crovesy L, Rosado EL, Soares-Mota M. Effect of coconut oil on weight loss and metabolic parameters in men with obesity: a randomized controlled clinical trial. Food Funct. 2020;11(7): 6588-6594. https://doi.org/10.1039/d0fo00872a.

[11] Heber D, Ashley JM, Solares ME, Wang HJ. The effects of a palm-oil enriched diet on plasma lipids and lipoproteins in healthy young men. Nutrition Research. 1992;12(SUPPL):S53-S9. https://doi.org/10.1016/S0271-5317(05)80450-6.

[12] Cox C, Mann J, Sutherland W, Chi. Effects of coconut oil, butter, and safflower oil on lipids and lipoproteins in persons with moderately elevated cholesterol levels. Journal of Lipid Research [Internet]. 1995;36.

[13] Cândido TLN, da Silva LE, Cândido FG, Valente FJ, da Silva JS, Lopes DRG, et al. Effect of the ingestion of vegetable oils associated with energy-restricted normo fat diet on intestinal microbiota and permeability in overweight women. Food Res Int. 2021;139:109951. https://doi.org/10.1016/j.foodres.2020.109951.

[14] Vijayakumar M, Vasudevan DM, Sundaram KR, Krishnan S, Vaidyanathan K, Nandakumar S, et al. A randomized study of coconut oil versus sunflower oil on cardiovascular risk factors in patients with stable coronary heart disease. Indian Heart J. 2016;68(4): 498-506. https://doi.org/10.1016/j.ihj.2015.10.384.

[15] Chinwong S, Chinwong D, Mangklabruks. Daily consumption of virgin coconut oil increases high-density lipoprotein cholesterol levels in healthy volunteers: a randomized crossover trial. Evid Based Complement Alternat Med. 2017;2017:7251562. https://doi.org/10.1155/2017/7251562.
